# Gene-based whole genome sequencing meta-analysis of 250 circulating proteins in three isolated European populations

**DOI:** 10.1016/j.molmet.2022.101509

**Published:** 2022-04-30

**Authors:** Arthur Gilly, Lucija Klaric, Young-Chan Park, Grace Png, Andrei Barysenka, Joseph A. Marsh, Emmanouil Tsafantakis, Maria Karaleftheri, George Dedoussis, James F. Wilson, Eleftheria Zeggini

**Affiliations:** 1 Institute of Translational Genomics, Helmholtz Zentrum München – German Research Center for Environmental Health, Ingolstaedter Landstr. 1, 85764 Neuherberg, Germany; 2 MRC Human Genetics Unit, Institute of Genetics and Cancer, University of Edinburgh, Western General Hospital, Edinburgh, EH4 2XU, UK; 3 TUM School of Medicine, Technical University of Munich and Klinikum Rechts der Isar, Ismaninger Straße 22, 8167 Munich, Germany; 4 Anogia Medical Centre, 74051 Anogia, Greece; 5 Echinos Medical Centre, 67300 Echinos, Greece; 6 Department of Nutrition and Dietetics, School of Health Science and Education, Harokopio University of Athens, 70, El. Venizelou ave. 17671, Kallithea, Greece; 7 Centre for Global Health Research, Usher Institute, University of Edinburgh, Teviot Place, Edinburgh, EH8 9AG, UK

**Keywords:** Whole-genome sequencing, Proteomics, Association studies, Gene-based tests

## Abstract

**Objective:**

Deep sequencing offers unparalleled access to rare variants in human populations. Understanding their role in disease is a priority, yet prohibitive sequencing costs mean that many cohorts lack the sample size to discover these effects on their own. Meta-analysis of individual variant scores allows the combination of rare variants across cohorts and study of their aggregated effect at the gene level, boosting discovery power. However, the methods involved have largely not been field-tested. In this study, we aim to perform the first meta-analysis of gene-based rare variant aggregation optimal tests, applied to the human cardiometabolic proteome.

**Methods:**

Here, we carry out this analysis across MANOLIS, Pomak and ORCADES, three isolated European cohorts with whole-genome sequencing (total N = 4,422). We examine the genetic architecture of 250 proteomic traits of cardiometabolic relevance. We use a containerised pipeline to harmonise variant lists across cohorts and define four sets of qualifying variants. For every gene, we interrogate protein-damaging variants, exonic variants, exonic and regulatory variants, and regulatory only variants, using the CADD and Eigen scores to weigh variants according to their predicted functional consequence. We perform single-cohort rare variant analysis and meta-analyse variant scores using the SMMAT package.

**Results:**

We describe 5 rare variant pQTLs (RV-pQTL) which pass our stringent significance threshold (7.45 × 10^−11^) and quality control procedure. These were split between four *cis* signals for MARCO, TEK, MMP2 and MPO, and one *trans* association for GDF2 in the SERPINA11 gene. We show that the *cis*-MPO association, which was not detectable using the single-point data alone, is driven by 5 missense and frameshift variants. These include rs140636390 and rs119468010, which are specific to MANOLIS and ORCADES, respectively. We show how this kind of signal could improve the predictive accuracy of genetic factors in common complex disease such as stroke and cardiovascular disease.

**Conclusions:**

Our proof-of-concept study demonstrates the power of gene-based meta-analyses for discovering disease-relevant associations complementing common-variant signals by incorporating population-specific rare variation.

## Highlights

• Meta-analysis of gene-based variant scores leverages population-specific allelic heterogeneity.
• We study rare variants influencing 250 circulating serum proteins in two Greek population isolates.
• 4 cis and 1 trans coding variant signals are study-wide significant and pass quality control.
• Five rare coding variants influence protein expression of myeloperoxidase (MPO).
• Different isolates are enriched for different rare coding MPO variants.

## 1 Introduction

Cardiovascular disease is the leading cause of death globally, with an estimated 17.9 million fatalities each year. A large number of genetic susceptibility factors have been identified through population-scale genome-wide association studies (GWAS) [1]. However, the exact pathways involved remain elusive, given that most associations arise in noncoding regions of the genome [2].

Proteins are one of the key mediating molecules in complex disease, and measuring their levels in peripheral blood can inform diagnosis, prognosis and treatment [3]. Examining the overlap between genetic variants affecting complex disease and protein levels can therefore pinpoint biologically-relevant disease pathways. Population-scale association studies of the proteome have discovered a growing number of such protein quantitative trait loci (pQTLs) either within or close to the protein-coding gene (*cis*), or distal to it (*trans*), with a particular focus on cardiovascular outcomes [[4], [5], [6], [7], [8], [9], [10]]. Similar to what is observed in complex trait association studies, cross-population meta-analyses have conferred unprecedented discovery power to such analyses [8,9].

Although they increase sample sizes markedly, these studies typically use imputed GWAS data. They are therefore limited to common and low-frequency variants. We have previously shown that both coding and noncoding rare variants affect the peripheral proteome independently of common variant signals [11]. Meta-analysis of such rare variant association signals has historically been challenging without sharing individual-level genetic data, since rare variants in or around a gene can differ markedly between populations. Meta-analysis of p-values from gene-based tests is possible, but remains of limited interest in the presence of different signal architectures and allelic heterogeneity.

Recently, methods have been introduced that summarise rare variant information into meta-analysable single-variant scores and correlation matrices [12]. Unlike aggregation tests, which assume an equidirectional effect across all variants and studies, these tests are agnostic to the underlying genetic architecture. However, these methods have not been extensively field tested, and no such large meta-analysis of score-based gene-centric tests currently exists.

Here, we perform genome-wide rare variant meta-analysis across three isolated European cohorts with whole-genome sequencing (total N = 4,422). We examine the genetic architecture of 250 proteomic traits of cardiometabolic relevance from the OLINK Cardiovascular II, Cardiovascular III and Metabolism panels, and describe 5 rare variant pQTLs (RV-pQTLs). We investigate in detail a burden of coding variants in the *MPO* gene, and demonstrate a distinct contribution of the rare, isolate-specific rs119468010 and rs140636390.

## 2 Materials and methods

### 2.1 Cohort information

The HELIC (www.helic.org) study comprises the MANOLIS (Minoan Isolates) cohort, focusing on Anogia and the surrounding Mylopotamos villages on the Greek island of Crete, and the Pomak cohort, which focuses on a set of isolated mountainous villages in the North of Greece. All individuals were required to have at least one parent from the respective area to enter the study. Recruitment was primarily carried out at the village medical centers. The study includes biological sample collection for DNA extraction and lab-based blood measurements, and interview-based questionnaire filling. The phenotypes collected include anthropometric and biometric measurements, clinical evaluation data, biochemical and hematological profiles, self-reported medical history, demographic, socioeconomic and lifestyle information [11,[13], [14], [15], [16], [17], [18]] [19,20]. The study was approved by the Harokopio University Bioethics Committee and informed consent was obtained from every participant.

The Orkney Complex Disease Study (ORCADES) is a family-based study that seeks to identify genetic factors influencing cardiovascular and other disease risk in the isolated archipelago of the Orkney Isles in northern Scotland [21]. Genetic diversity in this population is decreased compared to Mainland Scotland, consistent with the high levels of endogamy historically. 2078 participants aged 16–100 years were recruited between 2005 and 2011, most having three or four grandparents from Orkney, the remainder with two Orcadian grandparents. Fasting blood samples were collected and many health-related phenotypes and environmental exposures were measured in each individual. All participants gave written informed consent and the study was approved by Research Ethics Committees in Orkney, Aberdeen (North of Scotland REC), and South East Scotland REC, NHS Lothian (reference: 12/SS/0151).

### 2.2 Sequencing and variant calling

The three cohorts were sequenced in an identical way. Genomic DNA (500 ng) from 1482, 1642, and 1352 samples for MANOLIS, Pomak and ORCADES, respectively, was subjected to standard Illumina paired-end DNA library construction. Adapter-ligated libraries were amplified by 6 cycles of PCR and subjected to DNA sequencing using the HiSeqX platform (Illumina) according to manufacturer's instructions.

Basecall files for each lane were transformed into unmapped BAMs using Illumina2BAM (https://github.com/wtsi-npg/illumina2bam), marking adaptor contamination and decoding barcodes for removal into BAM tags. PhiX control reads were mapped using BWA Backtrack [22] and were used to remove spatial artefacts. Reads were converted to FASTQ and aligned using BWA MEM 0.7.8 [23] to the hg38 reference (GRCh38) with decoys (HS38DH). The alignment was then merged into the master sample BAM file using Illumina2BAM MergeAlign. PCR and optical duplicates are marked using biobambam markduplicates (https://github.com/gt1/biobambam) and the files were archived in CRAM format.

Per-lane CRAMs were retrieved and reads pooled on a per-sample basis across all lanes to produce library CRAMs; these were each divided in 200 chunks for parallelism. GVCFs were generated using HaplotypeCaller v.3.5 from the Genome Analysis Toolkit (GATK) for each chunk [24]. All chunks were then merged at sample level, samples were then further combined in batches of 150 samples using GATK CombineGVCFs v.3.5. Variant calling was then performed on each batch using GATK GenotypeGVCFs v.3.5. The resulting variant callsets were then merged across all batches into a cohort-wide VCF file using bcftools concat.

### 2.3 Variant and sample quality control

Variant-level QC of genotype calls was performed using the Variant Quality Score Recalibration tool (VQSR) from the Genome Analysis Toolkit (GATK) v. 3.5-0-g36282e431, using a tranche threshold of 99.4% for SNPs, which provided an estimate false positive rate of 6%, and a true positive rate of 95%. For indels, we used the recommended threshold of 99%. For sample-level QC, we made extensive use of genotyping array datasets in overlapping samples, which provided sample matching information for 1,386 and 1,511 samples in MANOLIS and Pomak, respectively. In MANOLIS, a total of 25 individuals were excluded (n = 1457) based on sex checks, low concordance (pi-hat <0.8) with chip data, duplicate checks, average depth (<10x), missingness (>0.5%), and contamination (Freemix or CHIPMIX score from the verifyBamID suite [25] >5%). This number was 27 for the Pomak cohort. In case of sample duplicates, the sample with highest quality metrics (depth, freemix and chipmix score) was kept. No samples were excluded in ORCADES.

### 2.4 Proteomics

The serum levels of all proteins from three Olink panels - CVDII, CVDIII and Metabolism - were measured in MANOLIS and Pomak using Olink's proximity extension assay (PEA) technology [26]. In ORCADES, the same panels were measured in plasma. Briefly, for each assay, the binding of a unique pair of oligonucleotide-labelled antibody probes to the protein of interest results in the hybridisation of the complementary oligonucleotides, which triggers extension by DNA polymerase. DNA barcodes unique to each protein are then amplified and quantified using microfluidic real-time qPCR. Measurements are given in a natural logarithmic scale in Normalised Protein eXpression (NPX) levels, a relative quantification unit. NPX is derived by first adjusting the qPCR Ct values by an extension control, followed by an inter-plate control and a correction factor predetermined by a negative control signal. This is followed by intensity normalisation, where values for each assay are centered around its median across plates to adjust for inter-plate technical variation. Further details on the internal and external controls used can be found at http://www.olink.com. Additionally, a lower limit of detection (LOD) value is determined for each protein based on the negative control signal plus three standard deviations. In this study, NPX values that fall below the LOD were set to missing.

For MANOLIS and Pomak, we regress covariates out of the inverse-normal transformed NPX values, followed by standardisation. In ORCADES, standardisation was replaced by an additional inverse normal transformation to guard against heteroskedasticity. All covariates were regressed out of all phenotypes, irrespective of their significance in the model. Covariates included were sex, age, age squared, plate number, and mean NPX level across all proteins, per sample. We also adjusted for season, given the observed annual variability of some circulating protein levels. Given the dry Mediterranean climate of Crete, we define season of collection as hot summer or mild winter. Plate effects are partially offset by the median-centering implemented by Olink. MANOLIS and Pomak samples were plated in the order of sample collection, which results in plate and season information to be largely correlated. For ORCADES, we included age, age squared, sex, sampling month, plate number, x-y coordinate of sample on the plate, and mean NPX level across all proteins per sample [27].

In MANOLIS, we excluded 13 protein measurements across all panels with missingness or below-LOD proportion greater than 40%. BNP(B-type natriuretic peptide) was measured across all three panels, and was excluded due to high missingness in all three. 26, 2 and 14 samples failed vendor QC and were excluded from CVDII, III and META, respectively. 42 samples were excluded due to missing age.

In Pomak we excluded 15 proteins and 49, 6 and 13 samples in CVDII, III and META, respectively, due to vendor QC failure. Missing ages were imputed by regressing all proteins that were non-missing in the 24 samples without age on sex and age, and identifying those for which the coefficient P-value was lower than a Bonferroni-corrected threshold of 2 × 10^−4^. We then regress age on all these proteins and sex in all non-missing samples.

ORCADES, like MANOLIS, was assayed on a previous version of the CVDIII panel, which means CCL22 was present instead of GP6. CCL22 was excluded upon vendor recommendation due to generally unreliable results. 23 samples from the CVDII panel and 15 from META were excluded in ORCADES due to vendor QC. In ORCADES, age, age-squared, sex, month of the year, plate number, row and column on the plate, as well as average NPX value were used as covariates during normalisation. 15 samples were removed due to absence of covariates.

In total, 277 proteins were measured across the 3 cohorts. 22 proteins were excluded for failing QC in at least one cohort (Supplementary Table 1).

### 2.5 Single-point association and meta-analysis

Single-point association is not used as discovery in this study, and only serves to elucidate the structure of rare variant signals.

Association was performed using a linear mixed model for all three cohorts. For MANOLIS and Pomak, we carry out single-point association using GEMMA v.0.94 ^33^. We use an empirical relatedness matrix calculated on a LD-pruned (using parameters 50-5-2 from plink [28]) variants filtered for MAF<5% and HWE p < 1 × 10^−5^ with mid-p adjustment. We further filter out variants with missingness higher than 1%, as was performed for rare variant tests. 5 proteins were excluded due to having a genomic control λ_GC_ < 0.95 or λ_GC_ > 1.05 after association, bringing the total number of analysed proteins to 250.

GEMMA v. 0.94 truncates alleles to a single character. In order to enable unambiguous meta-analysis of indels, we updated alleles in summary statistics by matching it to the VCF. More precisely, we join both files by chromosome and position, and match the alleles by frequency for biallelic SNVs. For multiallelics, we compute the difference in allele frequency between the GEMMA output MAF, which is based on samples with non-missing phenotypes, and the AF fields of each allele in the VCF, and use the alleles with the lowest difference.

In ORCADES, association was performed using GCTA v.1.93.0 beta using the MLMA algorithm [29] Using common LD-pruned variants for calculating the relatedness matrix was not sufficient, as persistent inflation was present. We assumed this was due to a different relatedness structure being expressed in rare variants, and we therefore included all sequence variants in the relatedness calculation, using 5 partitions of the autosomal genome. Following this, inflation was controlled. We use the 2011-03-25 release of METAL [30] for meta-analysis using inverse-variance based weighting. The genomic inflation factors across proteins had a mean of λ = 1.04 (σ = 0.079) post meta-analysis (Supplementary Figure 1).

### 2.6 Rare variant association

We use commit c5504b6 of the MUMMY wrapper (https://github.com/hmgu-itg/burden_testing) for rare-variant analyses, which is based on the GMMAT R package [12]. This version used GMMAT v. 1.2.0. Variant files documenting all polymorphic positions and alleles in each cohort are first produced, then merged across all cohorts to produce an input variant set. Multiallelic variants are exploded into multiple records for analysis, but are ignored by this version of GMMAT. VCFs are converted to GDS using the seqVCF2GDS function from the seqArray package.

We use protein coding genes from Gencode v.32 to define testing units [31]. Following our previously-reported analysis strategy [11,32], for each gene, we create 4 sets of qualifying variants (QV) with an upper MAF threshold of 5%, an upper missingness threshold of 1%, and their associated weights. The first set comprises all unweighted variants with an Ensembl VEP [33] predicted consequence more severe than missense. The second includes all exonic variants weighted by their CADD score, including a 50 base pair window either side of each exon. The third extracts the regulatory build from Ensembl release 98 [34], and overlaps each regulatory region with GTEx release 7 [35]. For each gene, the set of regulatory regions that overlaps with a significant eQTL variant in any tissue for that gene is defined as linked to the gene. All variants overlapping with linked regions, as well as exons, are then weighted using the Eigen score [36], which incorporates information about regulatory consequences. Finally, the fourth set of QVs includes the variants in the third set that are not exonic. CADD integrates multiple annotations into one metric by contrasting variants that survived natural selection with simulated mutations, whereas Eigen is an unsupervised method based on spectral decomposition of multiple functional annotations in coding as well as noncoding regions. We discard tests where the set of QVs does not include at least two variants. After single-cohort analyses, we meta-analyse scores using the meta-analysis function of MUMMY, which wraps the SMMAT.meta function of the GMMAT package.

All p-values reported in this article correspond to the SKAT-O optimal test [37] (O.pval as reported by SMMAT). An important parameter of this test is the *ρ* parameter, which is used to define the unified test statistic:

$$Q_{\rho }=\rho Q_{Burden}+\left(1-\rho \right)Q_{SKAT}$$

According to the above, *ρ* = 1 corresponds to a pure burden (collapsing) test, while *ρ* = 0 corresponds to a pure SKAT test. SMMAT allows to optimise this mixture parameter across a grid of values, which we define as all increments of ρ from 0 to 1.

### 2.7 Significant signals and quality control

We use a significance threshold of 7.45 × 10^−11^, which we derived by computing the effective number of traits and variants analysed [11,38]. Thresholds obtained by permutation testing for rare variant tests in the UK Biobank, on a higher number of both variants and traits, are markedly greater (2 × 10^−9^) [39], suggesting that the threshold used in this study is conservative.

779 signals passed our stringent threshold, which involved to 358 unique gene-pairs across multiple QV sets. For cases where several such sets passed the threshold, we selected the one with the lowest p-value, producing 357 signals taken forward for QC.

Since cis-pQTL signals tend to be much more strongly associated than complex traits, association peaks often extend over large multi-megabase genomic regions. We therefore exclude rare variant signals for genes that are less than 1Mbp away from the *cis* gene. 120 signals remain at this stage.

For each rare variant signal, we identify the qualifying variant with the lowest single-point meta-analysis association p-value, and add its genotypes as a covariate in the rare variant test. We exclude signals where the conditioned p-value is greater than 1 × 10^−3^, since a large p-value would indicate that the signal is driven by a single variant. We also exclude rare-variant signals where the meta-analysis rare-variant p-value is higher than at least one of the single-cohort analyses, which indicates attenuation/non-replication of a single-cohort signal. 55 signals pass this filtering step.

We further exclude cases where the rare-variant signal overlaps with a stronger single-point signal. We have previously shown that such signals can contribute independent signal at a common-variant, single-point locus, so this scenario is not indicative of a false positive. Rather, this filtering criterion was adopted to focus on signals purely arising from rare-variant meta-analysis. We exclude the *cis-*ACP6 exonic and regulatory signal, since it is known [11], and the *trans-*IL6RA signal in *UBQLN4,* since it is located 1.5 Mb away from a particularly strong *cis-*IL6RA single-point peak. Five signals pass this filtering step and are discussed in detail in the Results section.

Only one of these signals, the *cis-*MPO burden, is not also discoverable at the same threshold using the single-point meta-analysis, i.e., no single-point p-value passes the threshold of 7.45 × 10^−11^ within 1 Mb of the gene where the signal arises.

### 2.8 Association with stroke and CVD in UK biobank

#### 2.8.1 Phenotype definition

We sought to quantify the predictive accuracy of MPO-altering variants for cardiovascular diseases where this gene was previously implicated. For this, we leverage the clinical information reported in the UK Biobank [40]. We construct stroke and cardiovascular disease phenotypes based on multiple disease-relevant variables in the UK Biobank phenotype database. Since these diseases are strongly influenced by clinical and lifestyle factors, we first construct a covariate list containing sex, age, hyperlipidemia, hypertension, obesity, poor glycaemic control and smoking habits. We extract sex (field 31) and ten genetic principal components (fields 22009.0.1–10). For age, we take the maximum over all visits of the age when visited assessment centre (field 21003), since we also aggregate disease codes across all visits. For body mass index, systolic and diastolic blood pressure, glycated haemoglobin, low-density lipoprotein and triglycerides, we extract the corresponding fields (21001, 93 and 4080, 94 and 4079, 30750, 30780, 30870, respectively), and average them per individual across all visits where they were measured. We extract self-reported lipid- or blood-pressure lowering medication information (field 6177) and define hypertension as having SBP>140 or DBP>90 or taking blood pressure medication. We further define hyperlipidemia as having LDL>4.115 (third quartile) or TG > 5.6 or taking lipid-lowering medication, poor glycaemic control as having HbA1c > 64, obesity as having BMI>30. These thresholds are established in the literature [41], except for LDL where the suggested threshold of 1.8 would have assigned hyperlipidemia to most cases. Since there is no established LDL threshold for hyperlipidemia (the Institute for Quality and Efficiency in Health Care recommends 3.4), we used the third quartile to capture the top 25% of LDL measurements. For smoking, there is some confusion in the UK Biobank as to whether “ever smoked” (field 20160) was understood as “ever smoked one cigarette” or “ever were a regular smoker”. When compared to field 20116 (“smoking status”, defined as never/previous/current smoker), 27% of those who answered “never” also answered “yes” to “ever smoked”. We therefore use field 20116 as a better indicator of smoking habit and use it as a categorical variable with three levels.

For stroke, we base our definition on the Definitions of Stroke for UK Biobank Phase 1 Outcomes Adjudication v1.1. We define self-reported subarachnoid, intracerebral, ischaemic haemorrhage as having a self-reported non-cancer disease code (field 20002) equal to 1086, 1491, and 1583 respectively. We also define a general self-reported stroke that include all the above plus 1081 (unspecified stroke). We define hospital-diagnosed subarachnoid haemorrhage as having an ICD9 code of 430 or ICD10 code of I60 in any hospital episode data (HES table) or cause of death report (field 40001). Intracerebral haemorrhage is similarly defined using an ICD9 codes of 431 or ICD10 code of I61, and ischaemic stroke is defined using an ICD9 code of 434 or 436 and an ICD10 code of I63 or I64. We then define four stroke phenotypes (subarachnoid, intracerebral, ischaemic, and all) with two sub-phenotypes (self-reported or hospital diagnosis, and hospital diagnosis only).

For cardiovascular disease (CVD), we base our definition on previous literature [41,42]. For self-reported coronary heart disease (CHD), we extract field 6150 (“heart attack diagnosed by doctor”), value 1075 (heart attack) from field 20002, or self-reported operation (field 20004) including percutaneous transluminal coronary angioplasty (value 1095), coronary artery bypass graft surgery (value 1070), or triple heart bypass (value 1523). For hospital-diagnosed, we use ICD9 codes of 410–412, ICD10 codes of I21–I24 or I25.2 from HES data, as well as OPCS4 operation codes of K40–K46, K49, K50 or K75. We similarly create a CHD phenotype restricted to hospital-diagnosed CHD and one that includes self-reported data. We further define a “chronic” category for CHD by including ICD10 codes of I25.1, I25.8 and I25.9 in hospital diagnoses, resulting in four distinct CHD subphenotypes.

#### 2.8.2 Genetic scores

Genetic prediction is most effective when the effect of multiple variants is linearly combined in a polygenic score (PGS). For MPO, we first seek to replicate the score created by Phuah et al. [43], containing 15 variants which were suggestively associated at p < 1 × 10^−6^ in a previous study by Reiner et al. [44] (rs2814778, rs800292, rs12049351, rs2144300, rs9332739, rs3134931, rs1390943, rs12940923, rs2680701, rs9911753, rs8081967, rs6503905, rs28730837, rs35897051, rs6042507). We extracted these variants from the UK Biobank imputed genotype data, imputed missing genotypes using the allelic mean and created a normalised score based on the effect sizes reported in the original study. Since this work meta-analysed both serum and plasma studies, we stratified this score based on whether these variants had arisen in a plasma or serum analysis. We further sought to augment this score with recent results from large genome-wide, proteome-wide association studies. We added 14 additional SNVs reported in three large proteome-wide association efforts [[7], [8], [9]]. When a SNP was present in more than one study, we selected the score reported in the largest in terms of total (discovery and replication) sample size, as such a study will have more power to detect the true effect size. We also incorporated all independent variants at all MPO-associated locus if those were reported. We performed LD-based analysis and removed among SNVs which were tagging others in the combined score (r^2^ > 0.7) the one arising from the study with the smallest total sample size. This produced a score with 19 variants. Since the three studies contributing to this new score are all plasma-based, we also performed peak calling and independent signal discovery using the peakplotter pipeline [45] which uses conditional stepwise regression as implemented in COJO [46], on the serum-only HELIC meta-analysis using a threshold of 1 × 10^−6^, the same as used in the historical score [44]. This added 16 variants, one of which was removed because it was novel an could therefore not be found in an imputed dataset, and another because it was in strong LD with others in the score. Four further variants were monomorphic in the UK Biobank, and 10 variants therefore remained. We created versions of each of these three scores that excluded any SNV with frequency lower than 0.05, including all variants included in our gene-based test.

#### 2.8.3 Score-disease association

We then performed logistic regression of 12 stroke and CAD phenotypes, first using covariates only (PC1-10, age, sex, hypertension, hyperlipidemia, poor glycaemic control, obesity, and smoking) for our null model. Sex, age and smoking were significant predictors of all disease phenotypes, and hyperlipidemia, glycaemic control and obesity also significantly improved the model for most traits. We then included the historical score of Rainer et al. as a predictor. This MPO genetic score was a significant predictor in all CAD phenotypes, as well as for ischaemic and all stroke.

## 3 Results

### 3.1 Four cis signals and 1 trans pass QC, driven in part by isolate-specific variants

**Figure 1:**
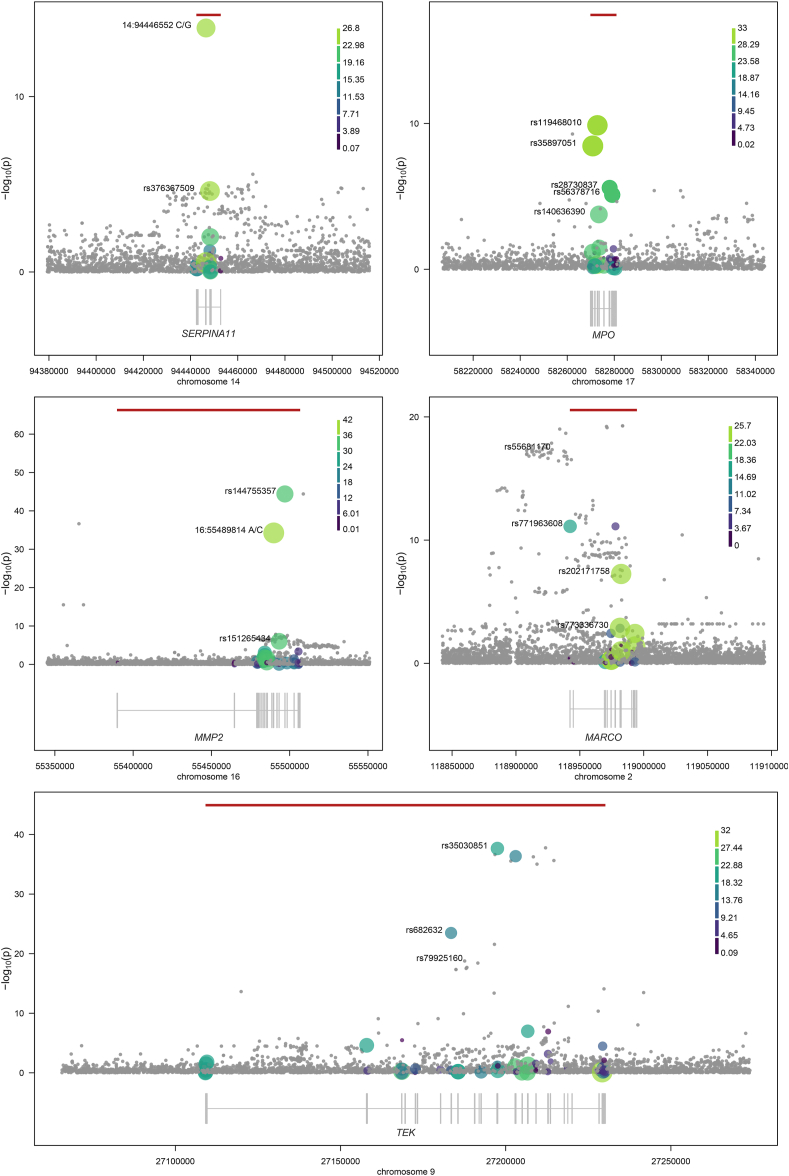
**Regional plots of the five burdens discovered in this study.** Circles denote the sequence variants identified in the region. Protein-coding genes are displayed in grey below the regional association plots, bars represent exons across all transcripts. Horizontal red lines indicate the −log10 of the burden signal p-value. Circles represent variants, with size and colour of circles proportional to the CADD score used for weighting. Grey circles indicate variants not included in the test. The plots have been generated using the plotburden software (https://github.com/hmgu-itg/plotburden).

After stringent quality control (see Methods), we identify 55 study-wide significant (p < 7.45 × 10^−11^) trait–gene pairs where at least two QV contribute to the association. We focus on 5 signals that do not overlap with a more strongly associated single-point association. We describe 4 *cis* signals for MARCO, TEK, MMP2 and MPO, and one *trans* association for GDF2 in the SERPINA11 gene (Figure 1). All detected associations are combinations of coding variants (exons +50 base pair margin) weighted by the CADD functional annotation score [47]. All except the *MPO* locus are detectable using a single-point meta-analysis, as one or more low-frequency QV pass the study-wide threshold on their own.

The MARCO, TEK and MMP2 *cis* signals have similar architectures and are discussed in detail in the Supplementary Text. The *trans* GDF2-*SERPINA11* signal may indicate novel SERPINA11-mediated inhibition of a GDF2-targeting protease, a mechanism previously documented for other members of these gene families (e.g. *GDF2* and *SERPINA1*, see Supplementary Text). We note that this signal is driven by two isolate-specific variants, the novel missense Pomak-exclusive chr14:94446552 G/C (p = 9.52 × 10^−14^, β = −1.17, σ = 0.152, MAF = 0.014) and the missense variant rs376367509 (p = 2.43 × 10^−5^, β = −1.31, σ = 0.311), which is monomorphic in all recorded populations according to Ensembl but observed at a MAF of 0.0059 in ORCADES. The MMP2-*cis* signal is similarly driven by associations exclusive to the Pomak cohort: the novel stop gained variant (chr14:55489814 C/A, p = 4.48 × 10^−30^, β = −2.59, σ = 0.21, MAF = 6.99 × 10^−3^), and the missense rs144755357 (p = 4.26 × 10^−38^, β = 2.18, σ = 0.15, MAF = 0.013), which has increased in frequency more than a thousand-fold compared to gnomAD exomes in non-Finnish Europeans. The signals in MARCO and TEK are similarly driven by variants that are either novel, or that exhibit strongly increased frequencies in one of the isolates analysed in this study.

We describe the *cis-*MPO signal in more detail below.

### 3.2 Description of the cis-MPO signal

Across all cohorts, this test includes 41 variants (25 in MANOLIS, 17 in Pomak, and 18 in ORCADES, Supplementary Table 2). Five variants contribute the bulk of the signal (single-point p < 0.05/41 = 0.001, in at least one cohort), however the magnitude of their contribution and the overall architecture of the burden varies across cohorts (Table 1). Only two QVs (rs28730837 and rs56378716) are present across all three cohorts, with the former nominally associated with MPO levels in Pomak and ORCADES only, and the latter in MANOLIS and Pomak only. Three out of two QVs display strong, significant changes in frequency in different isolates compared to cosmopolitan European populations (Supplementary Table 3). Interestingly, while rs119468010 and rs140636390 are enriched in ORCADES (3.8 fold) and MANOLIS (162-fold) respectively, rs28730837 exhibits significant depletion in all three isolates, up to 52-fold in MANOLIS. The QV with the strongest single-point association P-value across all cohorts is rs119468010, a missense variant present in ORCADES only (MAF = 0.014, β = −1.31, σ = 0.204, p = 1.34 × 10^−10^). Using this variant's genotypes as a covariate across the meta-analysis increases the meta-analysis burden P-value from 3.6 × 10^−18^ to 1.46 × 10^−12^, showing that it contributes only about a third of the rare variant signal at this locus. Similarly, no single variant, when conditioned upon, attenuates the P-value more than a third on the negative log scale for the meta-analysis, or by half in the single-cohort rare variant analysis. This underscores the genuine contribution of at least two variants per cohort to the meta-analysis burden signal, as well as a combined contribution by variants from all three cohorts.

**Table 1:** Cohort contribution and conditional P-values for the top five contributing variants.

	consequence for *MPO*	position on chr17	MANOLIS				Pomak				ORCADES				effect direction	meta-analysis		
			effect direction	single-point P	gene-based P	conditional P	effect direction	single-point P	gene-basedP	conditional P	effect direction	single-point P	gene-based P	conditional P		single-point P	gene-based P	conditional P
rs119468010	missense	58272835			6.79 × 10^−7^				1.39 × 10^−4^		–	1.34 × 10^−10^	2.78 × 10^−13^	1.64 × 10^−5^	–	1.34 × 10-10	3.60 × 10^−18^	1.46 × 10^−12^
rs28730837	missense	58278036	+	0.553		5.56 × 10^−7^	–	0.0157		6.62 × 10^−4^	–	1.96 × 10^−5^		6.55 × 10^−11^	–	2.60 × 10^−6^		8.30 × 10^−15^
rs56378716	missense	58279141	–	8.96 × 10^−3^		1.49 × 10^−5^	–	1.05 × 10^−3^		0.0181	–	0.117204		5.75 × 10^−13^	–	7.79 × 10^−6^		6.79 × 10^−14^
rs35897051	splice acceptor	58270865	–	6.26 × 10^−7^		2.26 × 10^−3^					–	1.26 × 10^−3^		1.00 × 10^−12^	–	3.48 × 10^−9^		2.89 × 10^−13^
rs140636390	missense	58273455	–	1.94 × 10^−4^		4.13 × 10^−5^									–	1.74 × 10^−4^		7.39 × 10^−16^

For each cohort, the single-point column contains the single-point P-value (Individual cohorts: Wald Test, Meta-analysis: Effect-size based) for that individual variant. The gene-based P contains the overall gene-based P-value for all QVs in that cohort, including the five displayed in the table. The conditional column is the gene-based P-value when the burden is conditioned on the genotypes of the variant of interest.

### 3.3 Signal architecture

The *ρ* parameter of the SKAT-O optimal test [37] performed in this study can inform about the genetic architecture of the signal (see Methods). While *ρ* = 1 corresponds to a pure burden (collapsing) test where all variants have concordant directions of effect, *ρ* = 0 corresponds to a pure SKAT (kernel-based) test. For the MPO signal, the optimal *ρ* values vary depending on the cohort and analysis, but are closer to a SKAT test for individual cohorts (0.3, 0.4 and 0.1 for MANOLIS, Pomak and ORCADES, respectively). In the meta-analysis *ρ* = 1, which indicates that the collapsing test is optimal. This is expected if the true signal is a burden, but the variants composing it are not all found in the same cohort. Accordingly, the five variants driving the signal, when nominally associated in any study, have their minor allele associated with a negative direction of effect on MPO levels, which confirms this signal as an MPO-decreasing burden of rare variants.

### 3.4 Independence from previous signals

The protein encoded by *MPO* is myeloperoxidase, a heme-containing peroxidase expressed mainly in neutrophils, where it catalyses the formation of reactive species instrumental in microbial killing. Because of MPO's role in processes thought to be crucial for CAD, including inflammation and tissue damage, MPO variants have been studied extensively both for their role in CAD and as part of GWAS of circulating MPO levels. Three of the 5 variants driving our burden are detectable at strong significance levels in the largest MPO single-point meta-analysis to date by Folkersen et al. [9] (rs28730837, MAF = 0.0255, β = −0.5407, σ = 0.0439, p = 7.98 × 10^−35^; rs56378716, MAF = 0.0209, β = −0.4947, σ = 0.0400, p = 4.15 × 10^−35^; rs35897051 MAF = 0.0066, β = −1.3122, σ = 0.0832, p = 5.54 × 10^−56^). Two are also present in an earlier study of the plasma proteome by Sun et al. [7] (rs28730837 p = 1.74 × 10^−11^, rs56378716 p = 1.10 × 10^−5^, rs35897051 p = 7.94 × 10^−23^). These variants are present in the summary statistics but are not reported as main signals, having been considered conditionally dependent on the reported rs34097845 and rs11079341. The two variants not detected in either meta-analysis, rs119468010 and rs140636390, are present only at low or very low frequencies in publicly available datasets (rs140636390 gnomAD allele count (AC) = 10, rs119468010 gnomAD AC = 470 AF = 0.001). When conditioned on the three variants previously implicated in GWAS studies (rs28730837, rs56378716, rs35897051), the p-value of the burden increases to 5.98 × 10^−7^, showing a distinct additional contribution of rs119468010 and rs140636390, the two variants private to MANOLIS and ORCADES, respectively.

### 3.5 Functional impact of burden variants

**Figure 2:**
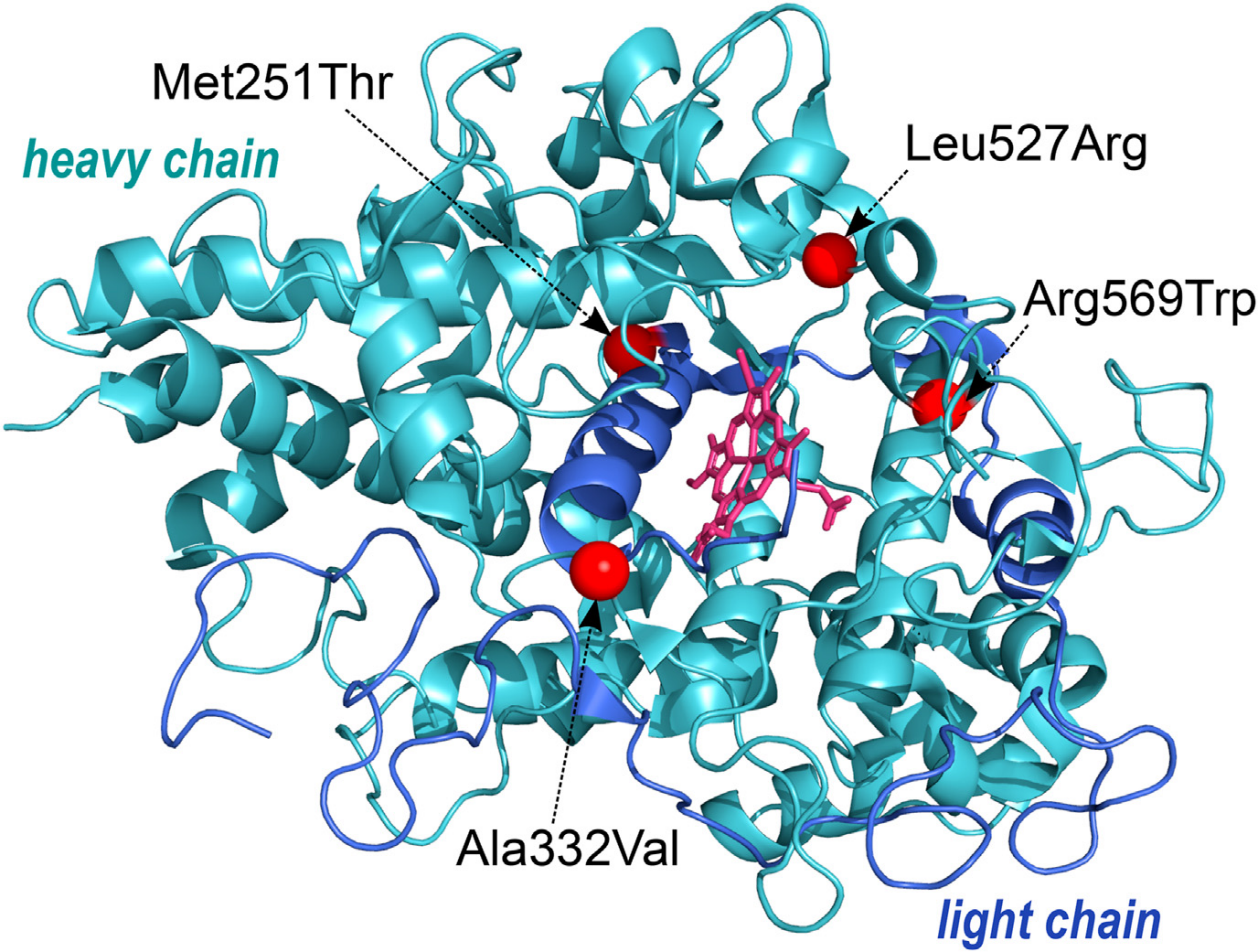
**3D Representation of the MPO protein with the four coding variants contributing to the signal represented in red** (rs56378716 (M251T), rs28730837 (A332V), rs119468010 (R569W), rs140636390 (L527R)).

Four of the five variants driving the signal (rs56378716 (M251T), rs28730837 (A332V), rs119468010 (R569W), rs35897051) are known pathogenic variants implicated in recessive MPO deficiency with clear loss-of-function mechanisms likely to reduce functional protein levels. Biochemical characterisation of rs56378716 and rs28730837 showed a complete loss of protein activity, whereas rs119468010 was shown to block heme insertion, and rs35897051 causing a large frameshifting insertion resulting in a non-functional protein. rs140636390 (L527R), the only variant without prior functional evidence, is likely to have a similar mechanism of action, as the affected leucine residue contacts the heme group crucial for protein function (Figure 2).

### 3.6 Association of MPO scores with cardiovascular outcomes

MPO has a long history as a candidate gene in cardiovascular disease (Supplementary Text). Protein genetic scores associated with disease outcomes can indicate either disease-induced or disease-causing dysregulation, and hint at a role for the protein in the disease process. We therefore sought to quantify the disease-predicting ability of recently implicated MPO-altering variants, including those described in this study, in the UK Biobank. We perform linear logistic regression of stroke and coronary artery disease (CAD) using MPO genetic scores (see Methods) and clinical predictors. We find clinical predictors such as sex, age, hypertension, hyperlipidemia, poor glycaemic control, obesity and smoking to be associated with almost all disease phenotypes (Supplementary Table 4) at nominal significance (P < 0.05). Clinical predictors have the highest p-values for the phenotypes with smallest case number (Supplementary Table 5), likely indicative of insufficient power for accurate disease risk prediction in these cases. We extend previous results from a 2017 study [43] (see Methods) which found that an MPO-increasing polygenic score based on a 2013 MPO GWAS meta-analysis was correlated with elevated risk of both primary and recurrent intracerebral haemorrhage. We perform ten additional regression analyses using increasingly dense MPO genetic scores (Supplementary Table 4, Supplementary Table 6). We confirm that the historical score is predictive of ischaemic (hospital-diagnosed (HD) F-test p = 0.010, self-reported (SR) and HD p = 7.73 × 10^−3^) and all (HD p = 1.83 × 10^−3^, SR + HD p = 4.26 × 10^−3^) stroke events, as well as all subtypes of CAD. The stratified serum and plasma versions of the score are weakly associated only in analyses with the largest CAD case numbers, indicating likely loss of power (e.g. all CAD SR + HD plasma p = 3.05 × 10^−3^, serum p = 0.0387) but hinting at a contribution of both the plasma and serum components. The score remains significant for the same phenotypes when a collapsed genotype for rare MPO variants is included as a predictor, although the rare variant component itself is not associated with any disease response. When we add variants significantly associated in three large, recent studies of MPO levels [[7], [8], [9]] to the score, the genetic MPO component only remained nominally predictive for chronic and incident CAD (SR + HD p = 0.0212, HD p = 0.0467). Any association of a genetic MPO predictor with stroke and CAD phenotypes is fully attenuated when we further add in variants significantly associated in the present meta-analysis.

## 4 Discussion

### 4.1 MPO associations in serum and plasma

In our study, protein levels were measured in serum for HELIC, and in plasma for ORCADES. Given MPO's main function in leukocytes, levels of the protein will be influenced by the leukocyte activation [48] and degranulation observed in complement fixation, which happens during the blood coagulation involved in serum generation from plasma. Genetic association signals from plasma and serum MPO have therefore been historically distinct [44], with plasma signals characterised by hits in the *MPO* and *GALNT2* regions, whereas the serum analyses are dominated by a strong *CFH* signal, with absence of a *cis-MPO* signal, as well as multiple *trans* signals. *CFH* encodes complement factor H, a key actor in the alternative complement pathway. The interplay and cross-talk between the complement and coagulation pathways is well-documented [49].

We broadly replicate previous findings, with a HELIC serum meta-analysis dominated by a *CFH* hit and the absence of a *cis*-*MPO* signal*,* whereas the meta-analysis including ORCADES is typical of a plasma analysis with a strong common-variant *MPO* signal (Supplementary Figure 2, Supplementary Text). Low-frequency MPO coding variants contribute markedly to the gene-based signal in the serum-measured HELIC cohorts, without a common variant signal. Together, these findings indicate that a *cis-*MPO signal arises from both a common genetic component mainly observable in plasma, and a rare variant component described in Table 1 and observable both in serum and plasma.

In general, we find that meta-analysing serum and plasma protein levels does not result in strong power loss for pQTL discovery. Out of 873 and 979 common and low-frequency raw signals identified in MANOLIS where tagging (LD > 0.8) variants could be found in Pomak (serum) and ORCADES (plasma), respectively, 142 (13.7%) and 109 (10.0%) replicate with the same direction of effect at a Bonferroni-corrected threshold (two-sided chi-squared two-sample test p = 0.0101). Given that high correlation is likely present at neighbouring signals, this threshold is conservative, and the slight observed increase in replication in Pomak subsides when nominal significance is used for replication (17.4% and 15.7%, p = 0.299). Combined with increased genetic proximity between the two Hellenic isolates, these results support the hypothesis that replication rates are similar between plasma and serum studies, except for a small number of pQTL reflecting interactions activated in coagulation pathways.

### 4.2 Are genetic scores of MPO predictive for stroke and CAD?

In our disease prediction model, we replicate a previous association between an MPO genetic score and risk of both stroke and CAD. However, both associations are attenuated when the score is updated with SNVs from recent, large MPO GWAS [[7], [8], [9]], as well as those associated in this study. We similarly find no significant predictive effect when using a stratified score that separates effects from the gene-based signal described in this work from common score variants.

Our finding that early MPO genetic scores are predictive of CAD and stroke risk must be tempered by the complex genetic architecture of myeloperoxidase levels. In particular, 6 out of 15 variants originally included in the 2013 score were located in the *CFH* and *HLA-C2* regions, both of which have been associated with cardiovascular disease risk and are involved in multiple biological pathways. When these variants are excluded, the association of any MPO score with either CAD or stroke disappears, even though the number of cases in the UK Biobank (maximum n = 17,148 for self-reported or hospital-diagnosed stroke) is the same order of magnitude as the 2017 study (n = 12,577 for the NINDS-SiGN subcohort). This suggests previous associations between MPO genetic scores and cardiovascular disease may have been driven by variants in these highly pleiotropic regions. Our results highlight that genetic scores require careful interpretation when inferring the role of intermediate traits such as proteomics in complex disease. More research will be required to unambiguously pinpoint myeloperoxidase as a therapeutic target in stroke and coronary artery disease.

## 5 Conclusions

In this work, we perform the first discovery meta-analysis of rare variant associations across three cohorts with whole genome sequencing. This proof-of-concept using deep proteomic data identifies associations of rare variants not detectable by single-point analysis, exemplified by a *cis* exonic MPO signal. We show an excess of association signal compared to previous studies, ascribed to two *MPO* variants, each exclusive to a specific study. This highlights the added value of this approach, which allows the inclusion of variants that only occur in certain populations. Our study details a reproducible pipeline for the single-cohort and cross-cohort analysis of functionally-weighted coding and regulatory variants. We describe robust quality control (QC) procedures based on conditional analysis that, unlike leave-one-out methods, account for linkage disequilibrium between variants and identify true rare variant signals. Finally, we note that the software used in this study does not allow single-cohort analysis of more than 30,000 individuals. As large whole exome and whole genome sequencing projects are gathering momentum [50,51], biobank-scale gene-based testing methods require further development [39,52,53]. Urgent methodological work is needed to scale up these methods to enable the next step in rare variant and complex trait research.
