# Supplementary material for: Gene-based whole genome sequencing meta-analysis of 250 circulating proteins in three isolated European populations
Source: Mol Metab. 2022 Apr 30;61:101509. doi: 10.1016/j.molmet.2022.101509 (PMC9118462; doi:10.1016/j.molmet.2022.101509)
Supplement: Multimedia component 1 [file mmc1.docx]

# Supplementary Text

## Detail on additional signals in *SERPINA11, MARCO, TEK* and *MMP2*

### GDF2-SERPINA11 trans signal

This association has not been described before, and is detectable using single-point data. We identify a driven by the ORCADES-specific missense rs376367509 (p=2.43x10^-5^, β=-1.31, σ=0.311) and the Pomak-specific novel missense chr14:94446552 G/C (p=9.52x10^-14^, β=-1.17, σ=0.152). Both are predicted to be deleterious by SIFT and probably damaging by PolyPhen. The signal is partially attenuated when conditioned on chr14:94446552 G/C (p_burden|chr14:94446552_G/C_ =5.64x10^-4^), and fully attenuated when rs376367509 is also conditioned on (p_burden|chr14:94446552_G/C,rs376367509_ =0.030), indicating a two-variant signal. MANOLIS does not contribute to this signal, with a rare-variant p-value of 0.14. GDF2, also called BMP9, is involved in ALK1-mediated cell signalling and plays a central role in angiogenesis and the maintenance of vascular quiescence[1]. SERPINs, the serine protease inhibitors, are a central control mechanisms of the 180 serine proteases active in humans. They are often capable of inhibiting multiple enzymes, such as antithrombin (SERPINC1), which targets multiple enzymes all involved in coagulation[2]. Consequently, disruptions in SERPIN activity lead to increased and sometimes pathological protease activity. The targets of SERPINA11 are unknown, however GDF2 proteolysis is inhibited by the related SERPINA1 [3]. The negative directions of effect of this association for likely damaging missense variants are concordant with SERPINA11 inhibiting a protease that targets GDF2.

### MARCO cis signal

We previously described [4] a single-point *cis* MARCO signal in MANOLIS with independent contributions by rs184649094 and rs55681170. We observe a cis-burden of rare variants (p=2.53x10^-22^, ρ=1) contributed to by the MANOLIS-exclusive missense variant rs771963608, which is in strong LD with rs184649094 (r^2^=0.94, d’=1). However, the burden is only partially attenuated when conditioned on the two known variants (p_burden|rs184649094, rs55681170_=2.05x10^-8^). Other contributors include the Pomak-exclusive missense variant rs202171758 (p=8.97x10^-8^, β=-1.36, σ=0.251) and to a lesser extent, the ORCADES-exclusive splice donor rs773336730 (p=1.34x10^-3^, β=-1.57, σ=0.490). This signal exemplifies the value of rare variant aggregation tests at elucidating and complementing existing signals. It pinpoints rs771963608 rather than rs184649094, located 314kb away, as a contributor, and highlights the role of several further low-frequency variants that fail to reach study-wide significance. rs771963608’s frequency has increased in MANOLIS (MAF=0.012) by up to 1,500 fold compared to cosmopolitan populations (gnomADe MAF=6.38x10^-5^ gnomADg MAF=3.49x10^-5^, TOPMed MAF=7.96x10^-6^). Similarly, the T (effect) allele of rs202171758 has increased in frequency in Pomak (MAF=6x10^-3^) compared to non-Finnish populations by at least 16-fold (gnomADe NFE MAF=1.07x10^-4^ gnomADg NFE MAF=2.37x10^-4^, TOPMed AC=0), and the splice donor rs773336730 similarly raised from frequencies of 2.69x10^-5^ and 7.96x10^-6^ in gnomAD and TOPMed, respectively, to 2.68x10^-3^ in ORCADES (a 100 and over 300-fold increase).

### TEK *cis* signal

A *cis* signal of 66 coding rare variants was detected for the angiopoietin-1 receptor (TIE2) encoded by the *TEK* gene (p=1.19x10^-45^, ρ=0.1). The signal emerged from the CADD-weighted analysis. The main contributing cohorts are MANOLIS and Pomak cohorts (p=1.48x10^-10^, ρ=0.1 and p=4.04x10^-34^, ρ=0.4, respectively), with a minor contribution from ORCADES (p=3.05x10^-6^, ρ=0). The signal is contributed to by two LD-blocs led by rs35030851 (p=2.21x10^-38^, β=0.793, σ=0.187) and rs682632 (p=3.36x10^-24^, β=0.745, σ=0.0735), two known TEK-increasing variants. When conditioned on these two variants, the burden attenuates to p=0.023. A block of intronic variants led by rs79925160 (p=1.69x10^-19^, β=-0.482, σ=0.0534) is present in the single-point but no variants from it are included in the aggregation test. Conditional analysis for this variant decreases the signal to p=8.90x10^-37^, reflecting partial LD with rs35030851 (r^2^_rs35030851,rs79925160_=0.53, d’_rs35030851, rs79925160_=0.86). Despite both leading variants having the same effect direction, the model leaned favoured a kernel test (p_BT_=1.88x10^-38^, p_SKAT_=4.73x10^-45^). We hypothesise that this is due to the inclusion of many other variants with less significant effect sizes.

### MMP2 *cis* signal

An exonic cis-MMP2 signal was driven by two Pomak-exclusive variants, the missense rs144755357, (p=4.26x10^-38^, β=2.18, σ=0.155, MAF=0.013), and the novel stop gained chr14:55489814 C/A (p=4.48x10^-30^, β=-2.59, σ=0.21, MAF=6.99x10^-3^). ORCADES adds a minor contribution with the missense variant rs151265434 (p=9.62x10^-7^, β=-1.90, σ=0.38, MAF=3.68x10^-3^). Similar to MPO, MMP2’s (Matrix Metallopeptidase 2) role in disease has been investigated in multiple studies. rs243865 and rs2285053 [5; 6] were identified early as modifying plasma levels of MMP2. These polymorphisms were associated in candidate gene studies with a number of complex traits, notably end-stage kidney disease[7], heart failure prognosis[8], acne[9], osteolysis[10], bone mineral density[11], and cancer processes[12], among others. Twenty variants have been previously identified at genome-wide significance in studies of circulating MMP2 [13; 14] (rs1816594, rs2110820, rs7187754, rs9302670, rs6499761, rs8055282, rs2540730, rs11076098, rs837531, rs837535, rs837537, rs837539, rs708267, rs708268, rs1561219, rs1561220, rs1347653, rs243837, rs572379748, rs16955280). The rare variant signal described here, however, is independent, with a conditional P-value of 4.45x10^-62^ and 7.95x10^-6^ in Pomak and ORCADES, respectively. This makes the MMP7-*cis* signal described here novel and likely private to Pomak, since rs144755357 has increased in frequency more than a hundred-fold in Pomak compared to TOPMed and gnomAD exomes and genomes.

## Serum and Plasma signals

Three of the serum signals previously reported in Europeans replicate in our HELIC serum meta-analysis (rs800292 p=2.08x10^-15^, rs6680396 p=1.20x10^-15^, rs35897051 p=4.31x10^-7^), whereas none of the plasma signals do. One variant reported in an African American analysis[15], rs505102, also replicates at p=6.91x10^-15^. These signals are attenuated in the HELIC/ORCADES plasma/serum meta-analysis, except for rs35897051 (p_meta_=3.48x10^-9^). rs34097845, the only variant reported in the Sun et al. plasma meta-analysis and strongest association in Folkersen et al., is the only one to replicate (p= 0.002886) with concordant effect direction in the HELIC serum meta-analysis. Interestingly, this is also the case for ORCADES (p= 0.00100179), despite a higher expected replication rate for plasma levels.

## MPO mechanisms in atherosclerosis

Three broad pathways have been proposed through which MPO could increase CVD risk[16].

### Oxidative action on lipoproteins

First, MPO-derived oxidants target LDL, forming atherogenic species more easily recognisable by macrophage scavenger receptors, leading to the formation of foam cells within the lipid core of atherosclerotic plaques. MPO also modifies HDL and the HDL-associated PON1 (Paraoxonase 1), leading to reduced cholesterol efflux from macrophages back to the liver. Because of its oxidative action on both LDL and HDL, as well as its interaction with PON1, lipid-adjusted measures of MPO are sometimes studied, and have successfully been associated with other risk factors for CAD, as well as risk of CVD events[17].

### Promoter of endothelial dysfunction

MPO is able to cause endothelial dysfunction by disrupting vasomotor tone, fibrinolysis and coagulation pathways, modulation of inflammation, angiogenesis, vessel repair and remodelling[16].

It is thought to interfere with NO metabolism, a crucial pathway in normal endothelial function, by both using it as a substrate and impairing the function of NO-synthase (NOS).

### Weakening of atherosclerotic plaques

MPO weakens the fibrous protective layer that covers the foam cell core of CAD lesions by multiple mechanisms. It promotes apoptosis of endothelial cells, activates matrix metalloproteinases (MMP) that degrade the extracellular matrix of the fibrous cap, silences MMP inhibitors, and is also able to bind to the matrix itself and degrade it.

## MPO Inhibitors for the treatment of CAD

Due to its history, MPO has been targeted by multiple molecules to improve CAD patient care, so far with mixed results. Benzoic acid hydrazides act as reversible competitive inhibitors, and are not able to function optimally in in vivo conditions. A ferulic acid derivative improved cholesterol transport, endothelial function, and reduced plaque size and MPO activity, but its effect on cholesterol oxidation remains uncertain. Several thiouracil and 2-thioxanthine derivatives have also been studied as irreversible MPO inhibitors, in both human and mice models, with some promise. Finally, commonly used substances such as acetaminophen and dietary flavonoids have also been proposed as MPO inhibitors, to some controversy.

Several triazolopyrimidines are the latest molecules to have been added to the list of reversible myeloperoxidase inhibitors [18].

## Myeloperoxidase as a CVD target

*MPO* encodes Myeloperoxidase, a heme-containing lysosomal peroxidase enzyme which can both produce various free radical and reactive oxidants, and uniquely catalyses the formation of hypohalous acids from hydrogen peroxide and halides. It is chiefly stored within the azurophilic granules of circulating neutrophils, and to a lesser extent in monocytes and tissue macrophages (19622015), and is released upon activation. This enzymatic activity has implicated MPO in multiple immune- and inflammation-associated disorders, such as coronary artery disease (CAD), rheumatoid arthritis, systemic lupus erythematosus, multiple sclerosis, and inflammatory diseases of the lung and kidneys.

MPO deficiency (MPOD) is the most common neutrophil disorder (1:2000) but can go undiagnosed if no other impairment of host defence, such as diabetes mellitus, is present. This is because MPOD patients usually do not show signs of chronic infections [19; 20]. This is thought to be due to MPO-independent pathways producing many of the same antimicrobial species, and a potential compensation by adaptive immunity.

Due to these inflammation-promoting and oxidative properties, MPO was an early candidate biomarker for cardiovascular diseases. Early studies showed that incidence of CAD was lower in MPOD patients than the general population [20]. Further observational studies have found associations between circulating MPO levels and risk of CAD in both healthy and at-risk individuals [21-23]. Results for stroke and small vessel diseases are more ambiguous and failed to demonstrate a consistent association between MPO levels and risk [16]. Enzymatically active MPO is found in atherosclerotic plaque [24], particularly in late-stage vulnerable or ruptured plaques as opposed to early-stage fatty streaks[25]. As a consequence, recent methods have proposed using MPO as a biomarker of vulnerable atherosclerotic plaque [26]. Myeloperoxidase has also been shown to play a critical role in tissue remodelling after a myocardial infarction [27; 28]. It is thought that MPO modulates CVD risk[16] through several different pathways[16], and several MPO inhibitors have been developed as experimental CAD treatments[18].

## Detailed disease risk modelling results

Outputs of logistic regressions from the R software are found below.

### Reiner et al. score and MPO burden

stroke.srhd.subarachnoid

=========================

Call:

glm(formula = rf, family = binomial(link = "logit"), data = ms,

maxit = 100)

Deviance Residuals:

Min 1Q Median 3Q Max

-0.1789 -0.0943 -0.0815 -0.0707 3.8009

Coefficients:

Estimate Std. Error z value Pr(>|z|)

(Intercept) -6.7736252 0.1890116 -35.837 < 2e-16 ***

old.nompo 0.0358767 0.0253529 1.415 0.157041

rvMPOrec -0.0400171 0.0964449 -0.415 0.678199

PC1 -0.0002786 0.0005413 -0.515 0.606789

PC2 0.0007687 0.0010905 0.705 0.480862

PC3 -0.0001860 0.0020302 -0.092 0.927003

PC4 0.0098745 0.0029920 3.300 0.000966 ***

PC5 0.0059296 0.0031635 1.874 0.060876 .

PC6 -0.0134666 0.0089083 -1.512 0.130611

PC7 -0.0129556 0.0063363 -2.045 0.040889 *

PC8 -0.0044405 0.0069945 -0.635 0.525524

PC9 0.0006585 0.0055486 0.119 0.905528

PC10 0.0008348 0.0070484 0.118 0.905715

sexMale -0.4437055 0.0536512 -8.270 < 2e-16 ***

age 0.0157247 0.0032334 4.863 1.15e-06 ***

hypertensionTRUE 0.3677567 0.0542698 6.776 1.23e-11 ***

hyperlipidemiaTRUE -0.0455201 0.0539627 -0.844 0.398923

gly_controlTRUE -0.4435373 0.3040718 -1.459 0.144659

obesityTRUE -0.0793286 0.0593831 -1.336 0.181589

smoking_status 0.4097896 0.0347025 11.809 < 2e-16 ***

---

Signif. codes: 0 ‘***’ 0.001 ‘**’ 0.01 ‘*’ 0.05 ‘.’ 0.1 ‘ ’ 1

(Dispersion parameter for binomial family taken to be 1)

Null deviance: 21381 on 443107 degrees of freedom

Residual deviance: 21089 on 443088 degrees of freedom

(45112 observations deleted due to missingness)

AIC: 21129

Number of Fisher Scoring iterations: 9

stroke.hd.subarachnoid

=========================

Call:

glm(formula = rf, family = binomial(link = "logit"), data = ms,

maxit = 100)

Deviance Residuals:

Min 1Q Median 3Q Max

-0.1781 -0.0872 -0.0754 -0.0649 3.8112

Coefficients:

Estimate Std. Error z value Pr(>|z|)

(Intercept) -6.789e+00 2.027e-01 -33.498 < 2e-16 ***

old.nompo 2.849e-02 2.730e-02 1.044 0.29667

rvMPOrec -2.145e-02 1.033e-01 -0.208 0.83557

PC1 5.491e-05 5.568e-04 0.099 0.92145

PC2 5.874e-04 1.115e-03 0.527 0.59847

PC3 -5.153e-04 2.098e-03 -0.246 0.80602

PC4 9.310e-03 3.157e-03 2.949 0.00319 **

PC5 8.648e-03 3.361e-03 2.573 0.01008 *

PC6 -1.324e-02 9.561e-03 -1.385 0.16600

PC7 -1.154e-02 6.648e-03 -1.736 0.08261 .

PC8 -1.434e-03 7.451e-03 -0.192 0.84736

PC9 8.566e-03 6.216e-03 1.378 0.16818

PC10 3.058e-04 7.517e-03 0.041 0.96755

sexMale -5.024e-01 5.819e-02 -8.633 < 2e-16 ***

age 1.355e-02 3.475e-03 3.899 9.66e-05 ***

hypertensionTRUE 3.647e-01 5.839e-02 6.245 4.23e-10 ***

hyperlipidemiaTRUE -3.922e-02 5.827e-02 -0.673 0.50083

gly_controlTRUE -3.681e-01 3.190e-01 -1.154 0.24861

obesityTRUE -1.169e-01 6.463e-02 -1.809 0.07048 .

smoking_status 4.378e-01 3.716e-02 11.781 < 2e-16 ***

---

Signif. codes: 0 ‘***’ 0.001 ‘**’ 0.01 ‘*’ 0.05 ‘.’ 0.1 ‘ ’ 1

(Dispersion parameter for binomial family taken to be 1)

Null deviance: 18812 on 443107 degrees of freedom

Residual deviance: 18535 on 443088 degrees of freedom

(45112 observations deleted due to missingness)

AIC: 18575

Number of Fisher Scoring iterations: 9

stroke.srhd.intracerebral

=========================

Call:

glm(formula = rf, family = binomial(link = "logit"), data = ms,

maxit = 100)

Deviance Residuals:

Min 1Q Median 3Q Max

-0.2559 -0.1091 -0.0865 -0.0674 3.8206

Coefficients:

Estimate Std. Error z value Pr(>|z|)

(Intercept) -9.2372858 0.1926175 -47.957 < 2e-16 ***

old.nompo 0.0136310 0.0230133 0.592 0.553643

rvMPOrec -0.0510587 0.0885149 -0.577 0.564050

PC1 0.0006825 0.0004847 1.408 0.159135

PC2 -0.0013164 0.0008244 -1.597 0.110321

PC3 -0.0003534 0.0015302 -0.231 0.817358

PC4 0.0053271 0.0024700 2.157 0.031026 *

PC5 0.0068412 0.0029249 2.339 0.019337 *

PC6 -0.0057440 0.0060020 -0.957 0.338563

PC7 0.0029802 0.0053734 0.555 0.579157

PC8 -0.0029878 0.0057419 -0.520 0.602822

PC9 0.0026867 0.0051027 0.527 0.598529

PC10 -0.0044008 0.0057795 -0.761 0.446392

sexMale 0.2575656 0.0481604 5.348 8.89e-08 ***

age 0.0560317 0.0031563 17.752 < 2e-16 ***

hypertensionTRUE 0.5014226 0.0518087 9.678 < 2e-16 ***

hyperlipidemiaTRUE -0.0853770 0.0481448 -1.773 0.076173 .

gly_controlTRUE 0.4248175 0.1706463 2.489 0.012794 *

obesityTRUE -0.0246147 0.0531310 -0.463 0.643162

smoking_status 0.1291554 0.0340071 3.798 0.000146 ***

---

Signif. codes: 0 ‘***’ 0.001 ‘**’ 0.01 ‘*’ 0.05 ‘.’ 0.1 ‘ ’ 1

(Dispersion parameter for binomial family taken to be 1)

Null deviance: 25042 on 443107 degrees of freedom

Residual deviance: 24359 on 443088 degrees of freedom

(45112 observations deleted due to missingness)

AIC: 24399

Number of Fisher Scoring iterations: 8

stroke.hd.intracerebral

=========================

Call:

glm(formula = rf, family = binomial(link = "logit"), data = ms,

maxit = 100)

Deviance Residuals:

Min 1Q Median 3Q Max

-0.2596 -0.1048 -0.0816 -0.0627 3.8160

Coefficients:

Estimate Std. Error z value Pr(>|z|)

(Intercept) -9.4867997 0.2033594 -46.650 < 2e-16 ***

old.nompo 0.0101875 0.0240759 0.423 0.67219

rvMPOrec -0.0868762 0.0939594 -0.925 0.35517

PC1 0.0006064 0.0005153 1.177 0.23929

PC2 -0.0012026 0.0008686 -1.385 0.16617

PC3 -0.0009600 0.0016260 -0.590 0.55494

PC4 0.0055470 0.0026415 2.100 0.03573 *

PC5 0.0065134 0.0030728 2.120 0.03403 *

PC6 -0.0068411 0.0066634 -1.027 0.30457

PC7 0.0053509 0.0056473 0.948 0.34338

PC8 -0.0020071 0.0061804 -0.325 0.74537

PC9 0.0014408 0.0053208 0.271 0.78655

PC10 -0.0083443 0.0061029 -1.367 0.17154

sexMale 0.2712938 0.0504735 5.375 7.66e-08 ***

age 0.0578456 0.0033233 17.406 < 2e-16 ***

hypertensionTRUE 0.5698518 0.0548586 10.388 < 2e-16 ***

hyperlipidemiaTRUE -0.1004638 0.0503659 -1.995 0.04608 *

gly_controlTRUE 0.4638654 0.1756858 2.640 0.00828 **

obesityTRUE -0.0570134 0.0559129 -1.020 0.30788

smoking_status 0.1393338 0.0355730 3.917 8.97e-05 ***

---

Signif. codes: 0 ‘***’ 0.001 ‘**’ 0.01 ‘*’ 0.05 ‘.’ 0.1 ‘ ’ 1

(Dispersion parameter for binomial family taken to be 1)

Null deviance: 23183 on 443107 degrees of freedom

Residual deviance: 22487 on 443088 degrees of freedom

(45112 observations deleted due to missingness)

AIC: 22527

Number of Fisher Scoring iterations: 9

stroke.srhd.ischaemic

=========================

Call:

glm(formula = rf, family = binomial(link = "logit"), data = ms,

maxit = 100)

Deviance Residuals:

Min 1Q Median 3Q Max

-0.8436 -0.2284 -0.1718 -0.1259 3.5236

Coefficients:

Estimate Std. Error z value Pr(>|z|)

(Intercept) -8.6289383 0.0950747 -90.760 < 2e-16 ***

old.nompo 0.0289445 0.0109752 2.637 0.00836 **

rvMPOrec 0.0031440 0.0411841 0.076 0.93915

PC1 0.0008200 0.0002205 3.719 0.00020 ***

PC2 0.0010747 0.0005219 2.059 0.03948 *

PC3 0.0013606 0.0009288 1.465 0.14296

PC4 0.0074200 0.0012277 6.044 1.50e-09 ***

PC5 0.0066351 0.0013826 4.799 1.59e-06 ***

PC6 -0.0113850 0.0043406 -2.623 0.00872 **

PC7 -0.0027979 0.0025698 -1.089 0.27627

PC8 0.0068709 0.0031793 2.161 0.03068 *

PC9 0.0145540 0.0025565 5.693 1.25e-08 ***

PC10 0.0023908 0.0028604 0.836 0.40325

sexMale 0.4284189 0.0235025 18.229 < 2e-16 ***

age 0.0640292 0.0015361 41.684 < 2e-16 ***

hypertensionTRUE 0.4983259 0.0252011 19.774 < 2e-16 ***

hyperlipidemiaTRUE 0.1491623 0.0226554 6.584 4.58e-11 ***

gly_controlTRUE 1.0274742 0.0598647 17.163 < 2e-16 ***

obesityTRUE 0.2920261 0.0236018 12.373 < 2e-16 ***

smoking_status 0.2953121 0.0158365 18.648 < 2e-16 ***

---

Signif. codes: 0 ‘***’ 0.001 ‘**’ 0.01 ‘*’ 0.05 ‘.’ 0.1 ‘ ’ 1

(Dispersion parameter for binomial family taken to be 1)

Null deviance: 86906 on 443107 degrees of freedom

Residual deviance: 81709 on 443088 degrees of freedom

(45112 observations deleted due to missingness)

AIC: 81749

Number of Fisher Scoring iterations: 7

stroke.hd.ischaemic

=========================

Call:

glm(formula = rf, family = binomial(link = "logit"), data = ms,

maxit = 100)

Deviance Residuals:

Min 1Q Median 3Q Max

-0.8410 -0.2282 -0.1718 -0.1260 3.5212

Coefficients:

Estimate Std. Error z value Pr(>|z|)

(Intercept) -8.6169102 0.0951116 -90.598 < 2e-16 ***

old.nompo 0.0300544 0.0109886 2.735 0.006237 **

rvMPOrec 0.0058264 0.0411865 0.141 0.887503

PC1 0.0008188 0.0002205 3.713 0.000205 ***

PC2 0.0010858 0.0005221 2.080 0.037552 *

PC3 0.0013267 0.0009293 1.428 0.153403

PC4 0.0073618 0.0012284 5.993 2.06e-09 ***

PC5 0.0065806 0.0013842 4.754 1.99e-06 ***

PC6 -0.0114054 0.0043535 -2.620 0.008797 **

PC7 -0.0028311 0.0025710 -1.101 0.270825

PC8 0.0070137 0.0031842 2.203 0.027621 *

PC9 0.0147046 0.0025607 5.742 9.34e-09 ***

PC10 0.0027468 0.0028657 0.958 0.337816

sexMale 0.4271438 0.0235216 18.160 < 2e-16 ***

age 0.0638572 0.0015370 41.547 < 2e-16 ***

hypertensionTRUE 0.4967043 0.0252143 19.699 < 2e-16 ***

hyperlipidemiaTRUE 0.1468585 0.0226803 6.475 9.47e-11 ***

gly_controlTRUE 1.0319656 0.0598649 17.238 < 2e-16 ***

obesityTRUE 0.2886197 0.0236403 12.209 < 2e-16 ***

smoking_status 0.2953976 0.0158505 18.636 < 2e-16 ***

---

Signif. codes: 0 ‘***’ 0.001 ‘**’ 0.01 ‘*’ 0.05 ‘.’ 0.1 ‘ ’ 1

(Dispersion parameter for binomial family taken to be 1)

Null deviance: 86750 on 443107 degrees of freedom

Residual deviance: 81590 on 443088 degrees of freedom

(45112 observations deleted due to missingness)

AIC: 81630

Number of Fisher Scoring iterations: 7

stroke.srhd.all

=========================

Call:

glm(formula = rf, family = binomial(link = "logit"), data = ms,

maxit = 100)

Deviance Residuals:

Min 1Q Median 3Q Max

-0.8760 -0.3033 -0.2354 -0.1765 3.2937

Coefficients:

Estimate Std. Error z value Pr(>|z|)

(Intercept) -7.5877188 0.0706230 -107.440 < 2e-16 ***

old.nompo 0.0264051 0.0083432 3.165 0.001552 **

rvMPOrec -0.0214908 0.0315975 -0.680 0.496413

PC1 0.0006599 0.0001710 3.858 0.000114 ***

PC2 0.0006134 0.0003772 1.626 0.103878

PC3 0.0011090 0.0006768 1.639 0.101273

PC4 0.0075768 0.0009255 8.186 2.70e-16 ***

PC5 0.0083370 0.0010461 7.969 1.60e-15 ***

PC6 -0.0064020 0.0026546 -2.412 0.015881 *

PC7 -0.0022695 0.0019464 -1.166 0.243610

PC8 0.0025625 0.0021809 1.175 0.239991

PC9 0.0092938 0.0018880 4.922 8.55e-07 ***

PC10 0.0031381 0.0021730 1.444 0.148699

sexMale 0.2908344 0.0175806 16.543 < 2e-16 ***

age 0.0589336 0.0011504 51.228 < 2e-16 ***

hypertensionTRUE 0.4177621 0.0186717 22.374 < 2e-16 ***

hyperlipidemiaTRUE 0.1549322 0.0172311 8.991 < 2e-16 ***

gly_controlTRUE 0.8633816 0.0506363 17.051 < 2e-16 ***

obesityTRUE 0.2842625 0.0180579 15.742 < 2e-16 ***

smoking_status 0.3129809 0.0119724 26.142 < 2e-16 ***

---

Signif. codes: 0 ‘***’ 0.001 ‘**’ 0.01 ‘*’ 0.05 ‘.’ 0.1 ‘ ’ 1

(Dispersion parameter for binomial family taken to be 1)

Null deviance: 135281 on 443107 degrees of freedom

Residual deviance: 128034 on 443088 degrees of freedom

(45112 observations deleted due to missingness)

AIC: 128074

Number of Fisher Scoring iterations: 6

stroke.hd.all

=========================

Call:

glm(formula = rf, family = binomial(link = "logit"), data = ms,

maxit = 100)

Deviance Residuals:

Min 1Q Median 3Q Max

-0.8193 -0.2590 -0.2008 -0.1517 3.3781

Coefficients:

Estimate Std. Error z value Pr(>|z|)

(Intercept) -7.8024981 0.0821779 -94.946 < 2e-16 ***

old.nompo 0.0284584 0.0097385 2.922 0.00348 **

rvMPOrec -0.0182162 0.0368593 -0.494 0.62116

PC1 0.0007979 0.0001939 4.114 3.88e-05 ***

PC2 0.0006107 0.0004269 1.430 0.15261

PC3 0.0007709 0.0007710 1.000 0.31733

PC4 0.0075867 0.0010882 6.972 3.12e-12 ***

PC5 0.0071688 0.0012246 5.854 4.80e-09 ***

PC6 -0.0114389 0.0037014 -3.090 0.00200 **

PC7 -0.0031453 0.0022813 -1.379 0.16798

PC8 0.0054825 0.0027636 1.984 0.04728 *

PC9 0.0126043 0.0022495 5.603 2.11e-08 ***

PC10 0.0007824 0.0025307 0.309 0.75720

sexMale 0.3081622 0.0205457 14.999 < 2e-16 ***

age 0.0570144 0.0013400 42.548 < 2e-16 ***

hypertensionTRUE 0.4851293 0.0220095 22.042 < 2e-16 ***

hyperlipidemiaTRUE 0.0971390 0.0201390 4.823 1.41e-06 ***

gly_controlTRUE 0.9243776 0.0570287 16.209 < 2e-16 ***

obesityTRUE 0.2088892 0.0212807 9.816 < 2e-16 ***

smoking_status 0.2928384 0.0139902 20.932 < 2e-16 ***

---

Signif. codes: 0 ‘***’ 0.001 ‘**’ 0.01 ‘*’ 0.05 ‘.’ 0.1 ‘ ’ 1

(Dispersion parameter for binomial family taken to be 1)

Null deviance: 105309 on 443107 degrees of freedom

Residual deviance: 100108 on 443088 degrees of freedom

(45112 observations deleted due to missingness)

AIC: 100148

Number of Fisher Scoring iterations: 7

cad.nochronic.withsr

=========================

Call:

glm(formula = rf, family = binomial(link = "logit"), data = ms,

maxit = 100)

Deviance Residuals:

Min 1Q Median 3Q Max

-1.5649 -0.4103 -0.2782 -0.1865 3.2983

Coefficients:

Estimate Std. Error z value Pr(>|z|)

(Intercept) -7.5755087 0.0522603 -144.957 < 2e-16 ***

old.nompo 0.0182671 0.0060994 2.995 0.002745 **

rvMPOrec -0.0089415 0.0231022 -0.387 0.698725

PC1 -0.0002156 0.0001581 -1.364 0.172621

PC2 -0.0023607 0.0002768 -8.527 < 2e-16 ***

PC3 0.0061790 0.0004633 13.337 < 2e-16 ***

PC4 0.0048514 0.0005959 8.141 3.92e-16 ***

PC5 0.0067602 0.0007764 8.707 < 2e-16 ***

PC6 -0.0018815 0.0016969 -1.109 0.267514

PC7 -0.0020846 0.0012875 -1.619 0.105426

PC8 0.0048563 0.0014379 3.377 0.000732 ***

PC9 0.0107561 0.0013904 7.736 1.03e-14 ***

PC10 0.0038664 0.0014099 2.742 0.006101 **

sexMale 0.9755306 0.0138986 70.189 < 2e-16 ***

age 0.0576062 0.0008398 68.597 < 2e-16 ***

hypertensionTRUE 0.4578324 0.0139607 32.794 < 2e-16 ***

hyperlipidemiaTRUE 0.7839635 0.0127481 61.496 < 2e-16 ***

gly_controlTRUE 0.9094946 0.0380016 23.933 < 2e-16 ***

obesityTRUE 0.3963531 0.0130681 30.330 < 2e-16 ***

smoking_status 0.3309489 0.0087730 37.724 < 2e-16 ***

---

Signif. codes: 0 ‘***’ 0.001 ‘**’ 0.01 ‘*’ 0.05 ‘.’ 0.1 ‘ ’ 1

(Dispersion parameter for binomial family taken to be 1)

Null deviance: 232155 on 443107 degrees of freedom

Residual deviance: 201661 on 443088 degrees of freedom

(45112 observations deleted due to missingness)

AIC: 201701

Number of Fisher Scoring iterations: 6

cad.nochronic.hd

=========================

Call:

glm(formula = rf, family = binomial(link = "logit"), data = ms,

maxit = 100)

Deviance Residuals:

Min 1Q Median 3Q Max

-1.5269 -0.3956 -0.2687 -0.1813 3.3283

Coefficients:

Estimate Std. Error z value Pr(>|z|)

(Intercept) -7.5681168 0.0539214 -140.355 < 2e-16 ***

old.nompo 0.0169085 0.0063064 2.681 0.00734 **

rvMPOrec 0.0005583 0.0238187 0.023 0.98130

PC1 -0.0004755 0.0001704 -2.791 0.00526 **

PC2 -0.0024739 0.0002919 -8.476 < 2e-16 ***

PC3 0.0064468 0.0004842 13.313 < 2e-16 ***

PC4 0.0052833 0.0006210 8.508 < 2e-16 ***

PC5 0.0068639 0.0008016 8.563 < 2e-16 ***

PC6 -0.0025668 0.0017956 -1.429 0.15287

PC7 -0.0021875 0.0013473 -1.624 0.10446

PC8 0.0041303 0.0015093 2.737 0.00621 **

PC9 0.0120209 0.0014445 8.322 < 2e-16 ***

PC10 0.0042564 0.0014553 2.925 0.00345 **

sexMale 0.9640939 0.0144212 66.853 < 2e-16 ***

age 0.0560506 0.0008674 64.621 < 2e-16 ***

hypertensionTRUE 0.4735825 0.0145070 32.645 < 2e-16 ***

hyperlipidemiaTRUE 0.7730098 0.0132112 58.512 < 2e-16 ***

gly_controlTRUE 0.9141857 0.0386914 23.628 < 2e-16 ***

obesityTRUE 0.3834135 0.0135097 28.381 < 2e-16 ***

smoking_status 0.3301633 0.0090685 36.408 < 2e-16 ***

---

Signif. codes: 0 ‘***’ 0.001 ‘**’ 0.01 ‘*’ 0.05 ‘.’ 0.1 ‘ ’ 1

(Dispersion parameter for binomial family taken to be 1)

Null deviance: 219311 on 443107 degrees of freedom

Residual deviance: 191285 on 443088 degrees of freedom

(45112 observations deleted due to missingness)

AIC: 191325

Number of Fisher Scoring iterations: 6

cad.chronic.withsr

=========================

Call:

glm(formula = rf, family = binomial(link = "logit"), data = ms,

maxit = 100)

Deviance Residuals:

Min 1Q Median 3Q Max

-1.7232 -0.4995 -0.3513 -0.2331 3.1216

Coefficients:

Estimate Std. Error z value Pr(>|z|)

(Intercept) -7.2917753 0.0443964 -164.243 < 2e-16 ***

old.nompo 0.0153450 0.0051769 2.964 0.00304 **

rvMPOrec -0.0038095 0.0195559 -0.195 0.84555

PC1 0.0002288 0.0001231 1.859 0.06304 .

PC2 -0.0021873 0.0002279 -9.599 < 2e-16 ***

PC3 0.0062776 0.0003881 16.175 < 2e-16 ***

PC4 0.0045362 0.0005045 8.991 < 2e-16 ***

PC5 0.0057150 0.0006630 8.620 < 2e-16 ***

PC6 -0.0015365 0.0013423 -1.145 0.25236

PC7 -0.0002419 0.0010943 -0.221 0.82507

PC8 0.0032468 0.0011962 2.714 0.00664 **

PC9 0.0096582 0.0011712 8.246 < 2e-16 ***

PC10 0.0062770 0.0012112 5.183 2.19e-07 ***

sexMale 0.8005405 0.0112265 71.308 < 2e-16 ***

age 0.0642719 0.0007150 89.895 < 2e-16 ***

hypertensionTRUE 0.4061830 0.0115300 35.228 < 2e-16 ***

hyperlipidemiaTRUE 0.5905623 0.0106058 55.683 < 2e-16 ***

gly_controlTRUE 0.9953449 0.0345156 28.838 < 2e-16 ***

obesityTRUE 0.5048982 0.0110805 45.566 < 2e-16 ***

smoking_status 0.3238079 0.0074753 43.317 < 2e-16 ***

---

Signif. codes: 0 ‘***’ 0.001 ‘**’ 0.01 ‘*’ 0.05 ‘.’ 0.1 ‘ ’ 1

(Dispersion parameter for binomial family taken to be 1)

Null deviance: 301057 on 443107 degrees of freedom

Residual deviance: 264272 on 443088 degrees of freedom

(45112 observations deleted due to missingness)

AIC: 264312

Number of Fisher Scoring iterations: 6

cad.chronic.hd

=========================

Call:

glm(formula = rf, family = binomial(link = "logit"), data = ms,

maxit = 100)

Deviance Residuals:

Min 1Q Median 3Q Max

-1.7148 -0.4929 -0.3464 -0.2302 3.1449

Coefficients:

Estimate Std. Error z value Pr(>|z|)

(Intercept) -7.3279137 0.0449738 -162.937 < 2e-16 ***

old.nompo 0.0154862 0.0052377 2.957 0.00311 **

rvMPOrec 0.0006121 0.0197587 0.031 0.97529

PC1 0.0001137 0.0001266 0.898 0.36937

PC2 -0.0022083 0.0002338 -9.446 < 2e-16 ***

PC3 0.0064874 0.0003967 16.355 < 2e-16 ***

PC4 0.0046970 0.0005114 9.184 < 2e-16 ***

PC5 0.0057794 0.0006703 8.622 < 2e-16 ***

PC6 -0.0018619 0.0013697 -1.359 0.17404

PC7 -0.0001693 0.0011132 -0.152 0.87912

PC8 0.0026616 0.0012187 2.184 0.02896 *

PC9 0.0103587 0.0011875 8.723 < 2e-16 ***

PC10 0.0064474 0.0012250 5.263 1.42e-07 ***

sexMale 0.7942508 0.0113700 69.855 < 2e-16 ***

age 0.0642475 0.0007240 88.734 < 2e-16 ***

hypertensionTRUE 0.4152451 0.0116905 35.520 < 2e-16 ***

hyperlipidemiaTRUE 0.5891025 0.0107370 54.867 < 2e-16 ***

gly_controlTRUE 1.0082526 0.0346298 29.115 < 2e-16 ***

obesityTRUE 0.5045102 0.0112004 45.044 < 2e-16 ***

smoking_status 0.3236521 0.0075647 42.785 < 2e-16 ***

---

Signif. codes: 0 ‘***’ 0.001 ‘**’ 0.01 ‘*’ 0.05 ‘.’ 0.1 ‘ ’ 1

(Dispersion parameter for binomial family taken to be 1)

Null deviance: 295394 on 443107 degrees of freedom

Residual deviance: 259378 on 443088 degrees of freedom

(45112 observations deleted due to missingness)

AIC: 259418

Number of Fisher Scoring iterations: 6

### New score and MPO burden

stroke.srhd.subarachnoid

=========================

Call:

glm(formula = rf, family = binomial(link = "logit"), data = ms,

maxit = 100)

Deviance Residuals:

Min 1Q Median 3Q Max

-0.1753 -0.0943 -0.0815 -0.0708 3.8070

Coefficients:

Estimate Std. Error z value Pr(>|z|)

(Intercept) -6.7732654 0.1890076 -35.836 < 2e-16 ***

new.nompo 0.0204208 0.0254657 0.802 0.422615

rvMPOrec -0.0390229 0.0967844 -0.403 0.686805

PC1 -0.0002941 0.0005449 -0.540 0.589323

PC2 0.0008510 0.0010885 0.782 0.434359

PC3 -0.0002334 0.0020300 -0.115 0.908444

PC4 0.0100181 0.0029954 3.345 0.000824 ***

PC5 0.0059196 0.0031641 1.871 0.061367 .

PC6 -0.0134674 0.0089072 -1.512 0.130542

PC7 -0.0129629 0.0063380 -2.045 0.040830 *

PC8 -0.0044225 0.0069949 -0.632 0.527231

PC9 0.0006491 0.0055481 0.117 0.906864

PC10 0.0008020 0.0070477 0.114 0.909400

sexMale -0.4433333 0.0536510 -8.263 < 2e-16 ***

age 0.0157253 0.0032333 4.864 1.15e-06 ***

hypertensionTRUE 0.3674184 0.0542678 6.770 1.28e-11 ***

hyperlipidemiaTRUE -0.0460850 0.0539611 -0.854 0.393082

gly_controlTRUE -0.4422576 0.3040735 -1.454 0.145824

obesityTRUE -0.0790648 0.0593821 -1.331 0.183038

smoking_status 0.4097687 0.0347025 11.808 < 2e-16 ***

---

Signif. codes: 0 ‘***’ 0.001 ‘**’ 0.01 ‘*’ 0.05 ‘.’ 0.1 ‘ ’ 1

(Dispersion parameter for binomial family taken to be 1)

Null deviance: 21381 on 443107 degrees of freedom

Residual deviance: 21090 on 443088 degrees of freedom

(45112 observations deleted due to missingness)

AIC: 21130

Number of Fisher Scoring iterations: 9

stroke.hd.subarachnoid

=========================

Call:

glm(formula = rf, family = binomial(link = "logit"), data = ms,

maxit = 100)

Deviance Residuals:

Min 1Q Median 3Q Max

-0.1756 -0.0871 -0.0754 -0.0649 3.8157

Coefficients:

Estimate Std. Error z value Pr(>|z|)

(Intercept) -6.789e+00 2.027e-01 -33.497 < 2e-16 ***

new.nompo 1.439e-02 2.746e-02 0.524 0.60024

rvMPOrec -2.133e-02 1.037e-01 -0.206 0.83702

PC1 4.737e-05 5.609e-04 0.084 0.93270

PC2 6.590e-04 1.113e-03 0.592 0.55393

PC3 -5.519e-04 2.098e-03 -0.263 0.79250

PC4 9.413e-03 3.160e-03 2.979 0.00289 **

PC5 8.642e-03 3.361e-03 2.571 0.01014 *

PC6 -1.325e-02 9.560e-03 -1.386 0.16589

PC7 -1.154e-02 6.650e-03 -1.736 0.08265 .

PC8 -1.420e-03 7.451e-03 -0.191 0.84887

PC9 8.556e-03 6.215e-03 1.377 0.16863

PC10 2.807e-04 7.517e-03 0.037 0.97022

sexMale -5.021e-01 5.819e-02 -8.628 < 2e-16 ***

age 1.355e-02 3.475e-03 3.899 9.65e-05 ***

hypertensionTRUE 3.644e-01 5.839e-02 6.241 4.35e-10 ***

hyperlipidemiaTRUE -3.968e-02 5.827e-02 -0.681 0.49585

gly_controlTRUE -3.671e-01 3.190e-01 -1.151 0.24989

obesityTRUE -1.167e-01 6.463e-02 -1.806 0.07100 .

smoking_status 4.378e-01 3.716e-02 11.781 < 2e-16 ***

---

Signif. codes: 0 ‘***’ 0.001 ‘**’ 0.01 ‘*’ 0.05 ‘.’ 0.1 ‘ ’ 1

(Dispersion parameter for binomial family taken to be 1)

Null deviance: 18812 on 443107 degrees of freedom

Residual deviance: 18536 on 443088 degrees of freedom

(45112 observations deleted due to missingness)

AIC: 18576

Number of Fisher Scoring iterations: 9

stroke.srhd.intracerebral

=========================

Call:

glm(formula = rf, family = binomial(link = "logit"), data = ms,

maxit = 100)

Deviance Residuals:

Min 1Q Median 3Q Max

-0.2521 -0.1091 -0.0865 -0.0674 3.8250

Coefficients:

Estimate Std. Error z value Pr(>|z|)

(Intercept) -9.2366814 0.1926196 -47.953 < 2e-16 ***

new.nompo -0.0087135 0.0232172 -0.375 0.707435

rvMPOrec -0.0565634 0.0888179 -0.637 0.524224

PC1 0.0007215 0.0004882 1.478 0.139492

PC2 -0.0012297 0.0008223 -1.495 0.134816

PC3 -0.0003673 0.0015300 -0.240 0.810280

PC4 0.0052949 0.0024733 2.141 0.032287 *

PC5 0.0068526 0.0029252 2.343 0.019151 *

PC6 -0.0057630 0.0060050 -0.960 0.337202

PC7 0.0030169 0.0053756 0.561 0.574648

PC8 -0.0029932 0.0057433 -0.521 0.602252

PC9 0.0026813 0.0051027 0.525 0.599251

PC10 -0.0044220 0.0057791 -0.765 0.444173

sexMale 0.2575895 0.0481606 5.349 8.87e-08 ***

age 0.0560312 0.0031564 17.752 < 2e-16 ***

hypertensionTRUE 0.5012631 0.0518089 9.675 < 2e-16 ***

hyperlipidemiaTRUE -0.0855529 0.0481440 -1.777 0.075565 .

gly_controlTRUE 0.4250945 0.1706450 2.491 0.012735 *

obesityTRUE -0.0245484 0.0531310 -0.462 0.644056

smoking_status 0.1291755 0.0340075 3.798 0.000146 ***

---

Signif. codes: 0 ‘***’ 0.001 ‘**’ 0.01 ‘*’ 0.05 ‘.’ 0.1 ‘ ’ 1

(Dispersion parameter for binomial family taken to be 1)

Null deviance: 25042 on 443107 degrees of freedom

Residual deviance: 24359 on 443088 degrees of freedom

(45112 observations deleted due to missingness)

AIC: 24399

Number of Fisher Scoring iterations: 8

stroke.hd.intracerebral

=========================

Call:

glm(formula = rf, family = binomial(link = "logit"), data = ms,

maxit = 100)

Deviance Residuals:

Min 1Q Median 3Q Max

-0.2548 -0.1047 -0.0816 -0.0627 3.8064

Coefficients:

Estimate Std. Error z value Pr(>|z|)

(Intercept) -9.4859740 0.2033632 -46.645 < 2e-16 ***

new.nompo -0.0246878 0.0242926 -1.016 0.30950

rvMPOrec -0.0975112 0.0942686 -1.034 0.30095

PC1 0.0006858 0.0005190 1.321 0.18636

PC2 -0.0010774 0.0008663 -1.244 0.21362

PC3 -0.0009652 0.0016258 -0.594 0.55271

PC4 0.0054286 0.0026452 2.052 0.04015 *

PC5 0.0065365 0.0030732 2.127 0.03343 *

PC6 -0.0068742 0.0066671 -1.031 0.30251

PC7 0.0054232 0.0056509 0.960 0.33720

PC8 -0.0020285 0.0061823 -0.328 0.74282

PC9 0.0014315 0.0053208 0.269 0.78790

PC10 -0.0083690 0.0061023 -1.371 0.17024

sexMale 0.2711998 0.0504739 5.373 7.74e-08 ***

age 0.0578442 0.0033234 17.405 < 2e-16 ***

hypertensionTRUE 0.5696817 0.0548599 10.384 < 2e-16 ***

hyperlipidemiaTRUE -0.1005744 0.0503655 -1.997 0.04584 *

gly_controlTRUE 0.4639715 0.1756838 2.641 0.00827 **

obesityTRUE -0.0569995 0.0559133 -1.019 0.30800

smoking_status 0.1393848 0.0355741 3.918 8.92e-05 ***

---

Signif. codes: 0 ‘***’ 0.001 ‘**’ 0.01 ‘*’ 0.05 ‘.’ 0.1 ‘ ’ 1

(Dispersion parameter for binomial family taken to be 1)

Null deviance: 23183 on 443107 degrees of freedom

Residual deviance: 22486 on 443088 degrees of freedom

(45112 observations deleted due to missingness)

AIC: 22526

Number of Fisher Scoring iterations: 9

stroke.srhd.ischaemic

=========================

Call:

glm(formula = rf, family = binomial(link = "logit"), data = ms,

maxit = 100)

Deviance Residuals:

Min 1Q Median 3Q Max

-0.8473 -0.2285 -0.1719 -0.1259 3.5281

Coefficients:

Estimate Std. Error z value Pr(>|z|)

(Intercept) -8.6281742 0.0950730 -90.753 < 2e-16 ***

new.nompo 0.0076812 0.0110382 0.696 0.486506

rvMPOrec 0.0009002 0.0413315 0.022 0.982624

PC1 0.0008303 0.0002222 3.737 0.000186 ***

PC2 0.0011722 0.0005211 2.249 0.024492 *

PC3 0.0013245 0.0009287 1.426 0.153842

PC4 0.0074846 0.0012292 6.089 1.13e-09 ***

PC5 0.0066408 0.0013827 4.803 1.57e-06 ***

PC6 -0.0114071 0.0043416 -2.627 0.008604 **

PC7 -0.0027830 0.0025707 -1.083 0.278982

PC8 0.0068905 0.0031799 2.167 0.030241 *

PC9 0.0145459 0.0025563 5.690 1.27e-08 ***

PC10 0.0023662 0.0028601 0.827 0.408063

sexMale 0.4286313 0.0235023 18.238 < 2e-16 ***

age 0.0640268 0.0015361 41.683 < 2e-16 ***

hypertensionTRUE 0.4980640 0.0252005 19.764 < 2e-16 ***

hyperlipidemiaTRUE 0.1487472 0.0226545 6.566 5.17e-11 ***

gly_controlTRUE 1.0281980 0.0598640 17.176 < 2e-16 ***

obesityTRUE 0.2922064 0.0236013 12.381 < 2e-16 ***

smoking_status 0.2952851 0.0158364 18.646 < 2e-16 ***

---

Signif. codes: 0 ‘***’ 0.001 ‘**’ 0.01 ‘*’ 0.05 ‘.’ 0.1 ‘ ’ 1

(Dispersion parameter for binomial family taken to be 1)

Null deviance: 86906 on 443107 degrees of freedom

Residual deviance: 81715 on 443088 degrees of freedom

(45112 observations deleted due to missingness)

AIC: 81755

Number of Fisher Scoring iterations: 7

stroke.hd.ischaemic

=========================

Call:

glm(formula = rf, family = binomial(link = "logit"), data = ms,

maxit = 100)

Deviance Residuals:

Min 1Q Median 3Q Max

-0.8449 -0.2282 -0.1719 -0.1260 3.5255

Coefficients:

Estimate Std. Error z value Pr(>|z|)

(Intercept) -8.6160713 0.0951097 -90.591 < 2e-16 ***

new.nompo 0.0073621 0.0110499 0.666 0.505249

rvMPOrec 0.0032741 0.0413340 0.079 0.936864

PC1 0.0008312 0.0002222 3.740 0.000184 ***

PC2 0.0011891 0.0005213 2.281 0.022547 *

PC3 0.0012893 0.0009292 1.388 0.165283

PC4 0.0074257 0.0012299 6.038 1.56e-09 ***

PC5 0.0065870 0.0013844 4.758 1.95e-06 ***

PC6 -0.0114290 0.0043545 -2.625 0.008674 **

PC7 -0.0028143 0.0025719 -1.094 0.273854

PC8 0.0070334 0.0031849 2.208 0.027217 *

PC9 0.0146959 0.0025605 5.739 9.50e-09 ***

PC10 0.0027209 0.0028654 0.950 0.342333

sexMale 0.4273602 0.0235213 18.169 < 2e-16 ***

age 0.0638547 0.0015370 41.546 < 2e-16 ***

hypertensionTRUE 0.4964289 0.0252137 19.689 < 2e-16 ***

hyperlipidemiaTRUE 0.1464276 0.0226794 6.456 1.07e-10 ***

gly_controlTRUE 1.0327125 0.0598641 17.251 < 2e-16 ***

obesityTRUE 0.2888055 0.0236399 12.217 < 2e-16 ***

smoking_status 0.2953704 0.0158504 18.635 < 2e-16 ***

---

Signif. codes: 0 ‘***’ 0.001 ‘**’ 0.01 ‘*’ 0.05 ‘.’ 0.1 ‘ ’ 1

(Dispersion parameter for binomial family taken to be 1)

Null deviance: 86750 on 443107 degrees of freedom

Residual deviance: 81597 on 443088 degrees of freedom

(45112 observations deleted due to missingness)

AIC: 81637

Number of Fisher Scoring iterations: 7

stroke.srhd.all

=========================

Call:

glm(formula = rf, family = binomial(link = "logit"), data = ms,

maxit = 100)

Deviance Residuals:

Min 1Q Median 3Q Max

-0.8796 -0.3032 -0.2355 -0.1765 3.2980

Coefficients:

Estimate Std. Error z value Pr(>|z|)

(Intercept) -7.5870838 0.0706216 -107.433 < 2e-16 ***

new.nompo 0.0076229 0.0083953 0.908 0.363883

rvMPOrec -0.0233416 0.0317083 -0.736 0.461647

PC1 0.0006678 0.0001723 3.875 0.000106 ***

PC2 0.0007002 0.0003766 1.860 0.062954 .

PC3 0.0010757 0.0006767 1.590 0.111913

PC4 0.0076394 0.0009267 8.244 < 2e-16 ***

PC5 0.0083405 0.0010463 7.972 1.57e-15 ***

PC6 -0.0064157 0.0026561 -2.415 0.015718 *

PC7 -0.0022560 0.0019469 -1.159 0.246561

PC8 0.0025784 0.0021815 1.182 0.237229

PC9 0.0092876 0.0018879 4.920 8.68e-07 ***

PC10 0.0031142 0.0021728 1.433 0.151774

sexMale 0.2910324 0.0175804 16.554 < 2e-16 ***

age 0.0589315 0.0011504 51.227 < 2e-16 ***

hypertensionTRUE 0.4175192 0.0186711 22.362 < 2e-16 ***

hyperlipidemiaTRUE 0.1545472 0.0172304 8.969 < 2e-16 ***

gly_controlTRUE 0.8640614 0.0506357 17.064 < 2e-16 ***

obesityTRUE 0.2844331 0.0180575 15.752 < 2e-16 ***

smoking_status 0.3129608 0.0119723 26.140 < 2e-16 ***

---

Signif. codes: 0 ‘***’ 0.001 ‘**’ 0.01 ‘*’ 0.05 ‘.’ 0.1 ‘ ’ 1

(Dispersion parameter for binomial family taken to be 1)

Null deviance: 135281 on 443107 degrees of freedom

Residual deviance: 128043 on 443088 degrees of freedom

(45112 observations deleted due to missingness)

AIC: 128083

Number of Fisher Scoring iterations: 6

stroke.hd.all

=========================

Call:

glm(formula = rf, family = binomial(link = "logit"), data = ms,

maxit = 100)

Deviance Residuals:

Min 1Q Median 3Q Max

-0.8230 -0.2590 -0.2008 -0.1518 3.3832

Coefficients:

Estimate Std. Error z value Pr(>|z|)

(Intercept) -7.8017072 0.0821762 -94.939 < 2e-16 ***

new.nompo 0.0058295 0.0097979 0.595 0.55186

rvMPOrec -0.0210657 0.0369889 -0.570 0.56901

PC1 0.0008129 0.0001955 4.159 3.20e-05 ***

PC2 0.0007121 0.0004262 1.671 0.09473 .

PC3 0.0007361 0.0007709 0.955 0.33964

PC4 0.0076424 0.0010895 7.015 2.31e-12 ***

PC5 0.0071746 0.0012248 5.858 4.69e-09 ***

PC6 -0.0114617 0.0037025 -3.096 0.00196 **

PC7 -0.0031273 0.0022821 -1.370 0.17057

PC8 0.0054993 0.0027642 1.989 0.04665 *

PC9 0.0125958 0.0022494 5.600 2.15e-08 ***

PC10 0.0007557 0.0025305 0.299 0.76523

sexMale 0.3083634 0.0205454 15.009 < 2e-16 ***

age 0.0570124 0.0013400 42.547 < 2e-16 ***

hypertensionTRUE 0.4848577 0.0220089 22.030 < 2e-16 ***

hyperlipidemiaTRUE 0.0967287 0.0201382 4.803 1.56e-06 ***

gly_controlTRUE 0.9250776 0.0570278 16.222 < 2e-16 ***

obesityTRUE 0.2090683 0.0212803 9.825 < 2e-16 ***

smoking_status 0.2928196 0.0139901 20.931 < 2e-16 ***

---

Signif. codes: 0 ‘***’ 0.001 ‘**’ 0.01 ‘*’ 0.05 ‘.’ 0.1 ‘ ’ 1

(Dispersion parameter for binomial family taken to be 1)

Null deviance: 105309 on 443107 degrees of freedom

Residual deviance: 100117 on 443088 degrees of freedom

(45112 observations deleted due to missingness)

AIC: 100157

Number of Fisher Scoring iterations: 7

cad.nochronic.withsr

=========================

Call:

glm(formula = rf, family = binomial(link = "logit"), data = ms,

maxit = 100)

Deviance Residuals:

Min 1Q Median 3Q Max

-1.5506 -0.4102 -0.2782 -0.1866 3.2991

Coefficients:

Estimate Std. Error z value Pr(>|z|)

(Intercept) -7.5748273 0.0522590 -144.948 < 2e-16 ***

new.nompo 0.0018352 0.0061544 0.298 0.765549

rvMPOrec -0.0114669 0.0231826 -0.495 0.620860

PC1 -0.0002007 0.0001588 -1.264 0.206364

PC2 -0.0022892 0.0002764 -8.282 < 2e-16 ***

PC3 0.0061564 0.0004633 13.289 < 2e-16 ***

PC4 0.0048743 0.0005968 8.167 3.15e-16 ***

PC5 0.0067632 0.0007764 8.711 < 2e-16 ***

PC6 -0.0018910 0.0016980 -1.114 0.265429

PC7 -0.0020597 0.0012877 -1.599 0.109715

PC8 0.0048625 0.0014384 3.381 0.000724 ***

PC9 0.0107493 0.0013903 7.731 1.06e-14 ***

PC10 0.0038372 0.0014098 2.722 0.006494 **

sexMale 0.9756055 0.0138985 70.195 < 2e-16 ***

age 0.0576018 0.0008398 68.594 < 2e-16 ***

hypertensionTRUE 0.4576843 0.0139604 32.784 < 2e-16 ***

hyperlipidemiaTRUE 0.7836830 0.0127475 61.477 < 2e-16 ***

gly_controlTRUE 0.9100010 0.0379997 23.948 < 2e-16 ***

obesityTRUE 0.3964816 0.0130677 30.340 < 2e-16 ***

smoking_status 0.3309286 0.0087729 37.722 < 2e-16 ***

---

Signif. codes: 0 ‘***’ 0.001 ‘**’ 0.01 ‘*’ 0.05 ‘.’ 0.1 ‘ ’ 1

(Dispersion parameter for binomial family taken to be 1)

Null deviance: 232155 on 443107 degrees of freedom

Residual deviance: 201669 on 443088 degrees of freedom

(45112 observations deleted due to missingness)

AIC: 201709

Number of Fisher Scoring iterations: 6

cad.nochronic.hd

=========================

Call:

glm(formula = rf, family = binomial(link = "logit"), data = ms,

maxit = 100)

Deviance Residuals:

Min 1Q Median 3Q Max

-1.5138 -0.3956 -0.2687 -0.1814 3.3293

Coefficients:

Estimate Std. Error z value Pr(>|z|)

(Intercept) -7.5672992 0.0539200 -140.343 < 2e-16 ***

new.nompo -0.0024027 0.0063643 -0.378 0.70578

rvMPOrec -0.0032499 0.0239021 -0.136 0.89185

PC1 -0.0004505 0.0001712 -2.632 0.00849 **

PC2 -0.0023939 0.0002914 -8.215 < 2e-16 ***

PC3 0.0064270 0.0004842 13.273 < 2e-16 ***

PC4 0.0052838 0.0006219 8.496 < 2e-16 ***

PC5 0.0068691 0.0008016 8.569 < 2e-16 ***

PC6 -0.0025817 0.0017967 -1.437 0.15075

PC7 -0.0021570 0.0013476 -1.601 0.10947

PC8 0.0041308 0.0015097 2.736 0.00622 **

PC9 0.0120117 0.0014444 8.316 < 2e-16 ***

PC10 0.0042259 0.0014553 2.904 0.00369 **

sexMale 0.9641428 0.0144211 66.857 < 2e-16 ***

age 0.0560456 0.0008674 64.616 < 2e-16 ***

hypertensionTRUE 0.4734285 0.0145068 32.635 < 2e-16 ***

hyperlipidemiaTRUE 0.7727647 0.0132107 58.496 < 2e-16 ***

gly_controlTRUE 0.9146583 0.0386895 23.641 < 2e-16 ***

obesityTRUE 0.3835207 0.0135094 28.389 < 2e-16 ***

smoking_status 0.3301493 0.0090685 36.406 < 2e-16 ***

---

Signif. codes: 0 ‘***’ 0.001 ‘**’ 0.01 ‘*’ 0.05 ‘.’ 0.1 ‘ ’ 1

(Dispersion parameter for binomial family taken to be 1)

Null deviance: 219311 on 443107 degrees of freedom

Residual deviance: 191293 on 443088 degrees of freedom

(45112 observations deleted due to missingness)

AIC: 191333

Number of Fisher Scoring iterations: 6

cad.chronic.withsr

=========================

Call:

glm(formula = rf, family = binomial(link = "logit"), data = ms,

maxit = 100)

Deviance Residuals:

Min 1Q Median 3Q Max

-1.7314 -0.4995 -0.3513 -0.2331 3.1217

Coefficients:

Estimate Std. Error z value Pr(>|z|)

(Intercept) -7.2918591 0.0443965 -164.244 < 2e-16 ***

new.nompo 0.0120389 0.0052251 2.304 0.02122 *

rvMPOrec -0.0021671 0.0196248 -0.110 0.91207

PC1 0.0002125 0.0001238 1.717 0.08596 .

PC2 -0.0021623 0.0002275 -9.505 < 2e-16 ***

PC3 0.0062560 0.0003881 16.121 < 2e-16 ***

PC4 0.0046096 0.0005053 9.123 < 2e-16 ***

PC5 0.0057111 0.0006630 8.614 < 2e-16 ***

PC6 -0.0015296 0.0013429 -1.139 0.25468

PC7 -0.0002410 0.0010943 -0.220 0.82566

PC8 0.0032645 0.0011964 2.729 0.00636 **

PC9 0.0096591 0.0011712 8.247 < 2e-16 ***

PC10 0.0062624 0.0012111 5.171 2.33e-07 ***

sexMale 0.8006842 0.0112265 71.321 < 2e-16 ***

age 0.0642716 0.0007150 89.896 < 2e-16 ***

hypertensionTRUE 0.4060963 0.0115298 35.222 < 2e-16 ***

hyperlipidemiaTRUE 0.5903160 0.0106055 55.662 < 2e-16 ***

gly_controlTRUE 0.9958301 0.0345156 28.852 < 2e-16 ***

obesityTRUE 0.5050298 0.0110803 45.579 < 2e-16 ***

smoking_status 0.3237888 0.0074753 43.314 < 2e-16 ***

---

Signif. codes: 0 ‘***’ 0.001 ‘**’ 0.01 ‘*’ 0.05 ‘.’ 0.1 ‘ ’ 1

(Dispersion parameter for binomial family taken to be 1)

Null deviance: 301057 on 443107 degrees of freedom

Residual deviance: 264276 on 443088 degrees of freedom

(45112 observations deleted due to missingness)

AIC: 264316

Number of Fisher Scoring iterations: 6

cad.chronic.hd

=========================

Call:

glm(formula = rf, family = binomial(link = "logit"), data = ms,

maxit = 100)

Deviance Residuals:

Min 1Q Median 3Q Max

-1.7238 -0.4928 -0.3464 -0.2303 3.1452

Coefficients:

Estimate Std. Error z value Pr(>|z|)

(Intercept) -7.3278925 0.0449737 -162.937 < 2e-16 ***

new.nompo 0.0105108 0.0052862 1.988 0.0468 *

rvMPOrec 0.0016817 0.0198286 0.085 0.9324

PC1 0.0001018 0.0001273 0.799 0.4241

PC2 -0.0021776 0.0002334 -9.330 < 2e-16 ***

PC3 0.0064660 0.0003966 16.303 < 2e-16 ***

PC4 0.0047625 0.0005122 9.299 < 2e-16 ***

PC5 0.0057764 0.0006703 8.617 < 2e-16 ***

PC6 -0.0018572 0.0013703 -1.355 0.1753

PC7 -0.0001654 0.0011132 -0.149 0.8819

PC8 0.0026775 0.0012189 2.197 0.0280 *

PC9 0.0103587 0.0011874 8.724 < 2e-16 ***

PC10 0.0064310 0.0012249 5.250 1.52e-07 ***

sexMale 0.7943824 0.0113700 69.867 < 2e-16 ***

age 0.0642467 0.0007240 88.734 < 2e-16 ***

hypertensionTRUE 0.4151496 0.0116903 35.512 < 2e-16 ***

hyperlipidemiaTRUE 0.5888566 0.0107366 54.846 < 2e-16 ***

gly_controlTRUE 1.0087267 0.0346295 29.129 < 2e-16 ***

obesityTRUE 0.5046375 0.0112002 45.056 < 2e-16 ***

smoking_status 0.3236332 0.0075646 42.782 < 2e-16 ***

---

Signif. codes: 0 ‘***’ 0.001 ‘**’ 0.01 ‘*’ 0.05 ‘.’ 0.1 ‘ ’ 1

(Dispersion parameter for binomial family taken to be 1)

Null deviance: 295394 on 443107 degrees of freedom

Residual deviance: 259383 on 443088 degrees of freedom

(45112 observations deleted due to missingness)

AIC: 259423

Number of Fisher Scoring iterations: 6

### New score, stratified by serum and plasma

stroke.srhd.subarachnoid

=========================

Call:

glm(formula = rf, family = binomial(link = "logit"), data = ms,

maxit = 100)

Deviance Residuals:

Min 1Q Median 3Q Max

-0.1722 -0.0943 -0.0815 -0.0708 3.8084

Coefficients:

Estimate Std. Error z value Pr(>|z|)

(Intercept) -6.7758459 0.1888983 -35.870 < 2e-16 ***

new.plasma 0.0016773 0.0249520 0.067 0.946406

new.serum 0.0280929 0.0257176 1.092 0.274674

PC1 -0.0003228 0.0005477 -0.589 0.555649

PC2 0.0008220 0.0010877 0.756 0.449820

PC3 -0.0002186 0.0020298 -0.108 0.914250

PC4 0.0099113 0.0029937 3.311 0.000931 ***

PC5 0.0059154 0.0031638 1.870 0.061521 .

PC6 -0.0134330 0.0089111 -1.507 0.131696

PC7 -0.0129751 0.0063344 -2.048 0.040524 *

PC8 -0.0044740 0.0069954 -0.640 0.522456

PC9 0.0007179 0.0055480 0.129 0.897038

PC10 0.0007596 0.0070458 0.108 0.914143

sexMale -0.4433002 0.0536501 -8.263 < 2e-16 ***

age 0.0157148 0.0032334 4.860 1.17e-06 ***

hypertensionTRUE 0.3674539 0.0542675 6.771 1.28e-11 ***

hyperlipidemiaTRUE -0.0460047 0.0539615 -0.853 0.393911

gly_controlTRUE -0.4415188 0.3040748 -1.452 0.146500

obesityTRUE -0.0788487 0.0593833 -1.328 0.184247

smoking_status 0.4098748 0.0347019 11.811 < 2e-16 ***

---

Signif. codes: 0 ‘***’ 0.001 ‘**’ 0.01 ‘*’ 0.05 ‘.’ 0.1 ‘ ’ 1

(Dispersion parameter for binomial family taken to be 1)

Null deviance: 21381 on 443107 degrees of freedom

Residual deviance: 21090 on 443088 degrees of freedom

(45112 observations deleted due to missingness)

AIC: 21130

Number of Fisher Scoring iterations: 9

stroke.hd.subarachnoid

=========================

Call:

glm(formula = rf, family = binomial(link = "logit"), data = ms,

maxit = 100)

Deviance Residuals:

Min 1Q Median 3Q Max

-0.1746 -0.0871 -0.0754 -0.0649 3.8161

Coefficients:

Estimate Std. Error z value Pr(>|z|)

(Intercept) -6.7904012 0.2025555 -33.524 < 2e-16 ***

new.plasma 0.0028745 0.0269317 0.107 0.91500

new.serum 0.0153316 0.0276271 0.555 0.57893

PC1 0.0000380 0.0005641 0.067 0.94629

PC2 0.0006524 0.0011123 0.587 0.55754

PC3 -0.0005444 0.0020979 -0.259 0.79526

PC4 0.0093483 0.0031590 2.959 0.00308 **

PC5 0.0086417 0.0033610 2.571 0.01014 *

PC6 -0.0132307 0.0095624 -1.384 0.16648

PC7 -0.0115459 0.0066477 -1.737 0.08242 .

PC8 -0.0014498 0.0074515 -0.195 0.84573

PC9 0.0085898 0.0062154 1.382 0.16697

PC10 0.0002591 0.0075156 0.034 0.97249

sexMale -0.5020541 0.0581878 -8.628 < 2e-16 ***

age 0.0135438 0.0034749 3.898 9.71e-05 ***

hypertensionTRUE 0.3644010 0.0583878 6.241 4.35e-10 ***

hyperlipidemiaTRUE -0.0396315 0.0582656 -0.680 0.49639

gly_controlTRUE -0.3667503 0.3190214 -1.150 0.25030

obesityTRUE -0.1165861 0.0646332 -1.804 0.07126 .

smoking_status 0.4378425 0.0371616 11.782 < 2e-16 ***

---

Signif. codes: 0 ‘***’ 0.001 ‘**’ 0.01 ‘*’ 0.05 ‘.’ 0.1 ‘ ’ 1

(Dispersion parameter for binomial family taken to be 1)

Null deviance: 18812 on 443107 degrees of freedom

Residual deviance: 18536 on 443088 degrees of freedom

(45112 observations deleted due to missingness)

AIC: 18576

Number of Fisher Scoring iterations: 9

stroke.srhd.intracerebral

=========================

Call:

glm(formula = rf, family = binomial(link = "logit"), data = ms,

maxit = 100)

Deviance Residuals:

Min 1Q Median 3Q Max

-0.2509 -0.1091 -0.0865 -0.0674 3.8235

Coefficients:

Estimate Std. Error z value Pr(>|z|)

(Intercept) -9.2407915 0.1925241 -47.998 < 2e-16 ***

new.plasma -0.0132684 0.0226502 -0.586 0.558013

new.serum 0.0046682 0.0232912 0.200 0.841146

PC1 0.0007141 0.0004908 1.455 0.145729

PC2 -0.0012789 0.0008214 -1.557 0.119483

PC3 -0.0003419 0.0015298 -0.223 0.823177

PC4 0.0052762 0.0024721 2.134 0.032816 *

PC5 0.0068813 0.0029250 2.353 0.018642 *

PC6 -0.0057250 0.0060082 -0.953 0.340659

PC7 0.0030091 0.0053736 0.560 0.575489

PC8 -0.0029785 0.0057435 -0.519 0.604056

PC9 0.0027240 0.0051026 0.534 0.593453

PC10 -0.0044262 0.0057777 -0.766 0.443630

sexMale 0.2576837 0.0481600 5.351 8.77e-08 ***

age 0.0560284 0.0031564 17.751 < 2e-16 ***

hypertensionTRUE 0.5012111 0.0518085 9.674 < 2e-16 ***

hyperlipidemiaTRUE -0.0855735 0.0481435 -1.777 0.075491 .

gly_controlTRUE 0.4256509 0.1706464 2.494 0.012619 *

obesityTRUE -0.0243652 0.0531315 -0.459 0.646534

smoking_status 0.1292567 0.0340068 3.801 0.000144 ***

---

Signif. codes: 0 ‘***’ 0.001 ‘**’ 0.01 ‘*’ 0.05 ‘.’ 0.1 ‘ ’ 1

(Dispersion parameter for binomial family taken to be 1)

Null deviance: 25042 on 443107 degrees of freedom

Residual deviance: 24359 on 443088 degrees of freedom

(45112 observations deleted due to missingness)

AIC: 24399

Number of Fisher Scoring iterations: 8

stroke.hd.intracerebral

=========================

Call:

glm(formula = rf, family = binomial(link = "logit"), data = ms,

maxit = 100)

Deviance Residuals:

Min 1Q Median 3Q Max

-0.2534 -0.1047 -0.0816 -0.0627 3.8130

Coefficients:

Estimate Std. Error z value Pr(>|z|)

(Intercept) -9.4929042 0.2032689 -46.701 < 2e-16 ***

new.plasma -0.0170243 0.0236532 -0.720 0.47168

new.serum -0.0069569 0.0243082 -0.286 0.77473

PC1 0.0006865 0.0005216 1.316 0.18813

PC2 -0.0011517 0.0008654 -1.331 0.18324

PC3 -0.0009277 0.0016257 -0.571 0.56823

PC4 0.0054704 0.0026442 2.069 0.03856 *

PC5 0.0066002 0.0030730 2.148 0.03173 *

PC6 -0.0068289 0.0066703 -1.024 0.30594

PC7 0.0054343 0.0056499 0.962 0.33613

PC8 -0.0019537 0.0061828 -0.316 0.75201

PC9 0.0014677 0.0053211 0.276 0.78267

PC10 -0.0083626 0.0061016 -1.371 0.17051

sexMale 0.2714019 0.0504728 5.377 7.57e-08 ***

age 0.0578440 0.0033234 17.405 < 2e-16 ***

hypertensionTRUE 0.5695673 0.0548585 10.382 < 2e-16 ***

hyperlipidemiaTRUE -0.1006334 0.0503641 -1.998 0.04570 *

gly_controlTRUE 0.4644998 0.1756841 2.644 0.00819 **

obesityTRUE -0.0568037 0.0559135 -1.016 0.30967

smoking_status 0.1394194 0.0355726 3.919 8.88e-05 ***

---

Signif. codes: 0 ‘***’ 0.001 ‘**’ 0.01 ‘*’ 0.05 ‘.’ 0.1 ‘ ’ 1

(Dispersion parameter for binomial family taken to be 1)

Null deviance: 23183 on 443107 degrees of freedom

Residual deviance: 22487 on 443088 degrees of freedom

(45112 observations deleted due to missingness)

AIC: 22527

Number of Fisher Scoring iterations: 9

stroke.srhd.ischaemic

=========================

Call:

glm(formula = rf, family = binomial(link = "logit"), data = ms,

maxit = 100)

Deviance Residuals:

Min 1Q Median 3Q Max

-0.8479 -0.2285 -0.1719 -0.1259 3.5281

Coefficients:

Estimate Std. Error z value Pr(>|z|)

(Intercept) -8.6280321 0.0950232 -90.799 < 2e-16 ***

new.plasma 0.0075855 0.0108580 0.699 0.484797

new.serum 0.0045428 0.0110840 0.410 0.681917

PC1 0.0008258 0.0002234 3.696 0.000219 ***

PC2 0.0011788 0.0005208 2.264 0.023604 *

PC3 0.0013190 0.0009287 1.420 0.155539

PC4 0.0074784 0.0012288 6.086 1.16e-09 ***

PC5 0.0066346 0.0013826 4.799 1.60e-06 ***

PC6 -0.0114058 0.0043410 -2.627 0.008603 **

PC7 -0.0027867 0.0025708 -1.084 0.278360

PC8 0.0068791 0.0031797 2.163 0.030505 *

PC9 0.0145463 0.0025564 5.690 1.27e-08 ***

PC10 0.0023623 0.0028601 0.826 0.408839

sexMale 0.4286330 0.0235021 18.238 < 2e-16 ***

age 0.0640252 0.0015361 41.682 < 2e-16 ***

hypertensionTRUE 0.4980870 0.0252006 19.765 < 2e-16 ***

hyperlipidemiaTRUE 0.1487747 0.0226546 6.567 5.13e-11 ***

gly_controlTRUE 1.0281601 0.0598648 17.175 < 2e-16 ***

obesityTRUE 0.2921414 0.0236018 12.378 < 2e-16 ***

smoking_status 0.2952816 0.0158365 18.646 < 2e-16 ***

---

Signif. codes: 0 ‘***’ 0.001 ‘**’ 0.01 ‘*’ 0.05 ‘.’ 0.1 ‘ ’ 1

(Dispersion parameter for binomial family taken to be 1)

Null deviance: 86906 on 443107 degrees of freedom

Residual deviance: 81715 on 443088 degrees of freedom

(45112 observations deleted due to missingness)

AIC: 81755

Number of Fisher Scoring iterations: 7

stroke.hd.ischaemic

=========================

Call:

glm(formula = rf, family = binomial(link = "logit"), data = ms,

maxit = 100)

Deviance Residuals:

Min 1Q Median 3Q Max

-0.8455 -0.2282 -0.1719 -0.1260 3.5261

Coefficients:

Estimate Std. Error z value Pr(>|z|)

(Intercept) -8.6157606 0.0950596 -90.635 < 2e-16 ***

new.plasma 0.0076577 0.0108697 0.705 0.481121

new.serum 0.0039801 0.0110943 0.359 0.719778

PC1 0.0008270 0.0002235 3.701 0.000215 ***

PC2 0.0011969 0.0005210 2.298 0.021585 *

PC3 0.0012830 0.0009292 1.381 0.167343

PC4 0.0074217 0.0012295 6.036 1.58e-09 ***

PC5 0.0065799 0.0013843 4.753 2.00e-06 ***

PC6 -0.0114284 0.0043538 -2.625 0.008667 **

PC7 -0.0028178 0.0025721 -1.096 0.273283

PC8 0.0070221 0.0031846 2.205 0.027454 *

PC9 0.0146943 0.0025606 5.739 9.55e-09 ***

PC10 0.0027175 0.0028654 0.948 0.342944

sexMale 0.4273591 0.0235212 18.169 < 2e-16 ***

age 0.0638532 0.0015370 41.545 < 2e-16 ***

hypertensionTRUE 0.4964527 0.0252137 19.690 < 2e-16 ***

hyperlipidemiaTRUE 0.1464541 0.0226795 6.458 1.06e-10 ***

gly_controlTRUE 1.0326501 0.0598650 17.250 < 2e-16 ***

obesityTRUE 0.2887363 0.0236403 12.214 < 2e-16 ***

smoking_status 0.2953636 0.0158505 18.634 < 2e-16 ***

---

Signif. codes: 0 ‘***’ 0.001 ‘**’ 0.01 ‘*’ 0.05 ‘.’ 0.1 ‘ ’ 1

(Dispersion parameter for binomial family taken to be 1)

Null deviance: 86750 on 443107 degrees of freedom

Residual deviance: 81597 on 443088 degrees of freedom

(45112 observations deleted due to missingness)

AIC: 81637

Number of Fisher Scoring iterations: 7

stroke.srhd.all

=========================

Call:

glm(formula = rf, family = binomial(link = "logit"), data = ms,

maxit = 100)

Deviance Residuals:

Min 1Q Median 3Q Max

-0.8799 -0.3032 -0.2355 -0.1765 3.2912

Coefficients:

Estimate Std. Error z value Pr(>|z|)

(Intercept) -7.5886696 0.0705857 -107.510 < 2e-16 ***

new.plasma 0.0057782 0.0082505 0.700 0.483716

new.serum 0.0051570 0.0084310 0.612 0.540751

PC1 0.0006695 0.0001733 3.864 0.000112 ***

PC2 0.0006996 0.0003763 1.859 0.062990 .

PC3 0.0010791 0.0006766 1.595 0.110745

PC4 0.0076232 0.0009264 8.229 < 2e-16 ***

PC5 0.0083502 0.0010462 7.982 1.44e-15 ***

PC6 -0.0064091 0.0026561 -2.413 0.015823 *

PC7 -0.0022515 0.0019470 -1.156 0.247509

PC8 0.0025786 0.0021815 1.182 0.237208

PC9 0.0092983 0.0018880 4.925 8.44e-07 ***

PC10 0.0031080 0.0021727 1.431 0.152573

sexMale 0.2910651 0.0175803 16.556 < 2e-16 ***

age 0.0589289 0.0011504 51.225 < 2e-16 ***

hypertensionTRUE 0.4175154 0.0186711 22.362 < 2e-16 ***

hyperlipidemiaTRUE 0.1545780 0.0172304 8.971 < 2e-16 ***

gly_controlTRUE 0.8641514 0.0506356 17.066 < 2e-16 ***

obesityTRUE 0.2844076 0.0180578 15.750 < 2e-16 ***

smoking_status 0.3129687 0.0119723 26.141 < 2e-16 ***

---

Signif. codes: 0 ‘***’ 0.001 ‘**’ 0.01 ‘*’ 0.05 ‘.’ 0.1 ‘ ’ 1

(Dispersion parameter for binomial family taken to be 1)

Null deviance: 135281 on 443107 degrees of freedom

Residual deviance: 128044 on 443088 degrees of freedom

(45112 observations deleted due to missingness)

AIC: 128084

Number of Fisher Scoring iterations: 6

stroke.hd.all

=========================

Call:

glm(formula = rf, family = binomial(link = "logit"), data = ms,

maxit = 100)

Deviance Residuals:

Min 1Q Median 3Q Max

-0.8227 -0.2590 -0.2008 -0.1518 3.3803

Coefficients:

Estimate Std. Error z value Pr(>|z|)

(Intercept) -7.8031728 0.0821337 -95.006 < 2e-16 ***

new.plasma 0.0052511 0.0096292 0.545 0.58552

new.serum 0.0059519 0.0098382 0.605 0.54519

PC1 0.0008071 0.0001966 4.105 4.04e-05 ***

PC2 0.0007043 0.0004259 1.654 0.09819 .

PC3 0.0007386 0.0007708 0.958 0.33797

PC4 0.0076333 0.0010891 7.009 2.41e-12 ***

PC5 0.0071800 0.0012247 5.863 4.55e-09 ***

PC6 -0.0114531 0.0037025 -3.093 0.00198 **

PC7 -0.0031300 0.0022820 -1.372 0.17018

PC8 0.0054976 0.0027642 1.989 0.04672 *

PC9 0.0126085 0.0022494 5.605 2.08e-08 ***

PC10 0.0007506 0.0025303 0.297 0.76673

sexMale 0.3084038 0.0205452 15.011 < 2e-16 ***

age 0.0570100 0.0013400 42.545 < 2e-16 ***

hypertensionTRUE 0.4848663 0.0220089 22.030 < 2e-16 ***

hyperlipidemiaTRUE 0.0967573 0.0201382 4.805 1.55e-06 ***

gly_controlTRUE 0.9252074 0.0570282 16.224 < 2e-16 ***

obesityTRUE 0.2090510 0.0212807 9.824 < 2e-16 ***

smoking_status 0.2928290 0.0139900 20.931 < 2e-16 ***

---

Signif. codes: 0 ‘***’ 0.001 ‘**’ 0.01 ‘*’ 0.05 ‘.’ 0.1 ‘ ’ 1

(Dispersion parameter for binomial family taken to be 1)

Null deviance: 105309 on 443107 degrees of freedom

Residual deviance: 100117 on 443088 degrees of freedom

(45112 observations deleted due to missingness)

AIC: 100157

Number of Fisher Scoring iterations: 7

cad.nochronic.withsr

=========================

Call:

glm(formula = rf, family = binomial(link = "logit"), data = ms,

maxit = 100)

Deviance Residuals:

Min 1Q Median 3Q Max

-1.5494 -0.4102 -0.2782 -0.1865 3.2981

Coefficients:

Estimate Std. Error z value Pr(>|z|)

(Intercept) -7.5756312 0.0522326 -145.037 < 2e-16 ***

new.plasma 0.0023315 0.0060408 0.386 0.699524

new.serum -0.0045529 0.0061605 -0.739 0.459881

PC1 -0.0001810 0.0001594 -1.135 0.256177

PC2 -0.0022738 0.0002762 -8.232 < 2e-16 ***

PC3 0.0061594 0.0004633 13.296 < 2e-16 ***

PC4 0.0048735 0.0005966 8.169 3.12e-16 ***

PC5 0.0067797 0.0007764 8.733 < 2e-16 ***

PC6 -0.0018977 0.0016979 -1.118 0.263708

PC7 -0.0020391 0.0012881 -1.583 0.113412

PC8 0.0048803 0.0014384 3.393 0.000692 ***

PC9 0.0107406 0.0013904 7.725 1.12e-14 ***

PC10 0.0038379 0.0014099 2.722 0.006488 **

sexMale 0.9756214 0.0138984 70.197 < 2e-16 ***

age 0.0576016 0.0008398 68.593 < 2e-16 ***

hypertensionTRUE 0.4576587 0.0139605 32.782 < 2e-16 ***

hyperlipidemiaTRUE 0.7836984 0.0127475 61.479 < 2e-16 ***

gly_controlTRUE 0.9098991 0.0379993 23.945 < 2e-16 ***

obesityTRUE 0.3964327 0.0130679 30.336 < 2e-16 ***

smoking_status 0.3309077 0.0087730 37.719 < 2e-16 ***

---

Signif. codes: 0 ‘***’ 0.001 ‘**’ 0.01 ‘*’ 0.05 ‘.’ 0.1 ‘ ’ 1

(Dispersion parameter for binomial family taken to be 1)

Null deviance: 232155 on 443107 degrees of freedom

Residual deviance: 201669 on 443088 degrees of freedom

(45112 observations deleted due to missingness)

AIC: 201709

Number of Fisher Scoring iterations: 6

cad.nochronic.hd

=========================

Call:

glm(formula = rf, family = binomial(link = "logit"), data = ms,

maxit = 100)

Deviance Residuals:

Min 1Q Median 3Q Max

-1.5119 -0.3955 -0.2687 -0.1814 3.3277

Coefficients:

Estimate Std. Error z value Pr(>|z|)

(Intercept) -7.5676254 0.0538922 -140.422 < 2e-16 ***

new.plasma 0.0007258 0.0062429 0.116 0.90745

new.serum -0.0082789 0.0063667 -1.300 0.19349

PC1 -0.0004300 0.0001717 -2.504 0.01227 *

PC2 -0.0023778 0.0002912 -8.165 3.22e-16 ***

PC3 0.0064281 0.0004842 13.276 < 2e-16 ***

PC4 0.0052988 0.0006217 8.523 < 2e-16 ***

PC5 0.0068842 0.0008016 8.588 < 2e-16 ***

PC6 -0.0025915 0.0017965 -1.443 0.14915

PC7 -0.0021353 0.0013481 -1.584 0.11320

PC8 0.0041550 0.0015098 2.752 0.00592 **

PC9 0.0119962 0.0014445 8.305 < 2e-16 ***

PC10 0.0042315 0.0014554 2.907 0.00364 **

sexMale 0.9641590 0.0144210 66.858 < 2e-16 ***

age 0.0560474 0.0008674 64.619 < 2e-16 ***

hypertensionTRUE 0.4734002 0.0145069 32.633 < 2e-16 ***

hyperlipidemiaTRUE 0.7727690 0.0132106 58.496 < 2e-16 ***

gly_controlTRUE 0.9144867 0.0386895 23.637 < 2e-16 ***

obesityTRUE 0.3834780 0.0135096 28.386 < 2e-16 ***

smoking_status 0.3301161 0.0090686 36.402 < 2e-16 ***

---

Signif. codes: 0 ‘***’ 0.001 ‘**’ 0.01 ‘*’ 0.05 ‘.’ 0.1 ‘ ’ 1

(Dispersion parameter for binomial family taken to be 1)

Null deviance: 219311 on 443107 degrees of freedom

Residual deviance: 191291 on 443088 degrees of freedom

(45112 observations deleted due to missingness)

AIC: 191331

Number of Fisher Scoring iterations: 6

cad.chronic.withsr

=========================

Call:

glm(formula = rf, family = binomial(link = "logit"), data = ms,

maxit = 100)

Deviance Residuals:

Min 1Q Median 3Q Max

-1.7370 -0.4995 -0.3513 -0.2331 3.1213

Coefficients:

Estimate Std. Error z value Pr(>|z|)

(Intercept) -7.2918369 0.0443735 -164.329 < 2e-16 ***

new.plasma 0.0099265 0.0051422 1.930 0.05356 .

new.serum 0.0018367 0.0052347 0.351 0.72569

PC1 0.0002267 0.0001243 1.824 0.06811 .

PC2 -0.0021354 0.0002273 -9.394 < 2e-16 ***

PC3 0.0062516 0.0003880 16.110 < 2e-16 ***

PC4 0.0045908 0.0005051 9.090 < 2e-16 ***

PC5 0.0057141 0.0006629 8.619 < 2e-16 ***

PC6 -0.0015413 0.0013426 -1.148 0.25098

PC7 -0.0002280 0.0010945 -0.208 0.83496

PC8 0.0032612 0.0011964 2.726 0.00641 **

PC9 0.0096513 0.0011713 8.240 < 2e-16 ***

PC10 0.0062570 0.0012111 5.166 2.39e-07 ***

sexMale 0.8006634 0.0112264 71.320 < 2e-16 ***

age 0.0642695 0.0007150 89.894 < 2e-16 ***

hypertensionTRUE 0.4060892 0.0115298 35.221 < 2e-16 ***

hyperlipidemiaTRUE 0.5903562 0.0106054 55.665 < 2e-16 ***

gly_controlTRUE 0.9956636 0.0345151 28.847 < 2e-16 ***

obesityTRUE 0.5049208 0.0110805 45.569 < 2e-16 ***

smoking_status 0.3237745 0.0074753 43.312 < 2e-16 ***

---

Signif. codes: 0 ‘***’ 0.001 ‘**’ 0.01 ‘*’ 0.05 ‘.’ 0.1 ‘ ’ 1

(Dispersion parameter for binomial family taken to be 1)

Null deviance: 301057 on 443107 degrees of freedom

Residual deviance: 264277 on 443088 degrees of freedom

(45112 observations deleted due to missingness)

AIC: 264317

Number of Fisher Scoring iterations: 6

cad.chronic.hd

=========================

Call:

glm(formula = rf, family = binomial(link = "logit"), data = ms,

maxit = 100)

Deviance Residuals:

Min 1Q Median 3Q Max

-1.7304 -0.4929 -0.3464 -0.2303 3.1444

Coefficients:

Estimate Std. Error z value Pr(>|z|)

(Intercept) -7.3276436 0.0449503 -163.016 < 2e-16 ***

new.plasma 0.0096789 0.0052017 1.861 0.0628 .

new.serum 0.0003832 0.0052951 0.072 0.9423

PC1 0.0001159 0.0001278 0.907 0.3645

PC2 -0.0021501 0.0002332 -9.219 < 2e-16 ***

PC3 0.0064604 0.0003966 16.289 < 2e-16 ***

PC4 0.0047508 0.0005120 9.279 < 2e-16 ***

PC5 0.0057783 0.0006703 8.621 < 2e-16 ***

PC6 -0.0018705 0.0013700 -1.365 0.1722

PC7 -0.0001520 0.0011135 -0.136 0.8914

PC8 0.0026765 0.0012189 2.196 0.0281 *

PC9 0.0103474 0.0011875 8.714 < 2e-16 ***

PC10 0.0064277 0.0012250 5.247 1.55e-07 ***

sexMale 0.7943632 0.0113699 69.865 < 2e-16 ***

age 0.0642455 0.0007240 88.733 < 2e-16 ***

hypertensionTRUE 0.4151434 0.0116903 35.512 < 2e-16 ***

hyperlipidemiaTRUE 0.5888933 0.0107366 54.849 < 2e-16 ***

gly_controlTRUE 1.0085319 0.0346293 29.124 < 2e-16 ***

obesityTRUE 0.5045252 0.0112004 45.045 < 2e-16 ***

smoking_status 0.3236133 0.0075647 42.779 < 2e-16 ***

---

Signif. codes: 0 ‘***’ 0.001 ‘**’ 0.01 ‘*’ 0.05 ‘.’ 0.1 ‘ ’ 1

(Dispersion parameter for binomial family taken to be 1)

Null deviance: 295394 on 443107 degrees of freedom

Residual deviance: 259383 on 443088 degrees of freedom

(45112 observations deleted due to missingness)

AIC: 259423

Number of Fisher Scoring iterations: 6

### Updated score

stroke.srhd.subarachnoid

=========================

Call:

glm(formula = rf, family = binomial(link = "logit"), data = ms,

maxit = 100)

Deviance Residuals:

Min 1Q Median 3Q Max

-0.1801 -0.0942 -0.0815 -0.0708 3.8040

Coefficients:

Estimate Std. Error z value Pr(>|z|)

(Intercept) -6.7757165 0.1888992 -35.869 < 2e-16 ***

updated 0.0026335 0.0251315 0.105 0.916544

PC1 -0.0002354 0.0005448 -0.432 0.665693

PC2 0.0009067 0.0010849 0.836 0.403309

PC3 -0.0002179 0.0020303 -0.107 0.914542

PC4 0.0099171 0.0029939 3.312 0.000925 ***

PC5 0.0059660 0.0031637 1.886 0.059329 .

PC6 -0.0134752 0.0089116 -1.512 0.130512

PC7 -0.0129273 0.0063403 -2.039 0.041458 *

PC8 -0.0044186 0.0069969 -0.632 0.527703

PC9 0.0006582 0.0055483 0.119 0.905573

PC10 0.0007971 0.0070474 0.113 0.909947

sexMale -0.4433975 0.0536502 -8.265 < 2e-16 ***

age 0.0157235 0.0032334 4.863 1.16e-06 ***

hypertensionTRUE 0.3673278 0.0542684 6.769 1.30e-11 ***

hyperlipidemiaTRUE -0.0460419 0.0539608 -0.853 0.393523

gly_controlTRUE -0.4426021 0.3040715 -1.456 0.145507

obesityTRUE -0.0790506 0.0593827 -1.331 0.183121

smoking_status 0.4098226 0.0347025 11.810 < 2e-16 ***

---

Signif. codes: 0 ‘***’ 0.001 ‘**’ 0.01 ‘*’ 0.05 ‘.’ 0.1 ‘ ’ 1

(Dispersion parameter for binomial family taken to be 1)

Null deviance: 21381 on 443107 degrees of freedom

Residual deviance: 21091 on 443089 degrees of freedom

(45112 observations deleted due to missingness)

AIC: 21129

Number of Fisher Scoring iterations: 9

stroke.hd.subarachnoid

=========================

Call:

glm(formula = rf, family = binomial(link = "logit"), data = ms,

maxit = 100)

Deviance Residuals:

Min 1Q Median 3Q Max

-0.1784 -0.0871 -0.0754 -0.0649 3.8137

Coefficients:

Estimate Std. Error z value Pr(>|z|)

(Intercept) -6.791e+00 2.026e-01 -33.524 < 2e-16 ***

updated 1.079e-02 2.734e-02 0.395 0.69316

PC1 6.209e-05 5.608e-04 0.111 0.91184

PC2 6.964e-04 1.109e-03 0.628 0.53010

PC3 -5.603e-04 2.098e-03 -0.267 0.78944

PC4 9.364e-03 3.158e-03 2.965 0.00303 **

PC5 8.671e-03 3.361e-03 2.580 0.00988 **

PC6 -1.326e-02 9.564e-03 -1.386 0.16574

PC7 -1.154e-02 6.651e-03 -1.735 0.08271 .

PC8 -1.426e-03 7.453e-03 -0.191 0.84829

PC9 8.568e-03 6.215e-03 1.378 0.16805

PC10 2.719e-04 7.516e-03 0.036 0.97114

sexMale -5.021e-01 5.819e-02 -8.629 < 2e-16 ***

age 1.355e-02 3.475e-03 3.899 9.66e-05 ***

hypertensionTRUE 3.643e-01 5.839e-02 6.240 4.37e-10 ***

hyperlipidemiaTRUE -3.965e-02 5.827e-02 -0.680 0.49621

gly_controlTRUE -3.672e-01 3.190e-01 -1.151 0.24970

obesityTRUE -1.167e-01 6.463e-02 -1.806 0.07093 .

smoking_status 4.378e-01 3.716e-02 11.781 < 2e-16 ***

---

Signif. codes: 0 ‘***’ 0.001 ‘**’ 0.01 ‘*’ 0.05 ‘.’ 0.1 ‘ ’ 1

(Dispersion parameter for binomial family taken to be 1)

Null deviance: 18812 on 443107 degrees of freedom

Residual deviance: 18536 on 443089 degrees of freedom

(45112 observations deleted due to missingness)

AIC: 18574

Number of Fisher Scoring iterations: 9

stroke.srhd.intracerebral

=========================

Call:

glm(formula = rf, family = binomial(link = "logit"), data = ms,

maxit = 100)

Deviance Residuals:

Min 1Q Median 3Q Max

-0.2552 -0.1091 -0.0865 -0.0674 3.8229

Coefficients:

Estimate Std. Error z value Pr(>|z|)

(Intercept) -9.2409952 0.1925255 -47.999 < 2e-16 ***

updated 0.0120006 0.0232002 0.517 0.604971

PC1 0.0006761 0.0004880 1.385 0.165982

PC2 -0.0012774 0.0008184 -1.561 0.118549

PC3 -0.0003767 0.0015303 -0.246 0.805539

PC4 0.0053624 0.0024708 2.170 0.029985 *

PC5 0.0068812 0.0029247 2.353 0.018633 *

PC6 -0.0057418 0.0060058 -0.956 0.339048

PC7 0.0029807 0.0053745 0.555 0.579170

PC8 -0.0029666 0.0057434 -0.517 0.605491

PC9 0.0027115 0.0051022 0.531 0.595125

PC10 -0.0044252 0.0057785 -0.766 0.443794

sexMale 0.2577738 0.0481597 5.352 8.68e-08 ***

age 0.0560320 0.0031563 17.752 < 2e-16 ***

hypertensionTRUE 0.5012520 0.0518076 9.675 < 2e-16 ***

hyperlipidemiaTRUE -0.0855229 0.0481432 -1.776 0.075662 .

gly_controlTRUE 0.4254037 0.1706456 2.493 0.012670 *

obesityTRUE -0.0245697 0.0531310 -0.462 0.643768

smoking_status 0.1291346 0.0340063 3.797 0.000146 ***

---

Signif. codes: 0 ‘***’ 0.001 ‘**’ 0.01 ‘*’ 0.05 ‘.’ 0.1 ‘ ’ 1

(Dispersion parameter for binomial family taken to be 1)

Null deviance: 25042 on 443107 degrees of freedom

Residual deviance: 24359 on 443089 degrees of freedom

(45112 observations deleted due to missingness)

AIC: 24397

Number of Fisher Scoring iterations: 8

stroke.hd.intracerebral

=========================

Call:

glm(formula = rf, family = binomial(link = "logit"), data = ms,

maxit = 100)

Deviance Residuals:

Min 1Q Median 3Q Max

-0.2584 -0.1047 -0.0816 -0.0628 3.8166

Coefficients:

Estimate Std. Error z value Pr(>|z|)

(Intercept) -9.4928989 0.2032677 -46.701 < 2e-16 ***

updated 0.0073698 0.0241491 0.305 0.7602

PC1 0.0006172 0.0005187 1.190 0.2341

PC2 -0.0011855 0.0008623 -1.375 0.1692

PC3 -0.0009598 0.0016261 -0.590 0.5550

PC4 0.0055645 0.0026424 2.106 0.0352 *

PC5 0.0065753 0.0030726 2.140 0.0324 *

PC6 -0.0068273 0.0066673 -1.024 0.3058

PC7 0.0053723 0.0056483 0.951 0.3415

PC8 -0.0019610 0.0061819 -0.317 0.7511

PC9 0.0014719 0.0053205 0.277 0.7820

PC10 -0.0083628 0.0061018 -1.371 0.1705

sexMale 0.2715232 0.0504724 5.380 7.46e-08 ***

age 0.0578440 0.0033234 17.405 < 2e-16 ***

hypertensionTRUE 0.5696743 0.0548572 10.385 < 2e-16 ***

hyperlipidemiaTRUE -0.1005686 0.0503639 -1.997 0.0458 *

gly_controlTRUE 0.4644480 0.1756841 2.644 0.0082 **

obesityTRUE -0.0569540 0.0559129 -1.019 0.3084

smoking_status 0.1393332 0.0355717 3.917 8.97e-05 ***

---

Signif. codes: 0 ‘***’ 0.001 ‘**’ 0.01 ‘*’ 0.05 ‘.’ 0.1 ‘ ’ 1

(Dispersion parameter for binomial family taken to be 1)

Null deviance: 23183 on 443107 degrees of freedom

Residual deviance: 22488 on 443089 degrees of freedom

(45112 observations deleted due to missingness)

AIC: 22526

Number of Fisher Scoring iterations: 9

stroke.srhd.ischaemic

=========================

Call:

glm(formula = rf, family = binomial(link = "logit"), data = ms,

maxit = 100)

Deviance Residuals:

Min 1Q Median 3Q Max

-0.8485 -0.2285 -0.1719 -0.1259 3.5329

Coefficients:

Estimate Std. Error z value Pr(>|z|)

(Intercept) -8.6281163 0.0950243 -90.799 < 2e-16 ***

updated 0.0063680 0.0109382 0.582 0.560446

PC1 0.0008338 0.0002221 3.754 0.000174 ***

PC2 0.0011950 0.0005197 2.299 0.021488 *

PC3 0.0013142 0.0009289 1.415 0.157111

PC4 0.0074603 0.0012282 6.074 1.25e-09 ***

PC5 0.0066474 0.0013825 4.808 1.52e-06 ***

PC6 -0.0114175 0.0043421 -2.629 0.008552 **

PC7 -0.0027850 0.0025710 -1.083 0.278707

PC8 0.0068771 0.0031801 2.163 0.030575 *

PC9 0.0145490 0.0025563 5.691 1.26e-08 ***

PC10 0.0023591 0.0028601 0.825 0.409461

sexMale 0.4286161 0.0235021 18.237 < 2e-16 ***

age 0.0640276 0.0015361 41.683 < 2e-16 ***

hypertensionTRUE 0.4980307 0.0252006 19.763 < 2e-16 ***

hyperlipidemiaTRUE 0.1487693 0.0226545 6.567 5.14e-11 ***

gly_controlTRUE 1.0281961 0.0598637 17.176 < 2e-16 ***

obesityTRUE 0.2921642 0.0236015 12.379 < 2e-16 ***

smoking_status 0.2952825 0.0158365 18.646 < 2e-16 ***

---

Signif. codes: 0 ‘***’ 0.001 ‘**’ 0.01 ‘*’ 0.05 ‘.’ 0.1 ‘ ’ 1

(Dispersion parameter for binomial family taken to be 1)

Null deviance: 86906 on 443107 degrees of freedom

Residual deviance: 81715 on 443089 degrees of freedom

(45112 observations deleted due to missingness)

AIC: 81753

Number of Fisher Scoring iterations: 7

stroke.hd.ischaemic

=========================

Call:

glm(formula = rf, family = binomial(link = "logit"), data = ms,

maxit = 100)

Deviance Residuals:

Min 1Q Median 3Q Max

-0.8462 -0.2282 -0.1719 -0.1260 3.5313

Coefficients:

Estimate Std. Error z value Pr(>|z|)

(Intercept) -8.6158632 0.0950608 -90.635 < 2e-16 ***

updated 0.0070340 0.0109574 0.642 0.520913

PC1 0.0008313 0.0002221 3.743 0.000182 ***

PC2 0.0012112 0.0005199 2.330 0.019816 *

PC3 0.0012769 0.0009293 1.374 0.169441

PC4 0.0074048 0.0012289 6.025 1.69e-09 ***

PC5 0.0065916 0.0013841 4.762 1.91e-06 ***

PC6 -0.0114398 0.0043550 -2.627 0.008619 **

PC7 -0.0028198 0.0025722 -1.096 0.272970

PC8 0.0070182 0.0031851 2.203 0.027562 *

PC9 0.0146989 0.0025604 5.741 9.42e-09 ***

PC10 0.0027138 0.0028654 0.947 0.343597

sexMale 0.4273460 0.0235211 18.169 < 2e-16 ***

age 0.0638557 0.0015370 41.546 < 2e-16 ***

hypertensionTRUE 0.4963997 0.0252138 19.688 < 2e-16 ***

hyperlipidemiaTRUE 0.1464507 0.0226794 6.457 1.06e-10 ***

gly_controlTRUE 1.0327062 0.0598640 17.251 < 2e-16 ***

obesityTRUE 0.2887591 0.0236400 12.215 < 2e-16 ***

smoking_status 0.2953648 0.0158504 18.634 < 2e-16 ***

---

Signif. codes: 0 ‘***’ 0.001 ‘**’ 0.01 ‘*’ 0.05 ‘.’ 0.1 ‘ ’ 1

(Dispersion parameter for binomial family taken to be 1)

Null deviance: 86750 on 443107 degrees of freedom

Residual deviance: 81597 on 443089 degrees of freedom

(45112 observations deleted due to missingness)

AIC: 81635

Number of Fisher Scoring iterations: 7

stroke.srhd.all

=========================

Call:

glm(formula = rf, family = binomial(link = "logit"), data = ms,

maxit = 100)

Deviance Residuals:

Min 1Q Median 3Q Max

-0.8824 -0.3033 -0.2354 -0.1765 3.2981

Coefficients:

Estimate Std. Error z value Pr(>|z|)

(Intercept) -7.5888525 0.0705866 -107.511 < 2e-16 ***

updated 0.0097586 0.0083473 1.169 0.242377

PC1 0.0006673 0.0001722 3.874 0.000107 ***

PC2 0.0007151 0.0003754 1.905 0.056822 .

PC3 0.0010663 0.0006768 1.576 0.115135

PC4 0.0076208 0.0009260 8.230 < 2e-16 ***

PC5 0.0083634 0.0010461 7.995 1.30e-15 ***

PC6 -0.0064183 0.0026567 -2.416 0.015695 *

PC7 -0.0022595 0.0019472 -1.160 0.245882

PC8 0.0025746 0.0021818 1.180 0.237978

PC9 0.0093020 0.0018878 4.927 8.33e-07 ***

PC10 0.0031047 0.0021727 1.429 0.153013

sexMale 0.2910688 0.0175802 16.557 < 2e-16 ***

age 0.0589321 0.0011504 51.227 < 2e-16 ***

hypertensionTRUE 0.4174738 0.0186711 22.359 < 2e-16 ***

hyperlipidemiaTRUE 0.1545839 0.0172303 8.972 < 2e-16 ***

gly_controlTRUE 0.8641673 0.0506352 17.067 < 2e-16 ***

obesityTRUE 0.2843876 0.0180576 15.749 < 2e-16 ***

smoking_status 0.3129503 0.0119722 26.140 < 2e-16 ***

---

Signif. codes: 0 ‘***’ 0.001 ‘**’ 0.01 ‘*’ 0.05 ‘.’ 0.1 ‘ ’ 1

(Dispersion parameter for binomial family taken to be 1)

Null deviance: 135281 on 443107 degrees of freedom

Residual deviance: 128044 on 443089 degrees of freedom

(45112 observations deleted due to missingness)

AIC: 128082

Number of Fisher Scoring iterations: 6

stroke.hd.all

=========================

Call:

glm(formula = rf, family = binomial(link = "logit"), data = ms,

maxit = 100)

Deviance Residuals:

Min 1Q Median 3Q Max

-0.8239 -0.2591 -0.2008 -0.1518 3.3845

Coefficients:

Estimate Std. Error z value Pr(>|z|)

(Intercept) -7.8033929 0.0821349 -95.007 < 2e-16 ***

updated 0.0111913 0.0097606 1.147 0.25155

PC1 0.0008027 0.0001954 4.108 3.98e-05 ***

PC2 0.0007212 0.0004248 1.698 0.08956 .

PC3 0.0007228 0.0007710 0.937 0.34853

PC4 0.0076366 0.0010887 7.015 2.31e-12 ***

PC5 0.0071943 0.0012246 5.875 4.23e-09 ***

PC6 -0.0114664 0.0037034 -3.096 0.00196 **

PC7 -0.0031400 0.0022823 -1.376 0.16888

PC8 0.0054961 0.0027646 1.988 0.04681 *

PC9 0.0126111 0.0022492 5.607 2.06e-08 ***

PC10 0.0007471 0.0025303 0.295 0.76781

sexMale 0.3084137 0.0205452 15.011 < 2e-16 ***

age 0.0570137 0.0013400 42.548 < 2e-16 ***

hypertensionTRUE 0.4848236 0.0220089 22.029 < 2e-16 ***

hyperlipidemiaTRUE 0.0967686 0.0201381 4.805 1.55e-06 ***

gly_controlTRUE 0.9251995 0.0570278 16.224 < 2e-16 ***

obesityTRUE 0.2090153 0.0212804 9.822 < 2e-16 ***

smoking_status 0.2928011 0.0139900 20.929 < 2e-16 ***

---

Signif. codes: 0 ‘***’ 0.001 ‘**’ 0.01 ‘*’ 0.05 ‘.’ 0.1 ‘ ’ 1

(Dispersion parameter for binomial family taken to be 1)

Null deviance: 105309 on 443107 degrees of freedom

Residual deviance: 100116 on 443089 degrees of freedom

(45112 observations deleted due to missingness)

AIC: 100154

Number of Fisher Scoring iterations: 7

cad.nochronic.withsr

=========================

Call:

glm(formula = rf, family = binomial(link = "logit"), data = ms,

maxit = 100)

Deviance Residuals:

Min 1Q Median 3Q Max

-1.5506 -0.4103 -0.2782 -0.1865 3.2992

Coefficients:

Estimate Std. Error z value Pr(>|z|)

(Intercept) -7.576e+00 5.223e-02 -145.035 < 2e-16 ***

updated -4.843e-05 6.071e-03 -0.008 0.993635

PC1 -1.927e-04 1.588e-04 -1.213 0.224999

PC2 -2.286e-03 2.756e-04 -8.296 < 2e-16 ***

PC3 6.161e-03 4.634e-04 13.295 < 2e-16 ***

PC4 4.864e-03 5.961e-04 8.158 3.40e-16 ***

PC5 6.772e-03 7.763e-04 8.724 < 2e-16 ***

PC6 -1.890e-03 1.698e-03 -1.113 0.265604

PC7 -2.052e-03 1.288e-03 -1.593 0.111095

PC8 4.867e-03 1.438e-03 3.384 0.000715 ***

PC9 1.075e-02 1.390e-03 7.733 1.05e-14 ***

PC10 3.835e-03 1.410e-03 2.720 0.006525 **

sexMale 9.756e-01 1.390e-02 70.196 < 2e-16 ***

age 5.760e-02 8.398e-04 68.592 < 2e-16 ***

hypertensionTRUE 4.577e-01 1.396e-02 32.784 < 2e-16 ***

hyperlipidemiaTRUE 7.837e-01 1.275e-02 61.478 < 2e-16 ***

gly_controlTRUE 9.100e-01 3.800e-02 23.949 < 2e-16 ***

obesityTRUE 3.965e-01 1.307e-02 30.340 < 2e-16 ***

smoking_status 3.309e-01 8.773e-03 37.722 < 2e-16 ***

---

Signif. codes: 0 ‘***’ 0.001 ‘**’ 0.01 ‘*’ 0.05 ‘.’ 0.1 ‘ ’ 1

(Dispersion parameter for binomial family taken to be 1)

Null deviance: 232155 on 443107 degrees of freedom

Residual deviance: 201670 on 443089 degrees of freedom

(45112 observations deleted due to missingness)

AIC: 201708

Number of Fisher Scoring iterations: 6

cad.nochronic.hd

=========================

Call:

glm(formula = rf, family = binomial(link = "logit"), data = ms,

maxit = 100)

Deviance Residuals:

Min 1Q Median 3Q Max

-1.5144 -0.3956 -0.2687 -0.1814 3.3293

Coefficients:

Estimate Std. Error z value Pr(>|z|)

(Intercept) -7.5674998 0.0538921 -140.419 < 2e-16 ***

updated -0.0032388 0.0062598 -0.517 0.60487

PC1 -0.0004473 0.0001711 -2.614 0.00894 **

PC2 -0.0024013 0.0002906 -8.264 < 2e-16 ***

PC3 0.0064335 0.0004843 13.284 < 2e-16 ***

PC4 0.0052884 0.0006212 8.513 < 2e-16 ***

PC5 0.0068692 0.0008015 8.571 < 2e-16 ***

PC6 -0.0025775 0.0017966 -1.435 0.15139

PC7 -0.0021518 0.0013476 -1.597 0.11033

PC8 0.0041384 0.0015096 2.741 0.00612 **

PC9 0.0120099 0.0014444 8.315 < 2e-16 ***

PC10 0.0042292 0.0014553 2.906 0.00366 **

sexMale 0.9641475 0.0144210 66.857 < 2e-16 ***

age 0.0560447 0.0008674 64.615 < 2e-16 ***

hypertensionTRUE 0.4734417 0.0145068 32.636 < 2e-16 ***

hyperlipidemiaTRUE 0.7727535 0.0132106 58.495 < 2e-16 ***

gly_controlTRUE 0.9146427 0.0386894 23.641 < 2e-16 ***

obesityTRUE 0.3835467 0.0135095 28.391 < 2e-16 ***

smoking_status 0.3301570 0.0090685 36.407 < 2e-16 ***

---

Signif. codes: 0 ‘***’ 0.001 ‘**’ 0.01 ‘*’ 0.05 ‘.’ 0.1 ‘ ’ 1

(Dispersion parameter for binomial family taken to be 1)

Null deviance: 219311 on 443107 degrees of freedom

Residual deviance: 191292 on 443089 degrees of freedom

(45112 observations deleted due to missingness)

AIC: 191330

Number of Fisher Scoring iterations: 6

cad.chronic.withsr

=========================

Call:

glm(formula = rf, family = binomial(link = "logit"), data = ms,

maxit = 100)

Deviance Residuals:

Min 1Q Median 3Q Max

-1.7368 -0.4995 -0.3513 -0.2331 3.1222

Coefficients:

Estimate Std. Error z value Pr(>|z|)

(Intercept) -7.2918943 0.0443739 -164.329 < 2e-16 ***

updated 0.0078243 0.0051884 1.508 0.13154

PC1 0.0002250 0.0001237 1.818 0.06900 .

PC2 -0.0021267 0.0002268 -9.379 < 2e-16 ***

PC3 0.0062457 0.0003881 16.091 < 2e-16 ***

PC4 0.0045648 0.0005047 9.045 < 2e-16 ***

PC5 0.0057231 0.0006629 8.634 < 2e-16 ***

PC6 -0.0015439 0.0013430 -1.150 0.25031

PC7 -0.0002369 0.0010944 -0.216 0.82861

PC8 0.0032457 0.0011965 2.713 0.00667 **

PC9 0.0096637 0.0011712 8.251 < 2e-16 ***

PC10 0.0062492 0.0012111 5.160 2.47e-07 ***

sexMale 0.8006436 0.0112264 71.318 < 2e-16 ***

age 0.0642712 0.0007150 89.895 < 2e-16 ***

hypertensionTRUE 0.4060405 0.0115297 35.217 < 2e-16 ***

hyperlipidemiaTRUE 0.5903594 0.0106054 55.666 < 2e-16 ***

gly_controlTRUE 0.9958344 0.0345147 28.852 < 2e-16 ***

obesityTRUE 0.5049590 0.0110803 45.573 < 2e-16 ***

smoking_status 0.3237746 0.0074753 43.313 < 2e-16 ***

---

Signif. codes: 0 ‘***’ 0.001 ‘**’ 0.01 ‘*’ 0.05 ‘.’ 0.1 ‘ ’ 1

(Dispersion parameter for binomial family taken to be 1)

Null deviance: 301057 on 443107 degrees of freedom

Residual deviance: 264279 on 443089 degrees of freedom

(45112 observations deleted due to missingness)

AIC: 264317

Number of Fisher Scoring iterations: 6

cad.chronic.hd

=========================

Call:

glm(formula = rf, family = binomial(link = "logit"), data = ms,

maxit = 100)

Deviance Residuals:

Min 1Q Median 3Q Max

-1.7287 -0.4929 -0.3464 -0.2303 3.1456

Coefficients:

Estimate Std. Error z value Pr(>|z|)

(Intercept) -7.3276885 0.0449507 -163.016 < 2e-16 ***

updated 0.0071600 0.0052462 1.365 0.1723

PC1 0.0001109 0.0001273 0.871 0.3837

PC2 -0.0021457 0.0002327 -9.222 < 2e-16 ***

PC3 0.0064553 0.0003967 16.272 < 2e-16 ***

PC4 0.0047246 0.0005116 9.235 < 2e-16 ***

PC5 0.0057845 0.0006702 8.631 < 2e-16 ***

PC6 -0.0018708 0.0013704 -1.365 0.1722

PC7 -0.0001639 0.0011134 -0.147 0.8829

PC8 0.0026587 0.0012189 2.181 0.0292 *

PC9 0.0103619 0.0011874 8.727 < 2e-16 ***

PC10 0.0064198 0.0012249 5.241 1.6e-07 ***

sexMale 0.7943442 0.0113699 69.864 < 2e-16 ***

age 0.0642467 0.0007240 88.734 < 2e-16 ***

hypertensionTRUE 0.4151029 0.0116903 35.508 < 2e-16 ***

hyperlipidemiaTRUE 0.5888949 0.0107366 54.849 < 2e-16 ***

gly_controlTRUE 1.0087299 0.0346289 29.130 < 2e-16 ***

obesityTRUE 0.5045736 0.0112002 45.050 < 2e-16 ***

smoking_status 0.3236200 0.0075646 42.781 < 2e-16 ***

---

Signif. codes: 0 ‘***’ 0.001 ‘**’ 0.01 ‘*’ 0.05 ‘.’ 0.1 ‘ ’ 1

(Dispersion parameter for binomial family taken to be 1)

Null deviance: 295394 on 443107 degrees of freedom

Residual deviance: 259385 on 443089 degrees of freedom

(45112 observations deleted due to missingness)

AIC: 259423

Number of Fisher Scoring iterations: 6

### Updated score, serum only

stroke.srhd.subarachnoid

=========================

Call:

glm(formula = rf, family = binomial(link = "logit"), data = ms,

maxit = 100)

Deviance Residuals:

Min 1Q Median 3Q Max

-0.1802 -0.0943 -0.0815 -0.0708 3.8039

Coefficients:

Estimate Std. Error z value Pr(>|z|)

(Intercept) -6.7757168 0.1888995 -35.869 < 2e-16 ***

updated.serum 0.0017813 0.0250394 0.071 0.943286

PC1 -0.0002324 0.0005438 -0.427 0.669101

PC2 0.0009075 0.0010848 0.837 0.402866

PC3 -0.0002158 0.0020302 -0.106 0.915367

PC4 0.0099116 0.0029933 3.311 0.000929 ***

PC5 0.0059668 0.0031637 1.886 0.059295 .

PC6 -0.0134749 0.0089120 -1.512 0.130534

PC7 -0.0129252 0.0063403 -2.039 0.041493 *

PC8 -0.0044189 0.0069970 -0.632 0.527683

PC9 0.0006588 0.0055485 0.119 0.905488

PC10 0.0007973 0.0070474 0.113 0.909926

sexMale -0.4434038 0.0536501 -8.265 < 2e-16 ***

age 0.0157236 0.0032334 4.863 1.16e-06 ***

hypertensionTRUE 0.3673256 0.0542685 6.769 1.30e-11 ***

hyperlipidemiaTRUE -0.0460443 0.0539608 -0.853 0.393498

gly_controlTRUE -0.4426047 0.3040716 -1.456 0.145505

obesityTRUE -0.0790395 0.0593826 -1.331 0.183182

smoking_status 0.4098294 0.0347024 11.810 < 2e-16 ***

---

Signif. codes: 0 ‘***’ 0.001 ‘**’ 0.01 ‘*’ 0.05 ‘.’ 0.1 ‘ ’ 1

(Dispersion parameter for binomial family taken to be 1)

Null deviance: 21381 on 443107 degrees of freedom

Residual deviance: 21091 on 443089 degrees of freedom

(45112 observations deleted due to missingness)

AIC: 21129

Number of Fisher Scoring iterations: 9

stroke.hd.subarachnoid

=========================

Call:

glm(formula = rf, family = binomial(link = "logit"), data = ms,

maxit = 100)

Deviance Residuals:

Min 1Q Median 3Q Max

-0.1786 -0.0871 -0.0754 -0.0649 3.8136

Coefficients:

Estimate Std. Error z value Pr(>|z|)

(Intercept) -6.791e+00 2.026e-01 -33.524 < 2e-16 ***

updated.serum 1.075e-02 2.737e-02 0.393 0.69451

PC1 6.555e-05 5.597e-04 0.117 0.90677

PC2 6.994e-04 1.109e-03 0.631 0.52828

PC3 -5.577e-04 2.098e-03 -0.266 0.79041

PC4 9.343e-03 3.158e-03 2.959 0.00309 **

PC5 8.676e-03 3.361e-03 2.581 0.00984 **

PC6 -1.326e-02 9.567e-03 -1.386 0.16585

PC7 -1.154e-02 6.651e-03 -1.735 0.08267 .

PC8 -1.432e-03 7.454e-03 -0.192 0.84769

PC9 8.575e-03 6.215e-03 1.380 0.16766

PC10 2.701e-04 7.516e-03 0.036 0.97133

sexMale -5.021e-01 5.819e-02 -8.629 < 2e-16 ***

age 1.355e-02 3.475e-03 3.899 9.65e-05 ***

hypertensionTRUE 3.643e-01 5.839e-02 6.240 4.38e-10 ***

hyperlipidemiaTRUE -3.966e-02 5.826e-02 -0.681 0.49607

gly_controlTRUE -3.672e-01 3.190e-01 -1.151 0.24975

obesityTRUE -1.167e-01 6.463e-02 -1.805 0.07104 .

smoking_status 4.378e-01 3.716e-02 11.782 < 2e-16 ***

---

Signif. codes: 0 ‘***’ 0.001 ‘**’ 0.01 ‘*’ 0.05 ‘.’ 0.1 ‘ ’ 1

(Dispersion parameter for binomial family taken to be 1)

Null deviance: 18812 on 443107 degrees of freedom

Residual deviance: 18536 on 443089 degrees of freedom

(45112 observations deleted due to missingness)

AIC: 18574

Number of Fisher Scoring iterations: 9

stroke.srhd.intracerebral

=========================

Call:

glm(formula = rf, family = binomial(link = "logit"), data = ms,

maxit = 100)

Deviance Residuals:

Min 1Q Median 3Q Max

-0.2548 -0.1091 -0.0865 -0.0674 3.8214

Coefficients:

Estimate Std. Error z value Pr(>|z|)

(Intercept) -9.2413211 0.1925296 -47.999 < 2e-16 ***

updated.serum 0.0214119 0.0235604 0.909 0.363451

PC1 0.0006563 0.0004872 1.347 0.177963

PC2 -0.0012755 0.0008183 -1.559 0.119086

PC3 -0.0003906 0.0015301 -0.255 0.798500

PC4 0.0053425 0.0024702 2.163 0.030558 *

PC5 0.0068882 0.0029248 2.355 0.018517 *

PC6 -0.0057389 0.0060087 -0.955 0.339527

PC7 0.0029518 0.0053739 0.549 0.582805

PC8 -0.0029924 0.0057442 -0.521 0.602412

PC9 0.0027354 0.0051019 0.536 0.591850

PC10 -0.0044335 0.0057776 -0.767 0.442860

sexMale 0.2577702 0.0481597 5.352 8.68e-08 ***

age 0.0560344 0.0031564 17.753 < 2e-16 ***

hypertensionTRUE 0.5012263 0.0518079 9.675 < 2e-16 ***

hyperlipidemiaTRUE -0.0855099 0.0481431 -1.776 0.075706 .

gly_controlTRUE 0.4256592 0.1706469 2.494 0.012618 *

obesityTRUE -0.0245251 0.0531309 -0.462 0.644370

smoking_status 0.1291532 0.0340060 3.798 0.000146 ***

---

Signif. codes: 0 ‘***’ 0.001 ‘**’ 0.01 ‘*’ 0.05 ‘.’ 0.1 ‘ ’ 1

(Dispersion parameter for binomial family taken to be 1)

Null deviance: 25042 on 443107 degrees of freedom

Residual deviance: 24359 on 443089 degrees of freedom

(45112 observations deleted due to missingness)

AIC: 24397

Number of Fisher Scoring iterations: 8

stroke.hd.intracerebral

=========================

Call:

glm(formula = rf, family = binomial(link = "logit"), data = ms,

maxit = 100)

Deviance Residuals:

Min 1Q Median 3Q Max

-0.2585 -0.1048 -0.0816 -0.0627 3.8165

Coefficients:

Estimate Std. Error z value Pr(>|z|)

(Intercept) -9.4932373 0.2032722 -46.702 < 2e-16 ***

updated.serum 0.0181866 0.0245328 0.741 0.45850

PC1 0.0005923 0.0005179 1.144 0.25269

PC2 -0.0011850 0.0008623 -1.374 0.16935

PC3 -0.0009771 0.0016260 -0.601 0.54789

PC4 0.0055547 0.0026417 2.103 0.03549 *

PC5 0.0065810 0.0030727 2.142 0.03221 *

PC6 -0.0068268 0.0066704 -1.023 0.30610

PC7 0.0053383 0.0056476 0.945 0.34454

PC8 -0.0019852 0.0061828 -0.321 0.74815

PC9 0.0014949 0.0053201 0.281 0.77872

PC10 -0.0083694 0.0061008 -1.372 0.17011

sexMale 0.2715312 0.0504724 5.380 7.46e-08 ***

age 0.0578468 0.0033234 17.406 < 2e-16 ***

hypertensionTRUE 0.5696550 0.0548574 10.384 < 2e-16 ***

hyperlipidemiaTRUE -0.1005490 0.0503638 -1.996 0.04588 *

gly_controlTRUE 0.4646646 0.1756856 2.645 0.00817 **

obesityTRUE -0.0569288 0.0559128 -1.018 0.30860

smoking_status 0.1393408 0.0355714 3.917 8.96e-05 ***

---

Signif. codes: 0 ‘***’ 0.001 ‘**’ 0.01 ‘*’ 0.05 ‘.’ 0.1 ‘ ’ 1

(Dispersion parameter for binomial family taken to be 1)

Null deviance: 23183 on 443107 degrees of freedom

Residual deviance: 22487 on 443089 degrees of freedom

(45112 observations deleted due to missingness)

AIC: 22525

Number of Fisher Scoring iterations: 9

stroke.srhd.ischaemic

=========================

Call:

glm(formula = rf, family = binomial(link = "logit"), data = ms,

maxit = 100)

Deviance Residuals:

Min 1Q Median 3Q Max

-0.8482 -0.2285 -0.1719 -0.1259 3.5318

Coefficients:

Estimate Std. Error z value Pr(>|z|)

(Intercept) -8.6280451 0.0950242 -90.798 < 2e-16 ***

updated.serum 0.0032276 0.0108817 0.297 0.766766

PC1 0.0008436 0.0002216 3.807 0.000141 ***

PC2 0.0011971 0.0005197 2.303 0.021254 *

PC3 0.0013209 0.0009289 1.422 0.155003

PC4 0.0074468 0.0012280 6.064 1.33e-09 ***

PC5 0.0066482 0.0013825 4.809 1.52e-06 ***

PC6 -0.0114178 0.0043422 -2.629 0.008552 **

PC7 -0.0027759 0.0025710 -1.080 0.280276

PC8 0.0068783 0.0031801 2.163 0.030547 *

PC9 0.0145477 0.0025563 5.691 1.26e-08 ***

PC10 0.0023598 0.0028601 0.825 0.409318

sexMale 0.4285950 0.0235021 18.236 < 2e-16 ***

age 0.0640269 0.0015361 41.682 < 2e-16 ***

hypertensionTRUE 0.4980206 0.0252007 19.762 < 2e-16 ***

hyperlipidemiaTRUE 0.1487572 0.0226545 6.566 5.16e-11 ***

gly_controlTRUE 1.0282096 0.0598636 17.176 < 2e-16 ***

obesityTRUE 0.2921960 0.0236014 12.380 < 2e-16 ***

smoking_status 0.2952978 0.0158365 18.647 < 2e-16 ***

---

Signif. codes: 0 ‘***’ 0.001 ‘**’ 0.01 ‘*’ 0.05 ‘.’ 0.1 ‘ ’ 1

(Dispersion parameter for binomial family taken to be 1)

Null deviance: 86906 on 443107 degrees of freedom

Residual deviance: 81716 on 443089 degrees of freedom

(45112 observations deleted due to missingness)

AIC: 81754

Number of Fisher Scoring iterations: 7

stroke.hd.ischaemic

=========================

Call:

glm(formula = rf, family = binomial(link = "logit"), data = ms,

maxit = 100)

Deviance Residuals:

Min 1Q Median 3Q Max

-0.8459 -0.2282 -0.1719 -0.1260 3.5303

Coefficients:

Estimate Std. Error z value Pr(>|z|)

(Intercept) -8.6157929 0.0950607 -90.635 < 2e-16 ***

updated.serum 0.0039626 0.0109047 0.363 0.716315

PC1 0.0008412 0.0002217 3.795 0.000148 ***

PC2 0.0012135 0.0005199 2.334 0.019580 *

PC3 0.0012836 0.0009293 1.381 0.167213

PC4 0.0073900 0.0012287 6.015 1.80e-09 ***

PC5 0.0065927 0.0013841 4.763 1.91e-06 ***

PC6 -0.0114403 0.0043552 -2.627 0.008619 **

PC7 -0.0028110 0.0025723 -1.093 0.274468

PC8 0.0070189 0.0031851 2.204 0.027548 *

PC9 0.0146981 0.0025605 5.740 9.45e-09 ***

PC10 0.0027143 0.0028654 0.947 0.343505

sexMale 0.4273238 0.0235211 18.168 < 2e-16 ***

age 0.0638550 0.0015370 41.545 < 2e-16 ***

hypertensionTRUE 0.4963879 0.0252139 19.687 < 2e-16 ***

hyperlipidemiaTRUE 0.1464381 0.0226794 6.457 1.07e-10 ***

gly_controlTRUE 1.0327268 0.0598639 17.251 < 2e-16 ***

obesityTRUE 0.2887941 0.0236399 12.216 < 2e-16 ***

smoking_status 0.2953814 0.0158504 18.636 < 2e-16 ***

---

Signif. codes: 0 ‘***’ 0.001 ‘**’ 0.01 ‘*’ 0.05 ‘.’ 0.1 ‘ ’ 1

(Dispersion parameter for binomial family taken to be 1)

Null deviance: 86750 on 443107 degrees of freedom

Residual deviance: 81598 on 443089 degrees of freedom

(45112 observations deleted due to missingness)

AIC: 81636

Number of Fisher Scoring iterations: 7

stroke.srhd.all

=========================

Call:

glm(formula = rf, family = binomial(link = "logit"), data = ms,

maxit = 100)

Deviance Residuals:

Min 1Q Median 3Q Max

-0.8811 -0.3033 -0.2355 -0.1765 3.2986

Coefficients:

Estimate Std. Error z value Pr(>|z|)

(Intercept) -7.5888243 0.0705868 -107.511 < 2e-16 ***

updated.serum 0.0081032 0.0083315 0.973 0.3308

PC1 0.0006745 0.0001719 3.923 8.73e-05 ***

PC2 0.0007180 0.0003754 1.912 0.0558 .

PC3 0.0010710 0.0006767 1.583 0.1135

PC4 0.0076011 0.0009258 8.211 < 2e-16 ***

PC5 0.0083660 0.0010461 7.997 1.27e-15 ***

PC6 -0.0064183 0.0026571 -2.416 0.0157 *

PC7 -0.0022554 0.0019472 -1.158 0.2467

PC8 0.0025707 0.0021819 1.178 0.2387

PC9 0.0093056 0.0018878 4.929 8.26e-07 ***

PC10 0.0031033 0.0021727 1.428 0.1532

sexMale 0.2910450 0.0175802 16.555 < 2e-16 ***

age 0.0589320 0.0011504 51.227 < 2e-16 ***

hypertensionTRUE 0.4174555 0.0186712 22.358 < 2e-16 ***

hyperlipidemiaTRUE 0.1545734 0.0172303 8.971 < 2e-16 ***

gly_controlTRUE 0.8642293 0.0506352 17.068 < 2e-16 ***

obesityTRUE 0.2844332 0.0180575 15.752 < 2e-16 ***

smoking_status 0.3129710 0.0119722 26.141 < 2e-16 ***

---

Signif. codes: 0 ‘***’ 0.001 ‘**’ 0.01 ‘*’ 0.05 ‘.’ 0.1 ‘ ’ 1

(Dispersion parameter for binomial family taken to be 1)

Null deviance: 135281 on 443107 degrees of freedom

Residual deviance: 128044 on 443089 degrees of freedom

(45112 observations deleted due to missingness)

AIC: 128082

Number of Fisher Scoring iterations: 6

stroke.hd.all

=========================

Call:

glm(formula = rf, family = binomial(link = "logit"), data = ms,

maxit = 100)

Deviance Residuals:

Min 1Q Median 3Q Max

-0.8237 -0.2591 -0.2008 -0.1518 3.3856

Coefficients:

Estimate Std. Error z value Pr(>|z|)

(Intercept) -7.8033770 0.0821352 -95.007 < 2e-16 ***

updated.serum 0.0100404 0.0097549 1.029 0.30335

PC1 0.0008091 0.0001950 4.149 3.33e-05 ***

PC2 0.0007245 0.0004248 1.706 0.08810 .

PC3 0.0007269 0.0007709 0.943 0.34578

PC4 0.0076144 0.0010884 6.996 2.64e-12 ***

PC5 0.0071975 0.0012246 5.878 4.16e-09 ***

PC6 -0.0114687 0.0037042 -3.096 0.00196 **

PC7 -0.0031376 0.0022823 -1.375 0.16921

PC8 0.0054915 0.0027649 1.986 0.04701 *

PC9 0.0126162 0.0022492 5.609 2.03e-08 ***

PC10 0.0007454 0.0025303 0.295 0.76830

sexMale 0.3083894 0.0205452 15.010 < 2e-16 ***

age 0.0570138 0.0013400 42.548 < 2e-16 ***

hypertensionTRUE 0.4848021 0.0220090 22.027 < 2e-16 ***

hyperlipidemiaTRUE 0.0967565 0.0201381 4.805 1.55e-06 ***

gly_controlTRUE 0.9252823 0.0570278 16.225 < 2e-16 ***

obesityTRUE 0.2090671 0.0212804 9.824 < 2e-16 ***

smoking_status 0.2928247 0.0139899 20.931 < 2e-16 ***

---

Signif. codes: 0 ‘***’ 0.001 ‘**’ 0.01 ‘*’ 0.05 ‘.’ 0.1 ‘ ’ 1

(Dispersion parameter for binomial family taken to be 1)

Null deviance: 105309 on 443107 degrees of freedom

Residual deviance: 100116 on 443089 degrees of freedom

(45112 observations deleted due to missingness)

AIC: 100154

Number of Fisher Scoring iterations: 7

cad.nochronic.withsr

=========================

Call:

glm(formula = rf, family = binomial(link = "logit"), data = ms,

maxit = 100)

Deviance Residuals:

Min 1Q Median 3Q Max

-1.5508 -0.4102 -0.2782 -0.1865 3.2992

Coefficients:

Estimate Std. Error z value Pr(>|z|)

(Intercept) -7.5755619 0.0522326 -145.035 < 2e-16 ***

updated.serum -0.0012421 0.0060457 -0.205 0.837225

PC1 -0.0001897 0.0001586 -1.196 0.231748

PC2 -0.0022861 0.0002756 -8.296 < 2e-16 ***

PC3 0.0061628 0.0004633 13.301 < 2e-16 ***

PC4 0.0048632 0.0005960 8.160 3.35e-16 ***

PC5 0.0067721 0.0007763 8.724 < 2e-16 ***

PC6 -0.0018902 0.0016980 -1.113 0.265631

PC7 -0.0020484 0.0012878 -1.591 0.111698

PC8 0.0048691 0.0014384 3.385 0.000711 ***

PC9 0.0107493 0.0013904 7.731 1.06e-14 ***

PC10 0.0038359 0.0014099 2.721 0.006513 **

sexMale 0.9756114 0.0138984 70.196 < 2e-16 ***

age 0.0576000 0.0008398 68.591 < 2e-16 ***

hypertensionTRUE 0.4576761 0.0139604 32.784 < 2e-16 ***

hyperlipidemiaTRUE 0.7836901 0.0127475 61.478 < 2e-16 ***

gly_controlTRUE 0.9100088 0.0379992 23.948 < 2e-16 ***

obesityTRUE 0.3964816 0.0130677 30.340 < 2e-16 ***

smoking_status 0.3309330 0.0087729 37.722 < 2e-16 ***

---

Signif. codes: 0 ‘***’ 0.001 ‘**’ 0.01 ‘*’ 0.05 ‘.’ 0.1 ‘ ’ 1

(Dispersion parameter for binomial family taken to be 1)

Null deviance: 232155 on 443107 degrees of freedom

Residual deviance: 201670 on 443089 degrees of freedom

(45112 observations deleted due to missingness)

AIC: 201708

Number of Fisher Scoring iterations: 6

cad.nochronic.hd

=========================

Call:

glm(formula = rf, family = binomial(link = "logit"), data = ms,

maxit = 100)

Deviance Residuals:

Min 1Q Median 3Q Max

-1.5144 -0.3956 -0.2687 -0.1814 3.3290

Coefficients:

Estimate Std. Error z value Pr(>|z|)

(Intercept) -7.5674895 0.0538920 -140.419 < 2e-16 ***

updated.serum -0.0040143 0.0062308 -0.644 0.51940

PC1 -0.0004464 0.0001709 -2.612 0.00901 **

PC2 -0.0024021 0.0002906 -8.267 < 2e-16 ***

PC3 0.0064344 0.0004843 13.286 < 2e-16 ***

PC4 0.0052945 0.0006210 8.525 < 2e-16 ***

PC5 0.0068681 0.0008015 8.569 < 2e-16 ***

PC6 -0.0025772 0.0017965 -1.435 0.15141

PC7 -0.0021494 0.0013477 -1.595 0.11073

PC8 0.0041422 0.0015096 2.744 0.00607 **

PC9 0.0120071 0.0014445 8.312 < 2e-16 ***

PC10 0.0042311 0.0014553 2.907 0.00364 **

sexMale 0.9641509 0.0144210 66.858 < 2e-16 ***

age 0.0560444 0.0008674 64.615 < 2e-16 ***

hypertensionTRUE 0.4734524 0.0145068 32.637 < 2e-16 ***

hyperlipidemiaTRUE 0.7727532 0.0132106 58.495 < 2e-16 ***

gly_controlTRUE 0.9145980 0.0386895 23.639 < 2e-16 ***

obesityTRUE 0.3835334 0.0135094 28.390 < 2e-16 ***

smoking_status 0.3301555 0.0090685 36.407 < 2e-16 ***

---

Signif. codes: 0 ‘***’ 0.001 ‘**’ 0.01 ‘*’ 0.05 ‘.’ 0.1 ‘ ’ 1

(Dispersion parameter for binomial family taken to be 1)

Null deviance: 219311 on 443107 degrees of freedom

Residual deviance: 191292 on 443089 degrees of freedom

(45112 observations deleted due to missingness)

AIC: 191330

Number of Fisher Scoring iterations: 6

cad.chronic.withsr

=========================

Call:

glm(formula = rf, family = binomial(link = "logit"), data = ms,

maxit = 100)

Deviance Residuals:

Min 1Q Median 3Q Max

-1.7356 -0.4995 -0.3513 -0.2331 3.1226

Coefficients:

Estimate Std. Error z value Pr(>|z|)

(Intercept) -7.2917501 0.0443736 -164.326 < 2e-16 ***

updated.serum 0.0036749 0.0051628 0.712 0.47659

PC1 0.0002379 0.0001235 1.926 0.05414 .

PC2 -0.0021241 0.0002267 -9.368 < 2e-16 ***

PC3 0.0062545 0.0003881 16.115 < 2e-16 ***

PC4 0.0045481 0.0005046 9.014 < 2e-16 ***

PC5 0.0057241 0.0006629 8.635 < 2e-16 ***

PC6 -0.0015435 0.0013431 -1.149 0.25045

PC7 -0.0002256 0.0010945 -0.206 0.83670

PC8 0.0032469 0.0011965 2.714 0.00665 **

PC9 0.0096611 0.0011712 8.249 < 2e-16 ***

PC10 0.0062489 0.0012111 5.160 2.47e-07 ***

sexMale 0.8006161 0.0112263 71.316 < 2e-16 ***

age 0.0642694 0.0007150 89.893 < 2e-16 ***

hypertensionTRUE 0.4060306 0.0115297 35.216 < 2e-16 ***

hyperlipidemiaTRUE 0.5903491 0.0106054 55.665 < 2e-16 ***

gly_controlTRUE 0.9958189 0.0345143 28.852 < 2e-16 ***

obesityTRUE 0.5049921 0.0110803 45.576 < 2e-16 ***

smoking_status 0.3237918 0.0074753 43.315 < 2e-16 ***

---

Signif. codes: 0 ‘***’ 0.001 ‘**’ 0.01 ‘*’ 0.05 ‘.’ 0.1 ‘ ’ 1

(Dispersion parameter for binomial family taken to be 1)

Null deviance: 301057 on 443107 degrees of freedom

Residual deviance: 264281 on 443089 degrees of freedom

(45112 observations deleted due to missingness)

AIC: 264319

Number of Fisher Scoring iterations: 6

cad.chronic.hd

=========================

Call:

glm(formula = rf, family = binomial(link = "logit"), data = ms,

maxit = 100)

Deviance Residuals:

Min 1Q Median 3Q Max

-1.7275 -0.4929 -0.3465 -0.2302 3.1459

Coefficients:

Estimate Std. Error z value Pr(>|z|)

(Intercept) -7.3275502 0.0449503 -163.014 < 2e-16 ***

updated.serum 0.0030629 0.0052196 0.587 0.5573

PC1 0.0001234 0.0001271 0.971 0.3314

PC2 -0.0021433 0.0002327 -9.212 < 2e-16 ***

PC3 0.0064639 0.0003967 16.295 < 2e-16 ***

PC4 0.0047093 0.0005115 9.207 < 2e-16 ***

PC5 0.0057853 0.0006702 8.632 < 2e-16 ***

PC6 -0.0018704 0.0013705 -1.365 0.1723

PC7 -0.0001527 0.0011134 -0.137 0.8909

PC8 0.0026604 0.0012190 2.183 0.0291 *

PC9 0.0103591 0.0011874 8.724 < 2e-16 ***

PC10 0.0064197 0.0012250 5.241 1.6e-07 ***

sexMale 0.7943184 0.0113698 69.862 < 2e-16 ***

age 0.0642450 0.0007240 88.732 < 2e-16 ***

hypertensionTRUE 0.4150944 0.0116903 35.508 < 2e-16 ***

hyperlipidemiaTRUE 0.5888852 0.0107365 54.849 < 2e-16 ***

gly_controlTRUE 1.0087107 0.0346286 29.129 < 2e-16 ***

obesityTRUE 0.5046043 0.0112002 45.053 < 2e-16 ***

smoking_status 0.3236364 0.0075646 42.783 < 2e-16 ***

---

Signif. codes: 0 ‘***’ 0.001 ‘**’ 0.01 ‘*’ 0.05 ‘.’ 0.1 ‘ ’ 1

(Dispersion parameter for binomial family taken to be 1)

Null deviance: 295394 on 443107 degrees of freedom

Residual deviance: 259386 on 443089 degrees of freedom

(45112 observations deleted due to missingness)

AIC: 259424

Number of Fisher Scoring iterations: 6

### MPO burden score only

stroke.srhd.subarachnoid

=========================

Call:

glm(formula = rf, family = binomial(link = "logit"), data = ms,

maxit = 100)

Deviance Residuals:

Min 1Q Median 3Q Max

-0.1806 -0.0943 -0.0815 -0.0708 3.8040

Coefficients:

Estimate Std. Error z value Pr(>|z|)

(Intercept) -6.7717472 0.1890047 -35.828 < 2e-16 ***

rvMPO -0.0568047 0.0940548 -0.604 0.54587

PC1 -0.0002416 0.0005405 -0.447 0.65489

PC2 0.0009228 0.0010851 0.850 0.39510

PC3 -0.0002302 0.0020299 -0.113 0.90971

PC4 0.0099191 0.0029932 3.314 0.00092 ***

PC5 0.0059273 0.0031642 1.873 0.06104 .

PC6 -0.0134953 0.0089119 -1.514 0.12995

PC7 -0.0129398 0.0063399 -2.041 0.04125 *

PC8 -0.0044458 0.0069969 -0.635 0.52517

PC9 0.0006385 0.0055483 0.115 0.90839

PC10 0.0008023 0.0070477 0.114 0.90937

sexMale -0.4434896 0.0536507 -8.266 < 2e-16 ***

age 0.0157275 0.0032334 4.864 1.15e-06 ***

hypertensionTRUE 0.3673943 0.0542690 6.770 1.29e-11 ***

hyperlipidemiaTRUE -0.0460544 0.0539612 -0.853 0.39340

gly_controlTRUE -0.4428014 0.3040719 -1.456 0.14533

obesityTRUE -0.0790609 0.0593825 -1.331 0.18306

smoking_status 0.4098189 0.0347029 11.809 < 2e-16 ***

---

Signif. codes: 0 ‘***’ 0.001 ‘**’ 0.01 ‘*’ 0.05 ‘.’ 0.1 ‘ ’ 1

(Dispersion parameter for binomial family taken to be 1)

Null deviance: 21381 on 443107 degrees of freedom

Residual deviance: 21091 on 443089 degrees of freedom

(45112 observations deleted due to missingness)

AIC: 21129

Number of Fisher Scoring iterations: 9

stroke.hd.subarachnoid

=========================

Call:

glm(formula = rf, family = binomial(link = "logit"), data = ms,

maxit = 100)

Deviance Residuals:

Min 1Q Median 3Q Max

-0.1794 -0.0871 -0.0754 -0.0649 3.8136

Coefficients:

Estimate Std. Error z value Pr(>|z|)

(Intercept) -6.788e+00 2.027e-01 -33.491 < 2e-16 ***

rvMPO -3.632e-02 1.007e-01 -0.361 0.71827

PC1 8.384e-05 5.560e-04 0.151 0.88014

PC2 7.103e-04 1.109e-03 0.640 0.52203

PC3 -5.504e-04 2.098e-03 -0.262 0.79307

PC4 9.344e-03 3.158e-03 2.959 0.00309 **

PC5 8.646e-03 3.361e-03 2.572 0.01010 *

PC6 -1.327e-02 9.564e-03 -1.387 0.16540

PC7 -1.152e-02 6.651e-03 -1.733 0.08316 .

PC8 -1.436e-03 7.453e-03 -0.193 0.84715

PC9 8.548e-03 6.216e-03 1.375 0.16904

PC10 2.792e-04 7.517e-03 0.037 0.97036

sexMale -5.022e-01 5.819e-02 -8.630 < 2e-16 ***

age 1.355e-02 3.475e-03 3.900 9.63e-05 ***

hypertensionTRUE 3.644e-01 5.839e-02 6.241 4.36e-10 ***

hyperlipidemiaTRUE -3.966e-02 5.827e-02 -0.681 0.49605

gly_controlTRUE -3.675e-01 3.190e-01 -1.152 0.24935

obesityTRUE -1.167e-01 6.463e-02 -1.805 0.07101 .

smoking_status 4.378e-01 3.716e-02 11.781 < 2e-16 ***

---

Signif. codes: 0 ‘***’ 0.001 ‘**’ 0.01 ‘*’ 0.05 ‘.’ 0.1 ‘ ’ 1

(Dispersion parameter for binomial family taken to be 1)

Null deviance: 18812 on 443107 degrees of freedom

Residual deviance: 18536 on 443089 degrees of freedom

(45112 observations deleted due to missingness)

AIC: 18574

Number of Fisher Scoring iterations: 9

stroke.srhd.intracerebral

=========================

Call:

glm(formula = rf, family = binomial(link = "logit"), data = ms,

maxit = 100)

Deviance Residuals:

Min 1Q Median 3Q Max

-0.2539 -0.1091 -0.0865 -0.0674 3.8233

Coefficients:

Estimate Std. Error z value Pr(>|z|)

(Intercept) -9.2355495 0.1926125 -47.949 < 2e-16 ***

rvMPO -0.0726653 0.0866705 -0.838 0.401801

PC1 0.0006928 0.0004840 1.431 0.152362

PC2 -0.0012534 0.0008186 -1.531 0.125757

PC3 -0.0003757 0.0015299 -0.246 0.806016

PC4 0.0053419 0.0024702 2.163 0.030575 *

PC5 0.0068311 0.0029251 2.335 0.019525 *

PC6 -0.0057601 0.0060041 -0.959 0.337381

PC7 0.0029894 0.0053744 0.556 0.578049

PC8 -0.0029954 0.0057428 -0.522 0.601951

PC9 0.0026779 0.0051026 0.525 0.599708

PC10 -0.0044158 0.0057792 -0.764 0.444815

sexMale 0.2576260 0.0481604 5.349 8.83e-08 ***

age 0.0560327 0.0031563 17.753 < 2e-16 ***

hypertensionTRUE 0.5012946 0.0518085 9.676 < 2e-16 ***

hyperlipidemiaTRUE -0.0855812 0.0481441 -1.778 0.075468 .

gly_controlTRUE 0.4250613 0.1706459 2.491 0.012742 *

obesityTRUE -0.0245488 0.0531308 -0.462 0.644049

smoking_status 0.1291448 0.0340075 3.798 0.000146 ***

---

Signif. codes: 0 ‘***’ 0.001 ‘**’ 0.01 ‘*’ 0.05 ‘.’ 0.1 ‘ ’ 1

(Dispersion parameter for binomial family taken to be 1)

Null deviance: 25042 on 443107 degrees of freedom

Residual deviance: 24359 on 443089 degrees of freedom

(45112 observations deleted due to missingness)

AIC: 24397

Number of Fisher Scoring iterations: 8

stroke.hd.intracerebral

=========================

Call:

glm(formula = rf, family = binomial(link = "logit"), data = ms,

maxit = 100)

Deviance Residuals:

Min 1Q Median 3Q Max

-0.2580 -0.1047 -0.0816 -0.0627 3.8139

Coefficients:

Estimate Std. Error z value Pr(>|z|)

(Intercept) -9.4852520 0.2033546 -46.644 < 2e-16 ***

rvMPO -0.1064909 0.0920694 -1.157 0.24742

PC1 0.0006132 0.0005146 1.192 0.23343

PC2 -0.0011546 0.0008626 -1.339 0.18071

PC3 -0.0009777 0.0016258 -0.601 0.54760

PC4 0.0055582 0.0026416 2.104 0.03537 *

PC5 0.0065026 0.0030729 2.116 0.03434 *

PC6 -0.0068549 0.0066649 -1.029 0.30371

PC7 0.0053562 0.0056481 0.948 0.34296

PC8 -0.0020144 0.0061811 -0.326 0.74451

PC9 0.0014334 0.0053207 0.269 0.78762

PC10 -0.0083545 0.0061026 -1.369 0.17100

sexMale 0.2713425 0.0504734 5.376 7.62e-08 ***

age 0.0578466 0.0033233 17.406 < 2e-16 ***

hypertensionTRUE 0.5697518 0.0548583 10.386 < 2e-16 ***

hyperlipidemiaTRUE -0.1006277 0.0503652 -1.998 0.04572 *

gly_controlTRUE 0.4640157 0.1756854 2.641 0.00826 **

obesityTRUE -0.0569696 0.0559127 -1.019 0.30825

smoking_status 0.1393187 0.0355733 3.916 8.99e-05 ***

---

Signif. codes: 0 ‘***’ 0.001 ‘**’ 0.01 ‘*’ 0.05 ‘.’ 0.1 ‘ ’ 1

(Dispersion parameter for binomial family taken to be 1)

Null deviance: 23183 on 443107 degrees of freedom

Residual deviance: 22486 on 443089 degrees of freedom

(45112 observations deleted due to missingness)

AIC: 22524

Number of Fisher Scoring iterations: 9

stroke.srhd.ischaemic

=========================

Call:

glm(formula = rf, family = binomial(link = "logit"), data = ms,

maxit = 100)

Deviance Residuals:

Min 1Q Median 3Q Max

-0.8479 -0.2285 -0.1719 -0.1259 3.5285

Coefficients:

Estimate Std. Error z value Pr(>|z|)

(Intercept) -8.6282720 0.0950714 -90.756 < 2e-16 ***

rvMPO 0.0039693 0.0398166 0.100 0.920590

PC1 0.0008528 0.0002201 3.874 0.000107 ***

PC2 0.0011963 0.0005198 2.301 0.021369 *

PC3 0.0013279 0.0009288 1.430 0.152785

PC4 0.0074452 0.0012280 6.063 1.34e-09 ***

PC5 0.0066500 0.0013828 4.809 1.52e-06 ***

PC6 -0.0114147 0.0043418 -2.629 0.008564 **

PC7 -0.0027646 0.0025709 -1.075 0.282230

PC8 0.0068857 0.0031800 2.165 0.030364 *

PC9 0.0145438 0.0025564 5.689 1.28e-08 ***

PC10 0.0023620 0.0028601 0.826 0.408904

sexMale 0.4285918 0.0235021 18.236 < 2e-16 ***

age 0.0640259 0.0015361 41.682 < 2e-16 ***

hypertensionTRUE 0.4980240 0.0252006 19.762 < 2e-16 ***

hyperlipidemiaTRUE 0.1487537 0.0226545 6.566 5.16e-11 ***

gly_controlTRUE 1.0281802 0.0598633 17.175 < 2e-16 ***

obesityTRUE 0.2922005 0.0236014 12.381 < 2e-16 ***

smoking_status 0.2953019 0.0158364 18.647 < 2e-16 ***

---

Signif. codes: 0 ‘***’ 0.001 ‘**’ 0.01 ‘*’ 0.05 ‘.’ 0.1 ‘ ’ 1

(Dispersion parameter for binomial family taken to be 1)

Null deviance: 86906 on 443107 degrees of freedom

Residual deviance: 81716 on 443089 degrees of freedom

(45112 observations deleted due to missingness)

AIC: 81754

Number of Fisher Scoring iterations: 7

stroke.hd.ischaemic

=========================

Call:

glm(formula = rf, family = binomial(link = "logit"), data = ms,

maxit = 100)

Deviance Residuals:

Min 1Q Median 3Q Max

-0.8455 -0.2282 -0.1719 -0.1260 3.5258

Coefficients:

Estimate Std. Error z value Pr(>|z|)

(Intercept) -8.6161826 0.0951081 -90.594 < 2e-16 ***

rvMPO 0.0063542 0.0398167 0.160 0.873207

PC1 0.0008528 0.0002202 3.873 0.000107 ***

PC2 0.0012121 0.0005200 2.331 0.019746 *

PC3 0.0012926 0.0009292 1.391 0.164196

PC4 0.0073879 0.0012287 6.013 1.82e-09 ***

PC5 0.0065960 0.0013844 4.764 1.89e-06 ***

PC6 -0.0114362 0.0043547 -2.626 0.008635 **

PC7 -0.0027965 0.0025722 -1.087 0.276934

PC8 0.0070289 0.0031850 2.207 0.027322 *

PC9 0.0146938 0.0025605 5.739 9.55e-09 ***

PC10 0.0027169 0.0028655 0.948 0.343060

sexMale 0.4273222 0.0235212 18.168 < 2e-16 ***

age 0.0638537 0.0015370 41.545 < 2e-16 ***

hypertensionTRUE 0.4963911 0.0252138 19.687 < 2e-16 ***

hyperlipidemiaTRUE 0.1464346 0.0226794 6.457 1.07e-10 ***

gly_controlTRUE 1.0326971 0.0598634 17.251 < 2e-16 ***

obesityTRUE 0.2888004 0.0236399 12.217 < 2e-16 ***

smoking_status 0.2953869 0.0158504 18.636 < 2e-16 ***

---

Signif. codes: 0 ‘***’ 0.001 ‘**’ 0.01 ‘*’ 0.05 ‘.’ 0.1 ‘ ’ 1

(Dispersion parameter for binomial family taken to be 1)

Null deviance: 86750 on 443107 degrees of freedom

Residual deviance: 81598 on 443089 degrees of freedom

(45112 observations deleted due to missingness)

AIC: 81636

Number of Fisher Scoring iterations: 7

stroke.srhd.all

=========================

Call:

glm(formula = rf, family = binomial(link = "logit"), data = ms,

maxit = 100)

Deviance Residuals:

Min 1Q Median 3Q Max

-0.8818 -0.3033 -0.2355 -0.1765 3.2995

Coefficients:

Estimate Std. Error z value Pr(>|z|)

(Intercept) -7.5868644 0.0706201 -107.432 < 2e-16 ***

rvMPO -0.0242692 0.0306250 -0.792 0.4281

PC1 0.0006890 0.0001708 4.034 5.48e-05 ***

PC2 0.0007254 0.0003755 1.932 0.0534 .

PC3 0.0010778 0.0006767 1.593 0.1112

PC4 0.0076006 0.0009258 8.210 < 2e-16 ***

PC5 0.0083462 0.0010463 7.977 1.50e-15 ***

PC6 -0.0064235 0.0026562 -2.418 0.0156 *

PC7 -0.0022405 0.0019471 -1.151 0.2499

PC8 0.0025706 0.0021816 1.178 0.2387

PC9 0.0092845 0.0018879 4.918 8.75e-07 ***

PC10 0.0031104 0.0021728 1.431 0.1523

sexMale 0.2909890 0.0175803 16.552 < 2e-16 ***

age 0.0589310 0.0011504 51.226 < 2e-16 ***

hypertensionTRUE 0.4174807 0.0186712 22.360 < 2e-16 ***

hyperlipidemiaTRUE 0.1545496 0.0172304 8.970 < 2e-16 ***

gly_controlTRUE 0.8639947 0.0506351 17.063 < 2e-16 ***

obesityTRUE 0.2844244 0.0180575 15.751 < 2e-16 ***

smoking_status 0.3129733 0.0119724 26.141 < 2e-16 ***

---

Signif. codes: 0 ‘***’ 0.001 ‘**’ 0.01 ‘*’ 0.05 ‘.’ 0.1 ‘ ’ 1

(Dispersion parameter for binomial family taken to be 1)

Null deviance: 135281 on 443107 degrees of freedom

Residual deviance: 128044 on 443089 degrees of freedom

(45112 observations deleted due to missingness)

AIC: 128082

Number of Fisher Scoring iterations: 6

stroke.hd.all

=========================

Call:

glm(formula = rf, family = binomial(link = "logit"), data = ms,

maxit = 100)

Deviance Residuals:

Min 1Q Median 3Q Max

-0.8235 -0.2590 -0.2009 -0.1518 3.3841

Coefficients:

Estimate Std. Error z value Pr(>|z|)

(Intercept) -7.8016107 0.0821747 -94.939 < 2e-16 ***

rvMPO -0.0207322 0.0357111 -0.581 0.56154

PC1 0.0008294 0.0001936 4.283 1.84e-05 ***

PC2 0.0007311 0.0004249 1.721 0.08532 .

PC3 0.0007380 0.0007709 0.957 0.33836

PC4 0.0076125 0.0010884 6.994 2.67e-12 ***

PC5 0.0071796 0.0012248 5.862 4.57e-09 ***

PC6 -0.0114679 0.0037027 -3.097 0.00195 **

PC7 -0.0031149 0.0022822 -1.365 0.17230

PC8 0.0054943 0.0027643 1.988 0.04686 *

PC9 0.0125938 0.0022494 5.599 2.16e-08 ***

PC10 0.0007528 0.0025305 0.297 0.76609

sexMale 0.3083343 0.0205453 15.008 < 2e-16 ***

age 0.0570119 0.0013400 42.547 < 2e-16 ***

hypertensionTRUE 0.4848291 0.0220090 22.029 < 2e-16 ***

hyperlipidemiaTRUE 0.0967296 0.0201382 4.803 1.56e-06 ***

gly_controlTRUE 0.9250345 0.0570274 16.221 < 2e-16 ***

obesityTRUE 0.2090609 0.0212803 9.824 < 2e-16 ***

smoking_status 0.2928283 0.0139901 20.931 < 2e-16 ***

---

Signif. codes: 0 ‘***’ 0.001 ‘**’ 0.01 ‘*’ 0.05 ‘.’ 0.1 ‘ ’ 1

(Dispersion parameter for binomial family taken to be 1)

Null deviance: 105309 on 443107 degrees of freedom

Residual deviance: 100117 on 443089 degrees of freedom

(45112 observations deleted due to missingness)

AIC: 100155

Number of Fisher Scoring iterations: 7

cad.nochronic.withsr

=========================

Call:

glm(formula = rf, family = binomial(link = "logit"), data = ms,

maxit = 100)

Deviance Residuals:

Min 1Q Median 3Q Max

-1.5506 -0.4102 -0.2782 -0.1865 3.2992

Coefficients:

Estimate Std. Error z value Pr(>|z|)

(Intercept) -7.5748651 0.0522576 -144.952 < 2e-16 ***

rvMPO -0.0099881 0.0223783 -0.446 0.655358

PC1 -0.0001952 0.0001579 -1.236 0.216434

PC2 -0.0022836 0.0002756 -8.285 < 2e-16 ***

PC3 0.0061575 0.0004633 13.292 < 2e-16 ***

PC4 0.0048646 0.0005960 8.162 3.28e-16 ***

PC5 0.0067653 0.0007764 8.713 < 2e-16 ***

PC6 -0.0018930 0.0016980 -1.115 0.264935

PC7 -0.0020555 0.0012877 -1.596 0.110434

PC8 0.0048613 0.0014384 3.380 0.000726 ***

PC9 0.0107487 0.0013903 7.731 1.07e-14 ***

PC10 0.0038356 0.0014098 2.721 0.006517 **

sexMale 0.9755996 0.0138984 70.195 < 2e-16 ***

age 0.0576012 0.0008398 68.593 < 2e-16 ***

hypertensionTRUE 0.4576734 0.0139604 32.784 < 2e-16 ***

hyperlipidemiaTRUE 0.7836863 0.0127475 61.478 < 2e-16 ***

gly_controlTRUE 0.9100007 0.0379996 23.948 < 2e-16 ***

obesityTRUE 0.3964749 0.0130677 30.340 < 2e-16 ***

smoking_status 0.3309286 0.0087729 37.722 < 2e-16 ***

---

Signif. codes: 0 ‘***’ 0.001 ‘**’ 0.01 ‘*’ 0.05 ‘.’ 0.1 ‘ ’ 1

(Dispersion parameter for binomial family taken to be 1)

Null deviance: 232155 on 443107 degrees of freedom

Residual deviance: 201670 on 443089 degrees of freedom

(45112 observations deleted due to missingness)

AIC: 201708

Number of Fisher Scoring iterations: 6

cad.nochronic.hd

=========================

Call:

glm(formula = rf, family = binomial(link = "logit"), data = ms,

maxit = 100)

Deviance Residuals:

Min 1Q Median 3Q Max

-1.5138 -0.3956 -0.2687 -0.1814 3.3292

Coefficients:

Estimate Std. Error z value Pr(>|z|)

(Intercept) -7.5675665 0.0539189 -140.351 < 2e-16 ***

rvMPO -0.0003395 0.0230675 -0.015 0.98826

PC1 -0.0004566 0.0001703 -2.682 0.00732 **

PC2 -0.0024025 0.0002906 -8.267 < 2e-16 ***

PC3 0.0064269 0.0004842 13.273 < 2e-16 ***

PC4 0.0052959 0.0006210 8.528 < 2e-16 ***

PC5 0.0068691 0.0008016 8.569 < 2e-16 ***

PC6 -0.0025779 0.0017967 -1.435 0.15135

PC7 -0.0021610 0.0013475 -1.604 0.10878

PC8 0.0041348 0.0015097 2.739 0.00616 **

PC9 0.0120137 0.0014444 8.317 < 2e-16 ***

PC10 0.0042278 0.0014553 2.905 0.00367 **

sexMale 0.9641606 0.0144210 66.858 < 2e-16 ***

age 0.0560461 0.0008674 64.617 < 2e-16 ***

hypertensionTRUE 0.4734385 0.0145067 32.636 < 2e-16 ***

hyperlipidemiaTRUE 0.7727582 0.0132106 58.495 < 2e-16 ***

gly_controlTRUE 0.9146666 0.0386896 23.641 < 2e-16 ***

obesityTRUE 0.3835292 0.0135094 28.390 < 2e-16 ***

smoking_status 0.3301470 0.0090685 36.406 < 2e-16 ***

---

Signif. codes: 0 ‘***’ 0.001 ‘**’ 0.01 ‘*’ 0.05 ‘.’ 0.1 ‘ ’ 1

(Dispersion parameter for binomial family taken to be 1)

Null deviance: 219311 on 443107 degrees of freedom

Residual deviance: 191293 on 443089 degrees of freedom

(45112 observations deleted due to missingness)

AIC: 191331

Number of Fisher Scoring iterations: 6

cad.chronic.withsr

=========================

Call:

glm(formula = rf, family = binomial(link = "logit"), data = ms,

maxit = 100)

Deviance Residuals:

Min 1Q Median 3Q Max

-1.7357 -0.4995 -0.3513 -0.2331 3.1224

Coefficients:

Estimate Std. Error z value Pr(>|z|)

(Intercept) -7.2913139 0.0443945 -164.239 < 2e-16 ***

rvMPO -0.0046564 0.0189372 -0.246 0.80577

PC1 0.0002461 0.0001229 2.002 0.04533 *

PC2 -0.0021224 0.0002268 -9.358 < 2e-16 ***

PC3 0.0062598 0.0003881 16.131 < 2e-16 ***

PC4 0.0045474 0.0005046 9.012 < 2e-16 ***

PC5 0.0057197 0.0006630 8.627 < 2e-16 ***

PC6 -0.0015447 0.0013430 -1.150 0.25005

PC7 -0.0002166 0.0010944 -0.198 0.84309

PC8 0.0032510 0.0011965 2.717 0.00658 **

PC9 0.0096534 0.0011712 8.242 < 2e-16 ***

PC10 0.0062516 0.0012111 5.162 2.45e-07 ***

sexMale 0.8005993 0.0112263 71.314 < 2e-16 ***

age 0.0642684 0.0007150 89.892 < 2e-16 ***

hypertensionTRUE 0.4060389 0.0115297 35.217 < 2e-16 ***

hyperlipidemiaTRUE 0.5903406 0.0106054 55.664 < 2e-16 ***

gly_controlTRUE 0.9957350 0.0345141 28.850 < 2e-16 ***

obesityTRUE 0.5049926 0.0110803 45.576 < 2e-16 ***

smoking_status 0.3237978 0.0074753 43.316 < 2e-16 ***

---

Signif. codes: 0 ‘***’ 0.001 ‘**’ 0.01 ‘*’ 0.05 ‘.’ 0.1 ‘ ’ 1

(Dispersion parameter for binomial family taken to be 1)

Null deviance: 301057 on 443107 degrees of freedom

Residual deviance: 264281 on 443089 degrees of freedom

(45112 observations deleted due to missingness)

AIC: 264319

Number of Fisher Scoring iterations: 6

cad.chronic.hd

=========================

Call:

glm(formula = rf, family = binomial(link = "logit"), data = ms,

maxit = 100)

Deviance Residuals:

Min 1Q Median 3Q Max

-1.7275 -0.4929 -0.3464 -0.2303 3.1458

Coefficients:

Estimate Std. Error z value Pr(>|z|)

(Intercept) -7.3274355 0.0449718 -162.934 < 2e-16 ***

rvMPO -0.0004813 0.0191330 -0.025 0.9799

PC1 0.0001311 0.0001265 1.036 0.3001

PC2 -0.0021428 0.0002327 -9.208 < 2e-16 ***

PC3 0.0064693 0.0003966 16.311 < 2e-16 ***

PC4 0.0047083 0.0005115 9.205 < 2e-16 ***

PC5 0.0057840 0.0006704 8.628 < 2e-16 ***

PC6 -0.0018704 0.0013704 -1.365 0.1723

PC7 -0.0001440 0.0011134 -0.129 0.8971

PC8 0.0026657 0.0012189 2.187 0.0287 *

PC9 0.0103537 0.0011874 8.719 < 2e-16 ***

PC10 0.0064218 0.0012250 5.242 1.59e-07 ***

sexMale 0.7943095 0.0113698 69.861 < 2e-16 ***

age 0.0642439 0.0007240 88.731 < 2e-16 ***

hypertensionTRUE 0.4151001 0.0116903 35.508 < 2e-16 ***

hyperlipidemiaTRUE 0.5888799 0.0107365 54.848 < 2e-16 ***

gly_controlTRUE 1.0086480 0.0346283 29.128 < 2e-16 ***

obesityTRUE 0.5046063 0.0112002 45.053 < 2e-16 ***

smoking_status 0.3236424 0.0075646 42.784 < 2e-16 ***

---

Signif. codes: 0 ‘***’ 0.001 ‘**’ 0.01 ‘*’ 0.05 ‘.’ 0.1 ‘ ’ 1

(Dispersion parameter for binomial family taken to be 1)

Null deviance: 295394 on 443107 degrees of freedom

Residual deviance: 259386 on 443089 degrees of freedom

(45112 observations deleted due to missingness)

AIC: 259424

Number of Fisher Scoring iterations: 6

### Updated score and MPO burden

stroke.srhd.subarachnoid

=========================

Call:

glm(formula = rf, family = binomial(link = "logit"), data = ms,

maxit = 100)

Deviance Residuals:

Min 1Q Median 3Q Max

-0.1804 -0.0943 -0.0815 -0.0708 3.8040

Coefficients:

Estimate Std. Error z value Pr(>|z|)

(Intercept) -6.7726211 0.1890121 -35.832 < 2e-16 ***

updated.nompo 0.0023026 0.0253855 0.091 0.927727

rvMPOrec -0.0452372 0.0972608 -0.465 0.641851

PC1 -0.0002449 0.0005445 -0.450 0.652900

PC2 0.0009178 0.0010853 0.846 0.397772

PC3 -0.0002304 0.0020303 -0.113 0.909647

PC4 0.0099235 0.0029940 3.314 0.000918 ***

PC5 0.0059369 0.0031643 1.876 0.060627 .

PC6 -0.0134918 0.0089123 -1.514 0.130069

PC7 -0.0129419 0.0063402 -2.041 0.041229 *

PC8 -0.0044399 0.0069971 -0.635 0.525733

PC9 0.0006449 0.0055484 0.116 0.907475

PC10 0.0008011 0.0070476 0.114 0.909494

sexMale -0.4434725 0.0536507 -8.266 < 2e-16 ***

age 0.0157267 0.0032334 4.864 1.15e-06 ***

hypertensionTRUE 0.3673888 0.0542689 6.770 1.29e-11 ***

hyperlipidemiaTRUE -0.0460490 0.0539611 -0.853 0.393451

gly_controlTRUE -0.4427197 0.3040722 -1.456 0.145401

obesityTRUE -0.0790576 0.0593826 -1.331 0.183081

smoking_status 0.4098187 0.0347028 11.809 < 2e-16 ***

---

Signif. codes: 0 ‘***’ 0.001 ‘**’ 0.01 ‘*’ 0.05 ‘.’ 0.1 ‘ ’ 1

(Dispersion parameter for binomial family taken to be 1)

Null deviance: 21381 on 443107 degrees of freedom

Residual deviance: 21091 on 443088 degrees of freedom

(45112 observations deleted due to missingness)

AIC: 21131

Number of Fisher Scoring iterations: 9

stroke.hd.subarachnoid

=========================

Call:

glm(formula = rf, family = binomial(link = "logit"), data = ms,

maxit = 100)

Deviance Residuals:

Min 1Q Median 3Q Max

-0.1782 -0.0871 -0.0754 -0.0649 3.8139

Coefficients:

Estimate Std. Error z value Pr(>|z|)

(Intercept) -6.789e+00 2.027e-01 -33.496 < 2e-16 ***

updated.nompo 1.188e-02 2.767e-02 0.429 0.66781

rvMPOrec -2.031e-02 1.042e-01 -0.195 0.84547

PC1 5.556e-05 5.606e-04 0.099 0.92105

PC2 6.969e-04 1.110e-03 0.628 0.53002

PC3 -5.661e-04 2.098e-03 -0.270 0.78733

PC4 9.373e-03 3.159e-03 2.967 0.00300 **

PC5 8.662e-03 3.362e-03 2.577 0.00997 **

PC6 -1.326e-02 9.566e-03 -1.387 0.16559

PC7 -1.155e-02 6.651e-03 -1.737 0.08244 .

PC8 -1.432e-03 7.453e-03 -0.192 0.84765

PC9 8.564e-03 6.215e-03 1.378 0.16823

PC10 2.757e-04 7.516e-03 0.037 0.97074

sexMale -5.021e-01 5.819e-02 -8.629 < 2e-16 ***

age 1.355e-02 3.475e-03 3.900 9.63e-05 ***

hypertensionTRUE 3.644e-01 5.839e-02 6.240 4.37e-10 ***

hyperlipidemiaTRUE -3.967e-02 5.827e-02 -0.681 0.49593

gly_controlTRUE -3.672e-01 3.190e-01 -1.151 0.24969

obesityTRUE -1.167e-01 6.463e-02 -1.806 0.07096 .

smoking_status 4.378e-01 3.716e-02 11.781 < 2e-16 ***

---

Signif. codes: 0 ‘***’ 0.001 ‘**’ 0.01 ‘*’ 0.05 ‘.’ 0.1 ‘ ’ 1

(Dispersion parameter for binomial family taken to be 1)

Null deviance: 18812 on 443107 degrees of freedom

Residual deviance: 18536 on 443088 degrees of freedom

(45112 observations deleted due to missingness)

AIC: 18576

Number of Fisher Scoring iterations: 9

stroke.srhd.intracerebral

=========================

Call:

glm(formula = rf, family = binomial(link = "logit"), data = ms,

maxit = 100)

Deviance Residuals:

Min 1Q Median 3Q Max

-0.2559 -0.1091 -0.0865 -0.0674 3.8221

Coefficients:

Estimate Std. Error z value Pr(>|z|)

(Intercept) -9.2378466 0.1926270 -47.957 < 2e-16 ***

updated.nompo 0.0136225 0.0234998 0.580 0.562126

rvMPOrec -0.0463491 0.0892470 -0.519 0.603528

PC1 0.0006622 0.0004878 1.357 0.174677

PC2 -0.0012710 0.0008189 -1.552 0.120667

PC3 -0.0003909 0.0015303 -0.255 0.798380

PC4 0.0053752 0.0024709 2.175 0.029603 *

PC5 0.0068548 0.0029253 2.343 0.019114 *

PC6 -0.0057530 0.0060060 -0.958 0.338125

PC7 0.0029601 0.0053744 0.551 0.581790

PC8 -0.0029895 0.0057435 -0.521 0.602712

PC9 0.0027005 0.0051023 0.529 0.596609

PC10 -0.0044206 0.0057786 -0.765 0.444274

sexMale 0.2577040 0.0481603 5.351 8.75e-08 ***

age 0.0560354 0.0031563 17.753 < 2e-16 ***

hypertensionTRUE 0.5012763 0.0518080 9.676 < 2e-16 ***

hyperlipidemiaTRUE -0.0855480 0.0481435 -1.777 0.075578 .

gly_controlTRUE 0.4252985 0.1706468 2.492 0.012693 *

obesityTRUE -0.0245522 0.0531307 -0.462 0.644002

smoking_status 0.1291245 0.0340068 3.797 0.000146 ***

---

Signif. codes: 0 ‘***’ 0.001 ‘**’ 0.01 ‘*’ 0.05 ‘.’ 0.1 ‘ ’ 1

(Dispersion parameter for binomial family taken to be 1)

Null deviance: 25042 on 443107 degrees of freedom

Residual deviance: 24359 on 443088 degrees of freedom

(45112 observations deleted due to missingness)

AIC: 24399

Number of Fisher Scoring iterations: 8

stroke.hd.intracerebral

=========================

Call:

glm(formula = rf, family = binomial(link = "logit"), data = ms,

maxit = 100)

Deviance Residuals:

Min 1Q Median 3Q Max

-0.2588 -0.1048 -0.0816 -0.0627 3.8148

Coefficients:

Estimate Std. Error z value Pr(>|z|)

(Intercept) -9.4869282 0.2033666 -46.649 < 2e-16 ***

updated.nompo 0.0057067 0.0243803 0.234 0.81493

rvMPOrec -0.0856608 0.0947185 -0.904 0.36580

PC1 0.0006028 0.0005185 1.163 0.24502

PC2 -0.0011647 0.0008629 -1.350 0.17707

PC3 -0.0009808 0.0016262 -0.603 0.54641

PC4 0.0055714 0.0026424 2.108 0.03499 *

PC5 0.0065206 0.0030730 2.122 0.03385 *

PC6 -0.0068498 0.0066661 -1.028 0.30416

PC7 0.0053477 0.0056483 0.947 0.34375

PC8 -0.0020054 0.0061815 -0.324 0.74562

PC9 0.0014459 0.0053206 0.272 0.78582

PC10 -0.0083569 0.0061023 -1.369 0.17085

sexMale 0.2713783 0.0504734 5.377 7.59e-08 ***

age 0.0578472 0.0033233 17.406 < 2e-16 ***

hypertensionTRUE 0.5697460 0.0548580 10.386 < 2e-16 ***

hyperlipidemiaTRUE -0.1005964 0.0503647 -1.997 0.04579 *

gly_controlTRUE 0.4641618 0.1756857 2.642 0.00824 **

obesityTRUE -0.0569618 0.0559126 -1.019 0.30832

smoking_status 0.1393215 0.0355729 3.917 8.98e-05 ***

---

Signif. codes: 0 ‘***’ 0.001 ‘**’ 0.01 ‘*’ 0.05 ‘.’ 0.1 ‘ ’ 1

(Dispersion parameter for binomial family taken to be 1)

Null deviance: 23183 on 443107 degrees of freedom

Residual deviance: 22487 on 443088 degrees of freedom

(45112 observations deleted due to missingness)

AIC: 22527

Number of Fisher Scoring iterations: 9

stroke.srhd.ischaemic

=========================

Call:

glm(formula = rf, family = binomial(link = "logit"), data = ms,

maxit = 100)

Deviance Residuals:

Min 1Q Median 3Q Max

-0.8482 -0.2285 -0.1719 -0.1259 3.5324

Coefficients:

Estimate Std. Error z value Pr(>|z|)

(Intercept) -8.6281801 0.0950752 -90.751 < 2e-16 ***

updated.nompo 0.0051602 0.0110232 0.468 0.63970

rvMPOrec 0.0008410 0.0415412 0.020 0.98385

PC1 0.0008379 0.0002220 3.775 0.00016 ***

PC2 0.0011933 0.0005199 2.295 0.02172 *

PC3 0.0013179 0.0009289 1.419 0.15596

PC4 0.0074589 0.0012283 6.073 1.26e-09 ***

PC5 0.0066497 0.0013828 4.809 1.52e-06 ***

PC6 -0.0114177 0.0043423 -2.629 0.00855 **

PC7 -0.0027811 0.0025710 -1.082 0.27939

PC8 0.0068813 0.0031802 2.164 0.03048 *

PC9 0.0145485 0.0025563 5.691 1.26e-08 ***

PC10 0.0023606 0.0028601 0.825 0.40918

sexMale 0.4286112 0.0235023 18.237 < 2e-16 ***

age 0.0640279 0.0015361 41.683 < 2e-16 ***

hypertensionTRUE 0.4980215 0.0252006 19.762 < 2e-16 ***

hyperlipidemiaTRUE 0.1487582 0.0226544 6.566 5.15e-11 ***

gly_controlTRUE 1.0282103 0.0598639 17.176 < 2e-16 ***

obesityTRUE 0.2921914 0.0236014 12.380 < 2e-16 ***

smoking_status 0.2952874 0.0158365 18.646 < 2e-16 ***

---

Signif. codes: 0 ‘***’ 0.001 ‘**’ 0.01 ‘*’ 0.05 ‘.’ 0.1 ‘ ’ 1

(Dispersion parameter for binomial family taken to be 1)

Null deviance: 86906 on 443107 degrees of freedom

Residual deviance: 81715 on 443088 degrees of freedom

(45112 observations deleted due to missingness)

AIC: 81755

Number of Fisher Scoring iterations: 7

stroke.hd.ischaemic

=========================

Call:

glm(formula = rf, family = binomial(link = "logit"), data = ms,

maxit = 100)

Deviance Residuals:

Min 1Q Median 3Q Max

-0.8458 -0.2282 -0.1719 -0.1260 3.5301

Coefficients:

Estimate Std. Error z value Pr(>|z|)

(Intercept) -8.6161459 0.0951120 -90.589 < 2e-16 ***

updated.nompo 0.0059250 0.0110437 0.537 0.591611

rvMPOrec 0.0037296 0.0415440 0.090 0.928466

PC1 0.0008360 0.0002220 3.765 0.000166 ***

PC2 0.0012084 0.0005201 2.324 0.020145 *

PC3 0.0012815 0.0009294 1.379 0.167935

PC4 0.0074035 0.0012290 6.024 1.70e-09 ***

PC5 0.0065962 0.0013844 4.765 1.89e-06 ***

PC6 -0.0114395 0.0043553 -2.627 0.008625 **

PC7 -0.0028152 0.0025723 -1.094 0.273770

PC8 0.0070245 0.0031852 2.205 0.027430 *

PC9 0.0146995 0.0025605 5.741 9.42e-09 ***

PC10 0.0027152 0.0028654 0.948 0.343340

sexMale 0.4273464 0.0235213 18.169 < 2e-16 ***

age 0.0638560 0.0015370 41.546 < 2e-16 ***

hypertensionTRUE 0.4963872 0.0252138 19.687 < 2e-16 ***

hyperlipidemiaTRUE 0.1464396 0.0226794 6.457 1.07e-10 ***

gly_controlTRUE 1.0327355 0.0598641 17.251 < 2e-16 ***

obesityTRUE 0.2887902 0.0236399 12.216 < 2e-16 ***

smoking_status 0.2953700 0.0158504 18.635 < 2e-16 ***

---

Signif. codes: 0 ‘***’ 0.001 ‘**’ 0.01 ‘*’ 0.05 ‘.’ 0.1 ‘ ’ 1

(Dispersion parameter for binomial family taken to be 1)

Null deviance: 86750 on 443107 degrees of freedom

Residual deviance: 81598 on 443088 degrees of freedom

(45112 observations deleted due to missingness)

AIC: 81638

Number of Fisher Scoring iterations: 7

stroke.srhd.all

=========================

Call:

glm(formula = rf, family = binomial(link = "logit"), data = ms,

maxit = 100)

Deviance Residuals:

Min 1Q Median 3Q Max

-0.8826 -0.3032 -0.2355 -0.1765 3.3044

Coefficients:

Estimate Std. Error z value Pr(>|z|)

(Intercept) -7.5873561 0.0706236 -107.434 < 2e-16 ***

updated.nompo 0.0089766 0.0084243 1.066 0.286624

rvMPOrec -0.0213846 0.0318670 -0.671 0.502185

PC1 0.0006654 0.0001722 3.865 0.000111 ***

PC2 0.0007177 0.0003756 1.911 0.056018 .

PC3 0.0010632 0.0006768 1.571 0.116205

PC4 0.0076237 0.0009260 8.233 < 2e-16 ***

PC5 0.0083522 0.0010463 7.982 1.43e-15 ***

PC6 -0.0064238 0.0026568 -2.418 0.015612 *

PC7 -0.0022649 0.0019472 -1.163 0.244762

PC8 0.0025673 0.0021818 1.177 0.239327

PC9 0.0092956 0.0018879 4.924 8.49e-07 ***

PC10 0.0031078 0.0021727 1.430 0.152615

sexMale 0.2910295 0.0175804 16.554 < 2e-16 ***

age 0.0589337 0.0011504 51.229 < 2e-16 ***

hypertensionTRUE 0.4174799 0.0186712 22.360 < 2e-16 ***

hyperlipidemiaTRUE 0.1545628 0.0172303 8.970 < 2e-16 ***

gly_controlTRUE 0.8641185 0.0506360 17.065 < 2e-16 ***

obesityTRUE 0.2844135 0.0180575 15.750 < 2e-16 ***

smoking_status 0.3129530 0.0119723 26.140 < 2e-16 ***

---

Signif. codes: 0 ‘***’ 0.001 ‘**’ 0.01 ‘*’ 0.05 ‘.’ 0.1 ‘ ’ 1

(Dispersion parameter for binomial family taken to be 1)

Null deviance: 135281 on 443107 degrees of freedom

Residual deviance: 128043 on 443088 degrees of freedom

(45112 observations deleted due to missingness)

AIC: 128083

Number of Fisher Scoring iterations: 6

stroke.hd.all

=========================

Call:

glm(formula = rf, family = binomial(link = "logit"), data = ms,

maxit = 100)

Deviance Residuals:

Min 1Q Median 3Q Max

-0.8239 -0.2591 -0.2009 -0.1518 3.3895

Coefficients:

Estimate Std. Error z value Pr(>|z|)

(Intercept) -7.8021204 0.0821789 -94.941 < 2e-16 ***

updated.nompo 0.0098807 0.0098453 1.004 0.31558

rvMPOrec -0.0179935 0.0371740 -0.484 0.62836

PC1 0.0008033 0.0001953 4.113 3.91e-05 ***

PC2 0.0007229 0.0004250 1.701 0.08898 .

PC3 0.0007219 0.0007710 0.936 0.34913

PC4 0.0076381 0.0010887 7.016 2.29e-12 ***

PC5 0.0071856 0.0012248 5.867 4.45e-09 ***

PC6 -0.0114712 0.0037036 -3.097 0.00195 **

PC7 -0.0031425 0.0022823 -1.377 0.16855

PC8 0.0054917 0.0027647 1.986 0.04699 *

PC9 0.0126052 0.0022493 5.604 2.09e-08 ***

PC10 0.0007501 0.0025304 0.296 0.76690

sexMale 0.3083767 0.0205454 15.010 < 2e-16 ***

age 0.0570150 0.0013400 42.549 < 2e-16 ***

hypertensionTRUE 0.4848245 0.0220090 22.028 < 2e-16 ***

hyperlipidemiaTRUE 0.0967443 0.0201382 4.804 1.56e-06 ***

gly_controlTRUE 0.9251569 0.0570284 16.223 < 2e-16 ***

obesityTRUE 0.2090497 0.0212803 9.824 < 2e-16 ***

smoking_status 0.2928063 0.0139901 20.930 < 2e-16 ***

---

Signif. codes: 0 ‘***’ 0.001 ‘**’ 0.01 ‘*’ 0.05 ‘.’ 0.1 ‘ ’ 1

(Dispersion parameter for binomial family taken to be 1)

Null deviance: 105309 on 443107 degrees of freedom

Residual deviance: 100116 on 443088 degrees of freedom

(45112 observations deleted due to missingness)

AIC: 100156

Number of Fisher Scoring iterations: 7

cad.nochronic.withsr

=========================

Call:

glm(formula = rf, family = binomial(link = "logit"), data = ms,

maxit = 100)

Deviance Residuals:

Min 1Q Median 3Q Max

-1.5505 -0.4102 -0.2782 -0.1865 3.2992

Coefficients:

Estimate Std. Error z value Pr(>|z|)

(Intercept) -7.5747639 0.0522599 -144.944 < 2e-16 ***

updated.nompo 0.0004515 0.0061288 0.074 0.941279

rvMPOrec -0.0118876 0.0233027 -0.510 0.609955

PC1 -0.0001969 0.0001588 -1.240 0.214963

PC2 -0.0022835 0.0002757 -8.283 < 2e-16 ***

PC3 0.0061562 0.0004634 13.286 < 2e-16 ***

PC4 0.0048660 0.0005962 8.162 3.30e-16 ***

PC5 0.0067646 0.0007764 8.712 < 2e-16 ***

PC6 -0.0018934 0.0016980 -1.115 0.264824

PC7 -0.0020572 0.0012878 -1.597 0.110166

PC8 0.0048601 0.0014384 3.379 0.000728 ***

PC9 0.0107488 0.0013904 7.731 1.07e-14 ***

PC10 0.0038355 0.0014098 2.721 0.006518 **

sexMale 0.9755966 0.0138985 70.194 < 2e-16 ***

age 0.0576015 0.0008398 68.593 < 2e-16 ***

hypertensionTRUE 0.4576748 0.0139604 32.784 < 2e-16 ***

hyperlipidemiaTRUE 0.7836883 0.0127475 61.478 < 2e-16 ***

gly_controlTRUE 0.9100037 0.0379997 23.948 < 2e-16 ***

obesityTRUE 0.3964753 0.0130677 30.340 < 2e-16 ***

smoking_status 0.3309287 0.0087729 37.722 < 2e-16 ***

---

Signif. codes: 0 ‘***’ 0.001 ‘**’ 0.01 ‘*’ 0.05 ‘.’ 0.1 ‘ ’ 1

(Dispersion parameter for binomial family taken to be 1)

Null deviance: 232155 on 443107 degrees of freedom

Residual deviance: 201670 on 443088 degrees of freedom

(45112 observations deleted due to missingness)

AIC: 201710

Number of Fisher Scoring iterations: 6

cad.nochronic.hd

=========================

Call:

glm(formula = rf, family = binomial(link = "logit"), data = ms,

maxit = 100)

Deviance Residuals:

Min 1Q Median 3Q Max

-1.5144 -0.3956 -0.2688 -0.1814 3.3293

Coefficients:

Estimate Std. Error z value Pr(>|z|)

(Intercept) -7.5672141 0.0539209 -140.339 < 2e-16 ***

updated.nompo -0.0028153 0.0063169 -0.446 0.65583

rvMPOrec -0.0038771 0.0240271 -0.161 0.87181

PC1 -0.0004498 0.0001711 -2.629 0.00856 **

PC2 -0.0023994 0.0002907 -8.254 < 2e-16 ***

PC3 0.0064309 0.0004843 13.278 < 2e-16 ***

PC4 0.0052890 0.0006212 8.514 < 2e-16 ***

PC5 0.0068656 0.0008016 8.564 < 2e-16 ***

PC6 -0.0025788 0.0017966 -1.435 0.15118

PC7 -0.0021545 0.0013476 -1.599 0.10988

PC8 0.0041346 0.0015096 2.739 0.00617 **

PC9 0.0120092 0.0014445 8.314 < 2e-16 ***

PC10 0.0042288 0.0014553 2.906 0.00366 **

sexMale 0.9641416 0.0144211 66.857 < 2e-16 ***

age 0.0560447 0.0008674 64.615 < 2e-16 ***

hypertensionTRUE 0.4734466 0.0145068 32.636 < 2e-16 ***

hyperlipidemiaTRUE 0.7727566 0.0132106 58.495 < 2e-16 ***

gly_controlTRUE 0.9146259 0.0386896 23.640 < 2e-16 ***

obesityTRUE 0.3835304 0.0135094 28.390 < 2e-16 ***

smoking_status 0.3301561 0.0090685 36.407 < 2e-16 ***

---

Signif. codes: 0 ‘***’ 0.001 ‘**’ 0.01 ‘*’ 0.05 ‘.’ 0.1 ‘ ’ 1

(Dispersion parameter for binomial family taken to be 1)

Null deviance: 219311 on 443107 degrees of freedom

Residual deviance: 191292 on 443088 degrees of freedom

(45112 observations deleted due to missingness)

AIC: 191332

Number of Fisher Scoring iterations: 6

cad.chronic.withsr

=========================

Call:

glm(formula = rf, family = binomial(link = "logit"), data = ms,

maxit = 100)

Deviance Residuals:

Min 1Q Median 3Q Max

-1.7367 -0.4995 -0.3513 -0.2331 3.1222

Coefficients:

Estimate Std. Error z value Pr(>|z|)

(Intercept) -7.2917887 0.0443972 -164.240 < 2e-16 ***

updated.nompo 0.0080751 0.0052379 1.542 0.12315

rvMPOrec -0.0022598 0.0197252 -0.115 0.90879

PC1 0.0002247 0.0001237 1.816 0.06933 .

PC2 -0.0021293 0.0002269 -9.386 < 2e-16 ***

PC3 0.0062461 0.0003881 16.092 < 2e-16 ***

PC4 0.0045677 0.0005047 9.050 < 2e-16 ***

PC5 0.0057246 0.0006630 8.634 < 2e-16 ***

PC6 -0.0015434 0.0013431 -1.149 0.25051

PC7 -0.0002379 0.0010945 -0.217 0.82793

PC8 0.0032474 0.0011965 2.714 0.00665 **

PC9 0.0096639 0.0011712 8.251 < 2e-16 ***

PC10 0.0062504 0.0012111 5.161 2.46e-07 ***

sexMale 0.8006428 0.0112264 71.318 < 2e-16 ***

age 0.0642722 0.0007150 89.897 < 2e-16 ***

hypertensionTRUE 0.4060310 0.0115298 35.216 < 2e-16 ***

hyperlipidemiaTRUE 0.5903475 0.0106054 55.665 < 2e-16 ***

gly_controlTRUE 0.9958631 0.0345149 28.853 < 2e-16 ***

obesityTRUE 0.5049864 0.0110803 45.575 < 2e-16 ***

smoking_status 0.3237737 0.0074753 43.313 < 2e-16 ***

---

Signif. codes: 0 ‘***’ 0.001 ‘**’ 0.01 ‘*’ 0.05 ‘.’ 0.1 ‘ ’ 1

(Dispersion parameter for binomial family taken to be 1)

Null deviance: 301057 on 443107 degrees of freedom

Residual deviance: 264279 on 443088 degrees of freedom

(45112 observations deleted due to missingness)

AIC: 264319

Number of Fisher Scoring iterations: 6

cad.chronic.hd

=========================

Call:

glm(formula = rf, family = binomial(link = "logit"), data = ms,

maxit = 100)

Deviance Residuals:

Min 1Q Median 3Q Max

-1.7284 -0.4928 -0.3464 -0.2303 3.1456

Coefficients:

Estimate Std. Error z value Pr(>|z|)

(Intercept) -7.3278597 0.0449745 -162.934 < 2e-16 ***

updated.nompo 0.0073081 0.0052952 1.380 0.168

rvMPOrec 0.0017380 0.0199303 0.087 0.931

PC1 0.0001117 0.0001272 0.878 0.380

PC2 -0.0021490 0.0002328 -9.232 < 2e-16 ***

PC3 0.0064569 0.0003967 16.277 < 2e-16 ***

PC4 0.0047267 0.0005116 9.238 < 2e-16 ***

PC5 0.0057884 0.0006704 8.635 < 2e-16 ***

PC6 -0.0018693 0.0013705 -1.364 0.173

PC7 -0.0001633 0.0011134 -0.147 0.883

PC8 0.0026624 0.0012190 2.184 0.029 *

PC9 0.0103632 0.0011874 8.728 < 2e-16 ***

PC10 0.0064206 0.0012249 5.242 1.59e-07 ***

sexMale 0.7943493 0.0113699 69.864 < 2e-16 ***

age 0.0642474 0.0007240 88.735 < 2e-16 ***

hypertensionTRUE 0.4150920 0.0116903 35.507 < 2e-16 ***

hyperlipidemiaTRUE 0.5888849 0.0107366 54.849 < 2e-16 ***

gly_controlTRUE 1.0087614 0.0346290 29.131 < 2e-16 ***

obesityTRUE 0.5046003 0.0112002 45.053 < 2e-16 ***

smoking_status 0.3236197 0.0075646 42.781 < 2e-16 ***

---

Signif. codes: 0 ‘***’ 0.001 ‘**’ 0.01 ‘*’ 0.05 ‘.’ 0.1 ‘ ’ 1

(Dispersion parameter for binomial family taken to be 1)

Null deviance: 295394 on 443107 degrees of freedom

Residual deviance: 259385 on 443088 degrees of freedom

(45112 observations deleted due to missingness)

AIC: 259425

Number of Fisher Scoring iterations: 6

# Supplementary Figures

## Supplementary Figure 1


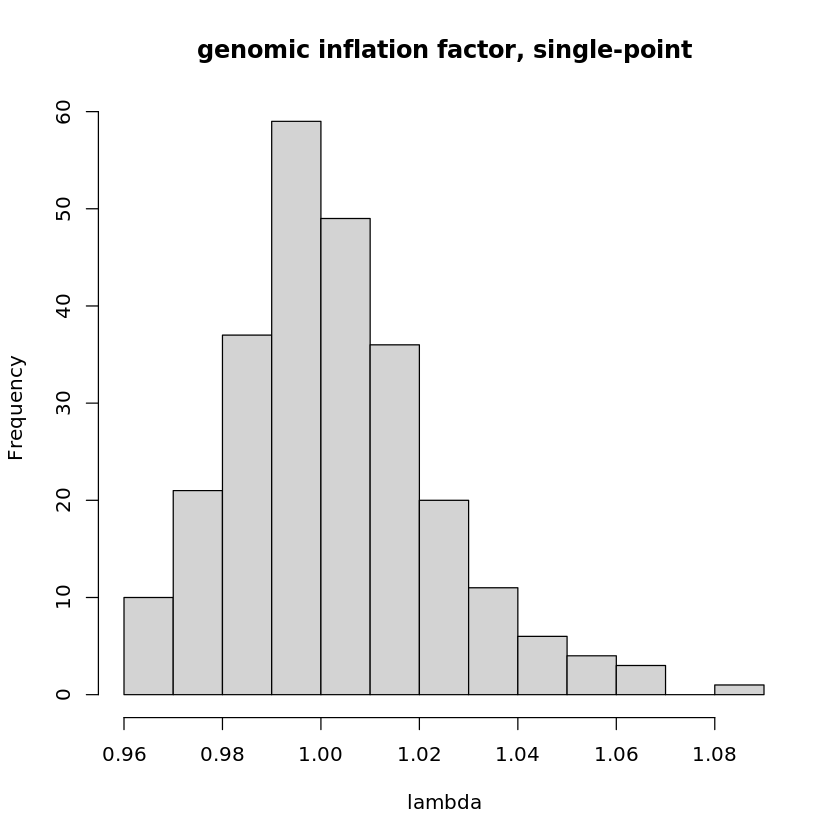


**Supplementary Figure 1: Histogram of single-point association summary statistics across all 250 proteins examined in this study.** The upper outlier is LOX1, upon inspection of the QQ-plot, it was not excluded as it remained well-controlled.

## Supplementary Figure 2

**Supplementary Figure 2**: Contextualisation of the HELIC-ORCADES single-point meta-analysis results. The right column shows the 2013 meta-analyses for serum and plasma MPO, respectively, with their very distinct features. For comparison, the HELIC and ORCADES analyses are displayed next to them. The 3-way reflects the combination of these 2 methods. For reference, the largest MPO study (in plasma) is displayed at the bottom.


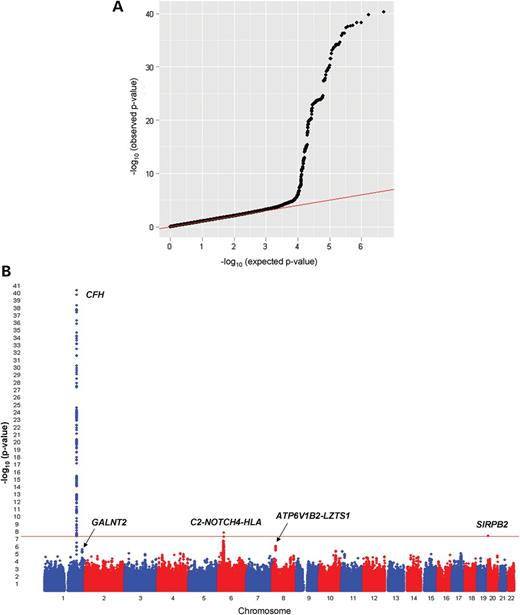

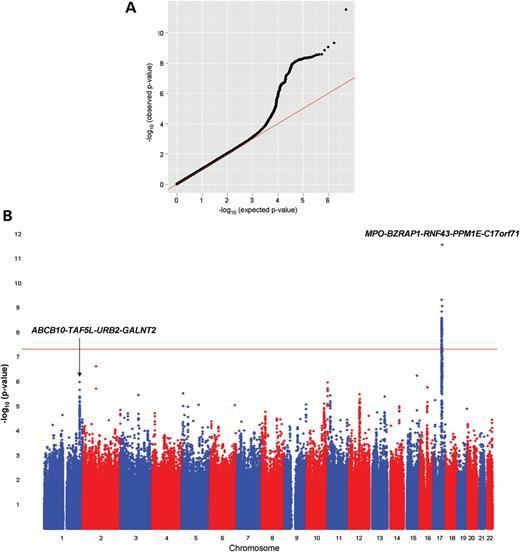


[HELIC 2-way meta-analysis / serum]

[2013 meta-analysis / serum]

[ORCADES analysis - plasma]

[2013 meta-analysis / plasma]

[3-way meta-analysis plasma+serum]

[Folkersen et al meta-analysis / serum]


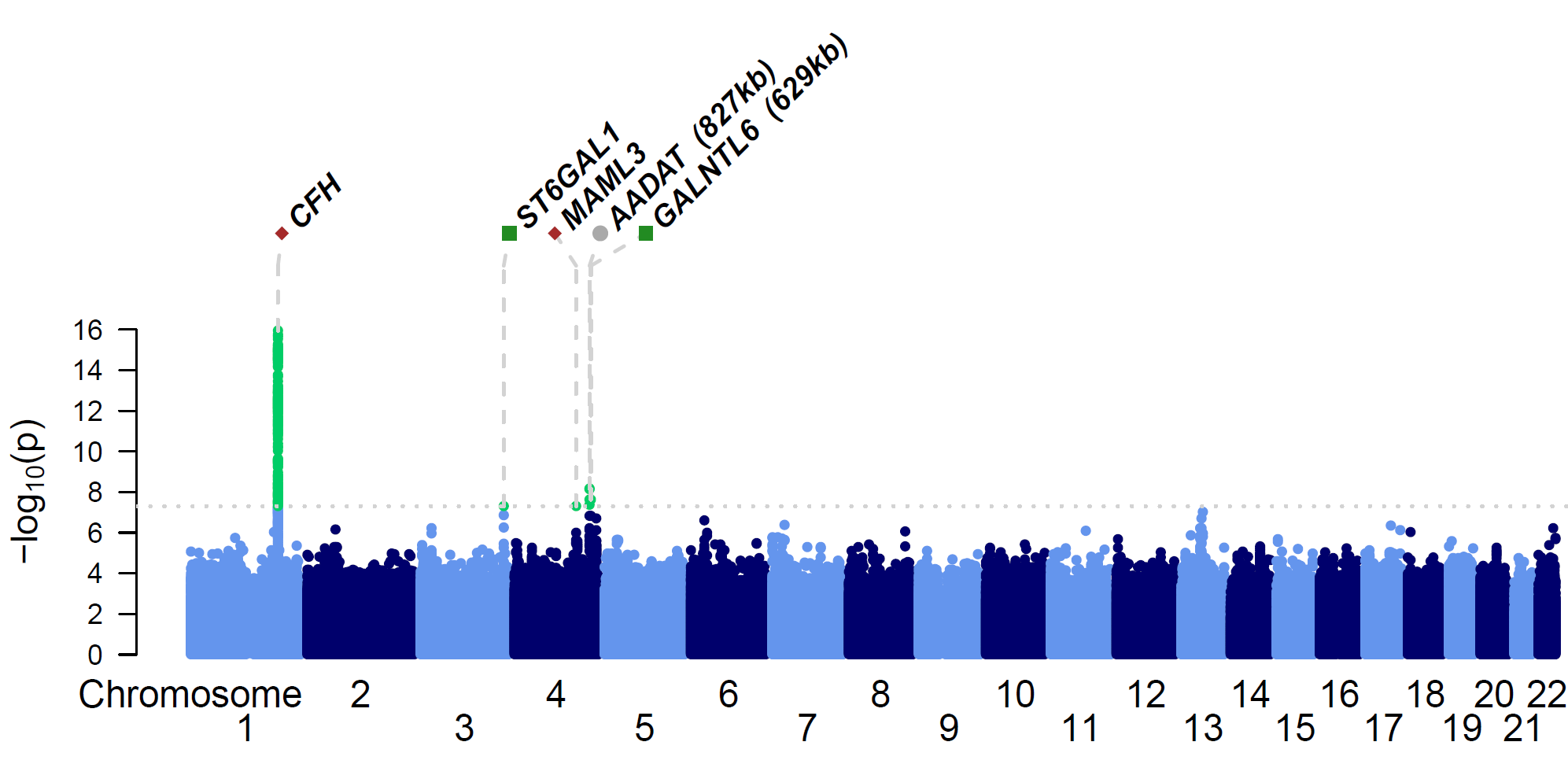

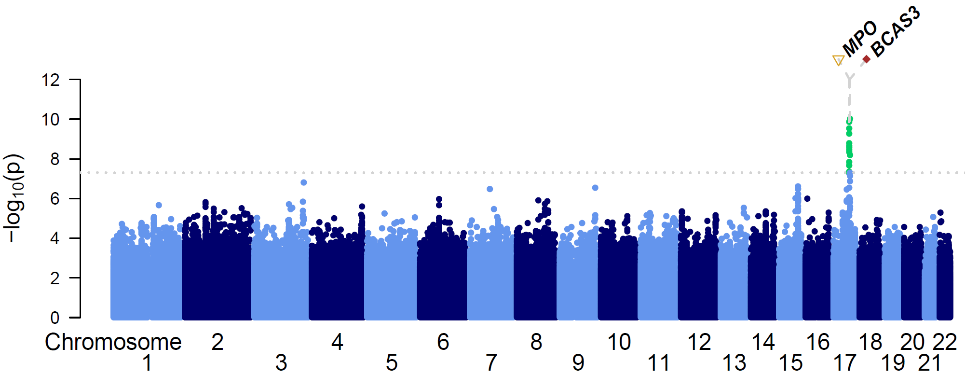

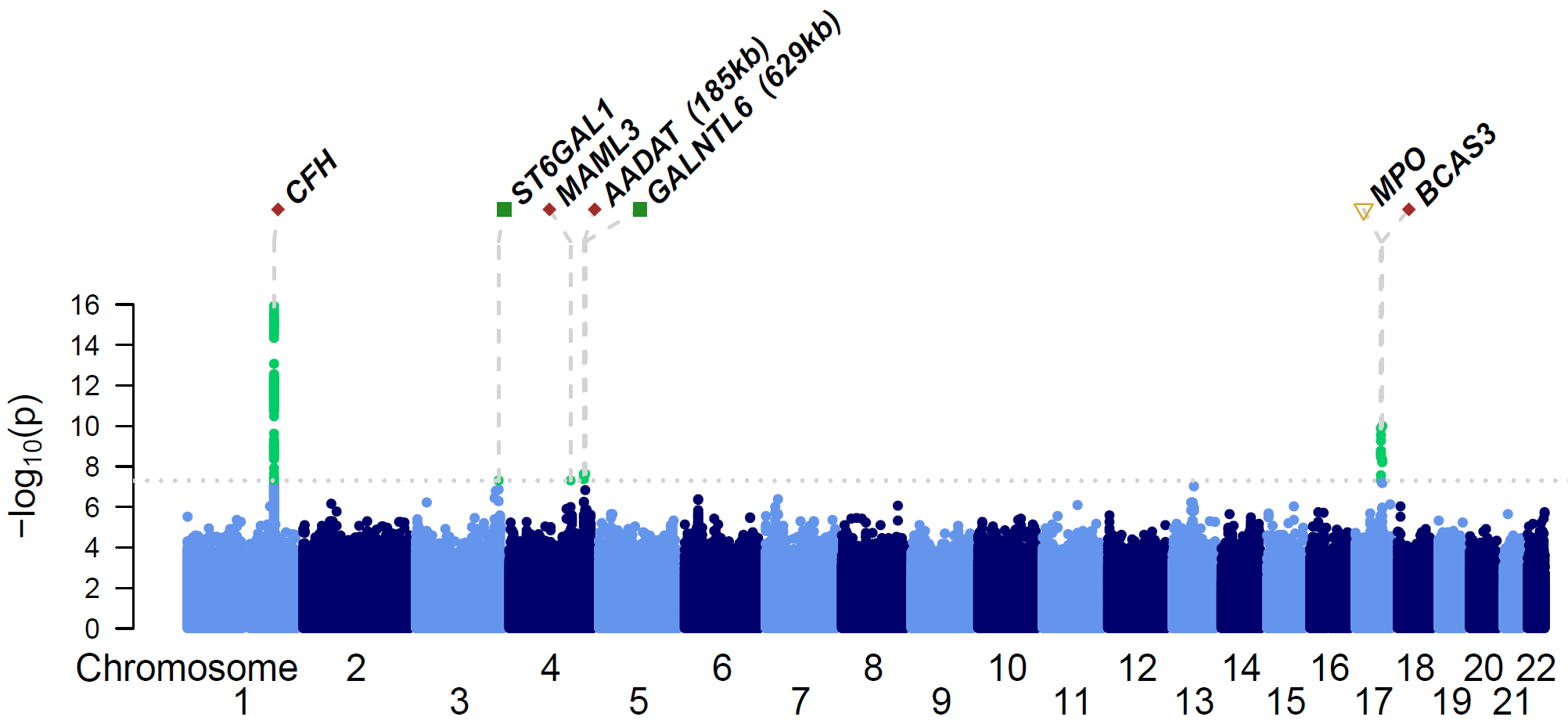

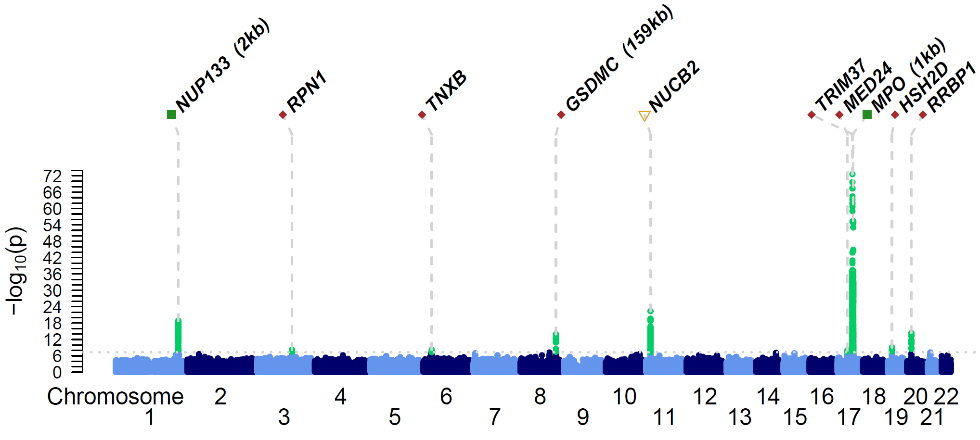


# References

[1] Salmon, R.M., Guo, J., Wood, J.H., Tong, Z., Beech, J.S., Lawera, A., et al., 2020. Molecular basis of ALK1-mediated signalling by BMP9/BMP10 and their prodomain-bound forms. Nat Commun 11(1):1621.

[2] Sanrattana, W., Maas, C., de Maat, S., 2019. SERPINs-From Trap to Treatment. Front Med (Lausanne) 6:25.

[3] Li, W., Long, L., Yang, X., Tong, Z., Southwood, M., King, R., et al., 2021. Circulating BMP9 Protects the Pulmonary Endothelium during Inflammation-induced Lung Injury in Mice. Am J Respir Crit Care Med 203(11):1419-1430.

[4] Gilly, A., Park, Y.C., Png, G., Barysenka, A., Fischer, I., Bjornland, T., et al., 2020. Whole-genome sequencing analysis of the cardiometabolic proteome. Nat Commun 11(1):6336.

[5] Price, S.J., Greaves, D.R., Watkins, H., 2001. Identification of novel, functional genetic variants in the human matrix metalloproteinase-2 gene: role of Sp1 in allele-specific transcriptional regulation. J Biol Chem 276(10):7549-7558.

[6] Yu, C., Zhou, Y., Miao, X., Xiong, P., Tan, W., Lin, D., 2004. Functional haplotypes in the promoter of matrix metalloproteinase-2 predict risk of the occurrence and metastasis of esophageal cancer. Cancer Res 64(20):7622-7628.

[7] Marson, B.P., Lacchini, R., Belo, V., Dickel, S., da Costa, B.P., Poli de Figueiredo, C.E., et al., 2012. Matrix metalloproteinase (MMP)-2 genetic variants modify the circulating MMP-2 levels in end-stage kidney disease. Am J Nephrol 35(3):209-215.

[8] Hua, Y., Song, L., Wu, N., Xie, G., Lu, X., Fan, X., et al., 2009. Polymorphisms of MMP-2 gene are associated with systolic heart failure prognosis. Clin Chim Acta 404(2):119-123.

[9] Gao, R., Yu, H., Zhao, Q., Wang, S., Bai, B., 2019. Role of MMP-2(-1306 C/T) and TIMP-2(-418G/C) Polymorphism in Chinese Han Patients with Acne Vulgaris. Biomed Res Int 2019:2364581.

[10] Li, X., Jin, L., Tan, Y., 2021. Different roles of matrix metalloproteinase 2 in osteolysis of skeletal dysplasia and bone metastasis (Review). Mol Med Rep 23(1).

[11] Morris, J.A., Kemp, J.P., Youlten, S.E., Laurent, L., Logan, J.G., Chai, R.C., et al., 2019. An atlas of genetic influences on osteoporosis in humans and mice. Nat Genet 51(2):258-266.

[12] Quintero-Fabian, S., Arreola, R., Becerril-Villanueva, E., Torres-Romero, J.C., Arana-Argaez, V., Lara-Riegos, J., et al., 2019. Role of Matrix Metalloproteinases in Angiogenesis and Cancer. Front Oncol 9:1370.

[13] Yang, C., Farias, F.H.G., Ibanez, L., Suhy, A., Sadler, B., Fernandez, M.V., et al., 2021. Genomic atlas of the proteome from brain, CSF and plasma prioritizes proteins implicated in neurological disorders. Nat Neurosci 24(9):1302-1312.

[14] Zhang, J., Dutta, D., Köttgen, A., Tin, A., Schlosser, P., Grams, M.E., et al., 2022. Plasma proteome analyses in individuals of European and African ancestry identify <em>cis</em>-pQTLs and models for proteome-wide association studies. bioRxiv.

[15] Reiner, A.P., Hartiala, J., Zeller, T., Bis, J.C., Dupuis, J., Fornage, M., et al., 2013. Genome-wide and gene-centric analyses of circulating myeloperoxidase levels in the charge and care consortia. Hum Mol Genet 22(16):3381-3393.

[16] Phuah, C.L., Dave, T., Malik, R., Raffeld, M.R., Ayres, A.M., Goldstein, J.N., et al., 2017. Genetic variants influencing elevated myeloperoxidase levels increase risk of stroke. Brain 140(10):2663-2672.

[17] Khine, H.W., Teiber, J.F., Haley, R.W., Khera, A., Ayers, C.R., Rohatgi, A., 2017. Association of the serum myeloperoxidase/high-density lipoprotein particle ratio and incident cardiovascular events in a multi-ethnic population: Observations from the Dallas Heart Study. Atherosclerosis 263:156-162.

[18] Duclos, F., Abell, L.M., Harden, D.G., Pike, K., Nowak, K., Locke, G.A., et al., 2017. Triazolopyrimidines identified as reversible myeloperoxidase inhibitors. Medchemcomm 8(11):2093-2099.

[19] Pahwa, R., Modi, P., Jialal, I., 2021. Myeloperoxidase Deficiency. StatPearls: Treasure Island (FL).

[20] Kutter, D., Devaquet, P., Vanderstocken, G., Paulus, J.M., Marchal, V., Gothot, A., 2000. Consequences of total and subtotal myeloperoxidase deficiency: risk or benefit ? Acta Haematol 104(1):10-15.

[21] Zhang, R., Brennan, M.L., Fu, X., Aviles, R.J., Pearce, G.L., Penn, M.S., et al., 2001. Association between myeloperoxidase levels and risk of coronary artery disease. JAMA 286(17):2136-2142.

[22] Meuwese, M.C., Stroes, E.S., Hazen, S.L., van Miert, J.N., Kuivenhoven, J.A., Schaub, R.G., et al., 2007. Serum myeloperoxidase levels are associated with the future risk of coronary artery disease in apparently healthy individuals: the EPIC-Norfolk Prospective Population Study. J Am Coll Cardiol 50(2):159-165.

[23] Hasanpour, Z., Javanmard, S.H., Gharaaty, M., Sadeghi, M., 2016. Association between serum myeloperoxidase levels and coronary artery disease in patients without diabetes, hypertension, obesity, and hyperlipidemia. Adv Biomed Res 5:103.

[24] Daugherty, A., Dunn, J.L., Rateri, D.L., Heinecke, J.W., 1994. Myeloperoxidase, a catalyst for lipoprotein oxidation, is expressed in human atherosclerotic lesions. J Clin Invest 94(1):437-444.

[25] Sugiyama, S., Okada, Y., Sukhova, G.K., Virmani, R., Heinecke, J.W., Libby, P., 2001. Macrophage myeloperoxidase regulation by granulocyte macrophage colony-stimulating factor in human atherosclerosis and implications in acute coronary syndromes. Am J Pathol 158(3):879-891.

[26] Tong, W., Hui, H., Shang, W., Zhang, Y., Tian, F., Ma, Q., et al., 2021. Highly sensitive magnetic particle imaging of vulnerable atherosclerotic plaque with active myeloperoxidase-targeted nanoparticles. Theranostics 11(2):506-521.

[27] Askari, A.T., Brennan, M.L., Zhou, X., Drinko, J., Morehead, A., Thomas, J.D., et al., 2003. Myeloperoxidase and plasminogen activator inhibitor 1 play a central role in ventricular remodeling after myocardial infarction. J Exp Med 197(5):615-624.

[28] Vasilyev, N., Williams, T., Brennan, M.L., Unzek, S., Zhou, X., Heinecke, J.W., et al., 2005. Myeloperoxidase-generated oxidants modulate left ventricular remodeling but not infarct size after myocardial infarction. Circulation 112(18):2812-2820.
